# Reaction Mechanism and Metal Selectivity of Human SAMHD1 Elucidated by QM/MM Calculations

**DOI:** 10.1021/acscatal.5c01682

**Published:** 2025-06-01

**Authors:** Wen-Hao Deng, Harry Lewin, Rong-Zhen Liao, Edina Rosta

**Affiliations:** † Key Laboratory of Material Chemistry for Energy Conversion and Storage, Ministry of Education, Hubei Key Laboratory of Bioinorganic Chemistry and Materia Medica, Hubei Key Laboratory of Materials Chemistry and Service Failure, School of Chemistry and Chemical Engineering, 12443 Huazhong University of Science and Technology, Wuhan 430074, PR China; ‡ Department of Physics and Astronomy, 4919 University College London, Gower Street, London WC1E 6BT, United Kingdom

**Keywords:** enzyme catalysis, reaction mechanism, QM/MM calculations, P−O bond cleavage, metal selectivity

## Abstract

2′-Deoxynucleoside-5′-triphosphate triphosphohydrolases (dNTPases) constitute a crucial enzyme family that plays a pivotal role in antiviral innate immunity. Among these enzymes, human SAMHD1 has emerged as a dNTPase with distinct catalytic properties and active-site architecture. This metalloenzyme regulates cellular dNTP concentration through its ability to hydrolyze all four canonical dNTPs into their corresponding 2′-deoxynucleosides and inorganic triphosphates, a reaction requiring coordinated iron and magnesium ions for enzymatic activity. In the present work, molecular dynamics (MD) simulations and quantum mechanics/molecular mechanics (QM/MM) calculations are employed to investigate the mechanistic details of dATP hydrolysis mediated by two metal ions. Starting from the resolved crystal structure, Model-1, containing a Fe^2+^ in the active site, was constructed. Our calculations demonstrate that SAMHD1 employs a bridging hydroxide anion OH^–^ to attack the Pα site of dNTP, triggering the cleavage of the Pα–O5′ bond via a trigonal-bipyramidal transition state. Simultaneously, His215 donates a proton to O5′ of the leaving group, leading to the formation of 2’-deoxyadenosine and triphosphate ion. It is further demonstrated that the native Fe^2+^–Mg^2+^ bimetallic center help catalyze this hydrolysis reaction with a barrier of 13.4 kcal/mol, while the substitution from Fe^2+^ to Fe^3+^ abolishes the catalytic activity of SAMHD1. The comparison between different QM/MM models highlight the high affinity of SAMHD1 for Fe^2+^ relative to Mn^2+^ and Mg^2+^ at one of the bimetallic sites. In addition, the metal ion swapping between Fe^2+^ and Mg^2+^ from their crystallographic positions is shown to elevate the energy of the reactant state, underscoring the critical influence of the metal coordination geometry on catalytic activity. These computational insights not only expand the understanding of how SAMHD1 wisely modulates catalytic reactivity and metal selectivity by binding suitable metal ions but also provide a valuable foundation for guiding the design of drugs for antiviral therapies.

## Graphical abstract

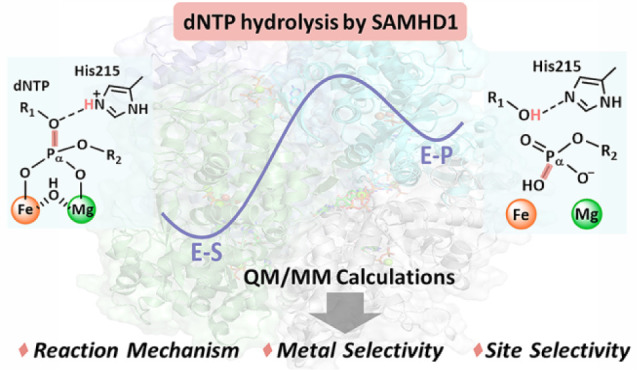

## 1 Introduction

Deoxynucleotide triphosphates (dNTPs) are the building blocks for DNA synthesis and repair. There are four types of dNTP, each containing a different DNA base group: adenine (dATP), cytosine (dCTP), guanine (dGTP), and thymine (dTTP). Absolute and relative concentrations of these four deoxynucleoside triphosphates (dNTPs) should be carefully balanced and tightly regulated within the cell to ensure that DNA replication proceeds efficiently and with high fidelity.^1,2^ Furthermore, in most living organisms, the regulation of intracellular dNTP pools plays a crucial role in antiviral mechanisms. For example, in terminally differentiated cells of the myeloid lineage and resting T-cells, depletion of the dNTP pools to a certain threshold with the help of various enzymes has been found to inhibit the replication of human immunodeficiency virus-1 (HIV-1) and other retroviruses.^3–6^

Sterile alpha motif and HD domain-containing protein 1 (SAMHD1) is a human deoxynucleoside triphosphate triphosphohydrolase.^7,8^ It plays an important role in determining the cellular dNTP pools by hydrolyzing all four dNTPs (see Scheme 1).^8^ SAMHD1 comprises an *N*-terminal nuclear localization signal and two significant structural domains: the sterile alpha motif (SAM) domain, which serves as a helical scaffold for binding proteins or nucleic acids, and the HD domain, named for its two pairs of conserved histidine and aspartate residues that coordinate a metal ion at the active site (see Figure 1).^9,10^ It is worth noting that the hydrolysis of dNTPs in SAMHD1 requires the precise assembly of SAMHD1 into homotetramer.^11^ Additionally, the four pairs of allosteric sites AL1 and AL2 are located in the interfaces between each two monomers and play a crucial role in influencing enzyme activity through the combined binding of one GTP or dGTP molecule, one dNTP molecule, and one magnesium ion (see Figure S2).^5–8,12–15^ Inspection of the resolved crystal structure of human SAMHD1 (PDB Code 6TX0)^8^ in complex with the inhibitor 5′-(α,β-imido)­triphosphate dAMPNPP reveals that the α-phosphate of the substrate is positioned near the HD motif-coordinated iron ion at the A site and a magnesium ion at the B site, while the β- and γ-phosphates are coordinated by another magnesium ion (see Figure 1B). Furthermore, at the Fe–Mg bimetallic center, an oxygen atom, presumably a hydroxide bridges the two metal ions and has been proposed to serve as a nucleophile.^8^ In the previously proposed S_N_2 hydrolysis mechanism displayed in Figure 1C,^8,16,17^ after the nucleophile hydroxide anion attacks the Pα site of dNTP and triggers the cleavage of Pα–O5′ bond via a trigonal-bipyramidal transition state, double-protonated His215 donates a proton to O5′ of the leaving group, leading to the formation of 2’-deoxynucleoside molecule and triphosphate.

**Scheme 1:**
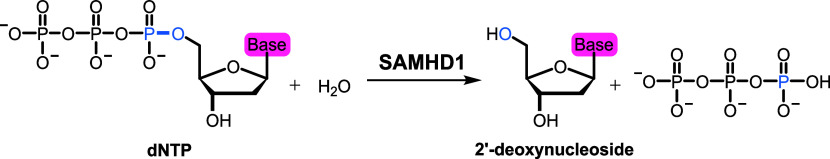
Hydrolysis of dNTP Catalyzed by SAMHD1^a^

a The Pα–O bond highlighted in light blue is cleaved in this reaction. There are four types of dNTP, with each using a different base group (see Figure S1): adenine (dATP), cytosine (dCTP), guanine (dGTP), and thymine (dTTP).

**Figure 1:**
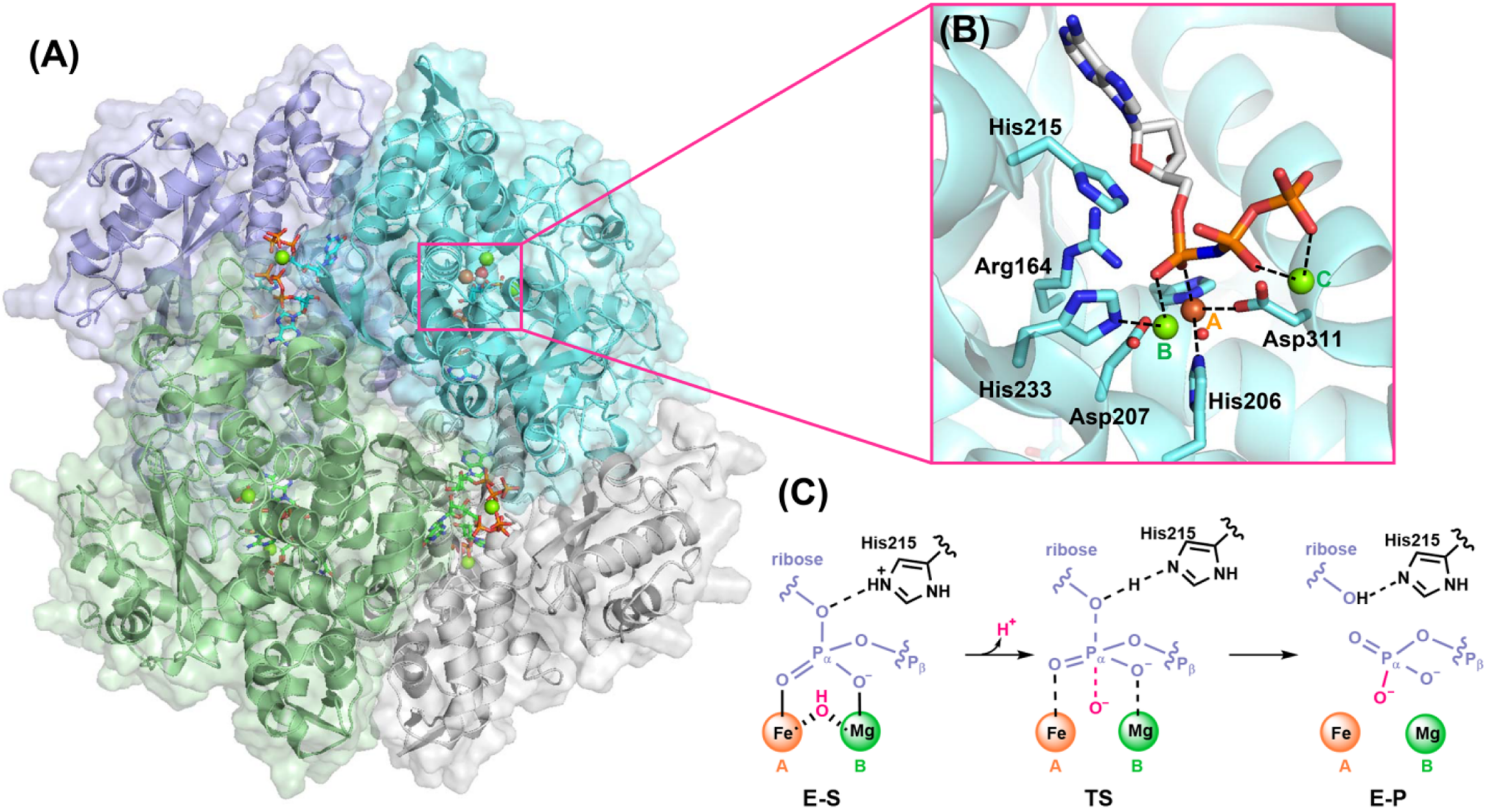
(A) Overall view of the tetramer structure of SAMHD1. Coordinates from the PDB code 6TX0 were used to generate this picture. (B) The active site of SAMHD1. Three metal sites, A, B, and C, are labeled and highlighted in orange and green, respectively. In the crystal structure, Fe is located in site A, while Mg is in sites B and C. His206 and Asp207 are two conserved HD motif residues. (C) A proposed concerted reaction mechanism of dNTP hydrolysis catalyzed by SAMHD1. The reaction proceeds via a nucleophilic attack of a bridging hydroxide on the α-phosphate of the dNTP in the enzyme–substrate complex (E-S), forming a transition state (TS). Then His215 donates a proton to the leaving nucleoside group to form the enzyme–product complex (E-P).

Although many crystal structures of SAMHD1 have been well resolved and reported,^3,7,12,15,16,18^ several key details about the reaction mechanism are still elusive. Taylor et al. proposed that dNTP hydrolysis in SAMHD1 initiates with a nucleophilic attack on the Pα atom by the bridging hydroxide anion (see Figure 1C),^8^ but it remains unclear whether the cleavage of the Pα–O5′ bond and the protonation of the leaving group occur in a concerted or stepwise manner (see Figure S3).^19–22^ Futhermore in the HD-domain enzyme family,^9,10^ apart from several diiron-dependent oxygenases like PhnZ^23^ and MIOX (see Figures S4 and S5),^24^ Fe has been rarely found to be used to catalyze phosphate hydrolysis reaction in the HD domain family.^9^ And most phosphohydrolases in this enzyme family usually utilize Mg,^25^ Mn,^26^ Zn,^27^ or Co^28,29^ to catalyze the cleavage of P–O bond.^9,30^ However, SAMHD1 appears chemically distinct and, to our knowledge, is the first reported phosphohydrolase that employs Fe and Mg to construct a bimetallic center to activate P–O bond.^9^ In contrast to the diiron-dependent oxygenases PhnZ^31^ and MIOX,^32^ which employ iron ions for oxygen binding, SAMHD1 lacks available coordination sites at the Fe center for the binding of additional water or oxygen molecules (see Figure S4). The HD domain monophosphatases OxsA and YfbR recruit only a single metal ion, Mg or Co ion, via their HD motifs, which contribute to Pα–O bond activation and active-site assembly. This metal coordination mode in the monophosphatases differs markedly from the active-site architecture observed in the triphosphatase SAMHD1 (see Figures S5 and S6). On the other hand, the experimental observations indicate that, compared to the Fe ion in the active site, Mg ion in SAMHD1 is more easily substituted by the Mn ion, and the resulting Fe–Mn bicenter remains capable of supporting dNTP hydrolysis with comparable catalytic parameters.^8^ This may underscore the critical role of Fe ions in the enzymatic reaction. However, no clear explanation is currently available for this phenomenon, particularly regarding the preferential substitution of hard Lewis acid Mg ion by soft Lewis acid Mn ion in the active site, rather than by the Fe ion.^33,34^

Despite the importance of SAMHD1, a canonical triphosphatase from the HD domain family, no molecular simulations are available to study this enzyme. Over the past two decades, the quantum mechanics/molecular mechanics (QM/MM) methods^35–38^ have been used to investigate the reaction mechanisms of cyclic nucleotide phosphodiesterases (PDEs), a subgroup of the HD domain superfamily,^21,22,25^ providing many valuable insights into metal-dependent enzymatic P–O bond cleavage of phosphate. For example, Salter and Wierzbicki investigated the catalytic mechanism of 3′,5′-cyclic monophosphate nucleotide hydrolysis by Zn–Mg PDE and proposed that the cleavage of P–O bond and protonation of a leaving group could take place simultaneously.^21^ However, their calculations relied on a density functional theory (DFT) methodology with a small basis set, which is known to predict a pentacoordinate phosphate intermediate that is either not observed or unstable when larger basis sets are employed.^39,40^ Therefore, no current work is available that could correctly and systematically assess the mechanism of P–O bond cleavage catalyzed by enzymes in the HD domain superfamily. Apart from PDEs, while involving a different protein fold, an analogous triphosphatase enzyme reaction is carried out by purple acid phosphatases, a part of the binuclear metallohydrolases superfamily.^41^ For this family of enzymes, Russo et al. revealed a classic stepwise S_N_2 mechanism of monophosphate hydrolysis catalyzed by Zn–Fe purple acid phosphatase from red kidney beans (see Figure S7).^19^

In this study, we utilized molecular dynamics (MD) simulations and QM/MM calculations to advance our mechanistic understanding of the reaction mechanism and metal selectivity of SAMHD1. Our QM/MM calculation results not only closely align with experimental observations but also shed light on SAMHD1’s preference for Fe as one of the catalytic metals. We also find that the reaction mechanism involves a concerted proton transfer coupled with triphosphate cleavage at the rate-limiting step of the reaction, and the bridging hydroxide group does not donate a proton when attacking the triphosphate. This unusual concerted mechanism expands our understanding of the cleavage of the P–O bond of phosphate.

## 2 Methods

### 2.1 System Preparation

The enzyme model was constructed using the crystal structure of tetrameric human D137N-SAMHD1 (PDB code 6TX0) at a resolution of 2.01 Å.^8^ The nucleotide xanthosine-5′-triphosphate (XTP) in the allosteric site AL1 was replaced by GTP, which is the physiological ligand in SAMHD1,^8^ while the original substrate adenosine 5′-(α,β-imido)­triphosphate dAMPNPP in the other allosteric site AL2 (see Figure S2) and the active site was replaced by dATP. Besides, the mutated residue Asn137 in the resolved crystal structure was reverted to its native Asp137 using CHARMM-GUI.^42,43^ The Fe–Mg-bridging oxygen atom was set to be a hydroxide anion according to the previous computational studies on the members of HD domain family^21,25,31,32,44,45^ and molecular modeling of hydrolysis catalyzed by dimetal-containing enzymes.^46–48^ For all four chains, the protonation states of all aspartic and glutamic acid residues, histidine, arginine, and lysine residues in SAMHD1 were calculated using the PROPKA 3.1 code^49^ and then visually checked using Visual Molecular Dynamics (VMD) program.^50^ Based on the predicted p*K*_a_ value (8.75), His215 was set to be protonated (see Table S1). Notably, the substrates dATP and GTP were predicted to be deprotonated, and their charges are −4.

The model was set up using CHARMM-GUI.^42,43^ The molecular mechanics parameters for all the amino acid residues and GTP in SAMHD were taken from the CHARMM36m force field.^51^ The parameters and atomic charges of the substrate dATP were assigned by analogy to ATP, which is available in the force field. All missing hydrogen atoms in the crystal structure were automatically added according to the predicted protonation states.^42^ The system was subsequently solvated in a pre-equilibrated TIP3P^52^ water box with dimensions of 120 Å. Subsequently, it was neutralized by adding counterions KCl and at the same time some TIP3P water molecules were randomly replaced when adding KCl. The final model constructed using CHARMM-GUI consists of 201597 atoms, including 1944 amino acid residues, 56409 TIP3P water molecules (including 193 crystal water molecules), 73 chloride ions, 89 potassium ions, and the substrates dATP and GTP (Figure S8).

### 2.2 Molecular Dynamics Simulation

After the system containing Fe^2+^ and Mg^2+^ ions was constructed, the molecular dynamics (MD) simulations of SAMHD1 in complex with dATP were carried out using GROMACS 2022.4 software^53^ with the CHARMM36m force field.^43,51,54^ To maintain the equilibration of the energy-minimized systems, the temperature and pressure of the systems were maintained. We employed the Nose–Hoover algorithm^55^ to enforce a constant temperature of 303.15 K, while the Parrinello–Rahman pressure-coupling algorithm^56^ was adopted to maintain a pressure of 1.0 bar during the simulation. The system was energy-minimized for 5000 steps using the steepest descent method, and further equilibrations were continued for 1 ns with the NVT ensemble. During the equilibrations, positional restraint potentials were applied and their force constants were gradually reduced. Subsequently, a 25 ns equilibration was performed by using the NPT ensemble. Following this 25 ns NPT equilibration, three independent 100 ns production runs were conducted in the NPT ensemble, starting from the same equilibrated structure. The time step was set as 2 fs throughout the simulation. The particle mesh Ewald (PME) method^57,58^ was employed to handle long-range electrostatic interactions, and the LINCS algorithm^59^ was used to constrain hydrogen atom bonds. All the investigations of MD trajectories were carried out using VMD 1.9.3.^50^ The three production trajectories were analyzed using the Python-based MDAnalysis package (see Figure S9),^60,61^ and the results show that the substrate dATP maintained a stable conformation within the binding pocket, with minimal fluctuations observed. Finally, a final equilibrated structure from one of the 100 ns production runs was selected as a representative model to perform the following QM/MM calculations.

We also considered different metal ion substitutions which were found to have an influence on SAMHD1’s efficiency.^16^ While the metal coordination in HD domain proteins is highly conserved and aligns well even across different enzyme members (see Figures S4 and S5), some structural fluctuations could affect the active site upon replacing the Fe^2+^ ions, which might not be overcome by QM/MM minimization. To test if such flexibility is present at the 100 ns time scale, we used different metals to replace Fe^2+^ at site A in the four chains of SAMHD1 to construct the other three systems, Fe^3+^–Mg^2+^, Mg^2+^–Mg^2+^, and Mn^2+^–Mg^2+^, and then performed additional MD simulations for these systems. Analysis of the 100 ns production MD trajectories revealed that both the O1–Pα and O5′–Pα bond distances maintained dynamic stability (see Figure S10), exhibiting similarity with the Fe^2+^–Mg^2+^-containing SAMHD1 system. These consistent distributions of key chemical bond distances strongly support the idea that QM/MM minimizations can be used to assess the effect of ion replacements on the catalytic barrier. Nevertheless, it remains to be investigated how metal ions and ligands could enter the binding site, and the effects of these processes on the catalytic turnover are currently not known.

### 2.3 QM/MM Methodology

To carry out the following QM/MM calculations, we used the CHARMM36m force field and interfaced with Q-Chem 5.2.^62–64^ Compared with the other chains in the model, chain A was selected, as the substrate dATP is in a better binding mode, to be used to simulate the enzymatic reaction. The full equilibrated system was trimmed to a sphere of 25 Å centered on the Fe in chain A, and this small part, where amino acid residues at the boundary were retained as complete units, consists of 7758 atoms and was partitioned into QM and MM parts, as illustrated in Figure S11. The QM region was chosen to contain 91 atoms, including Fe; two magnesium ions (see Figure 2); a bridging hydroxide group, O1H1; and the side chains of six critical active site residues: positively charged His215, negatively charged Asp311 and Asp207, and neutral His167, His206, and His233. The QM region also included six water molecules: two coordinated with Mg at site B and four coordinated with another Mg at site C. The substrate dATP was split with the nitrogenous base adenine and the five-membered ring of the sugar ribose excluded from the QM region (see Figure 2B). Initially, the charge of the Fe ion was set to be +2 so that the net total charge of the QM region was zero. Fe^3+^ was also considered in the QM/MM calculations and is discussed in Section 3.2. The remaining atoms treated with molecular mechanics were assigned to the MM region. Atoms farther than 20 Å away from the Fe ion were kept frozen during the QM/MM optimizations. We used full electrostatic coupling^65^ between the QM and MM regions and standard link atom treatment for the bonds cut across the MM and QM regions. Seven hydrogen link atoms were placed between the Cβ and Cα bonds for Asp207, Asp311 and the other four histidine residues in the QM region, as well as between the C5′ and C4’ bond for dATP. These hydrogen link atoms were restrained between the original bond partners, and their bond length was scaled by a factor of 0.7261.

**Figure 2:**
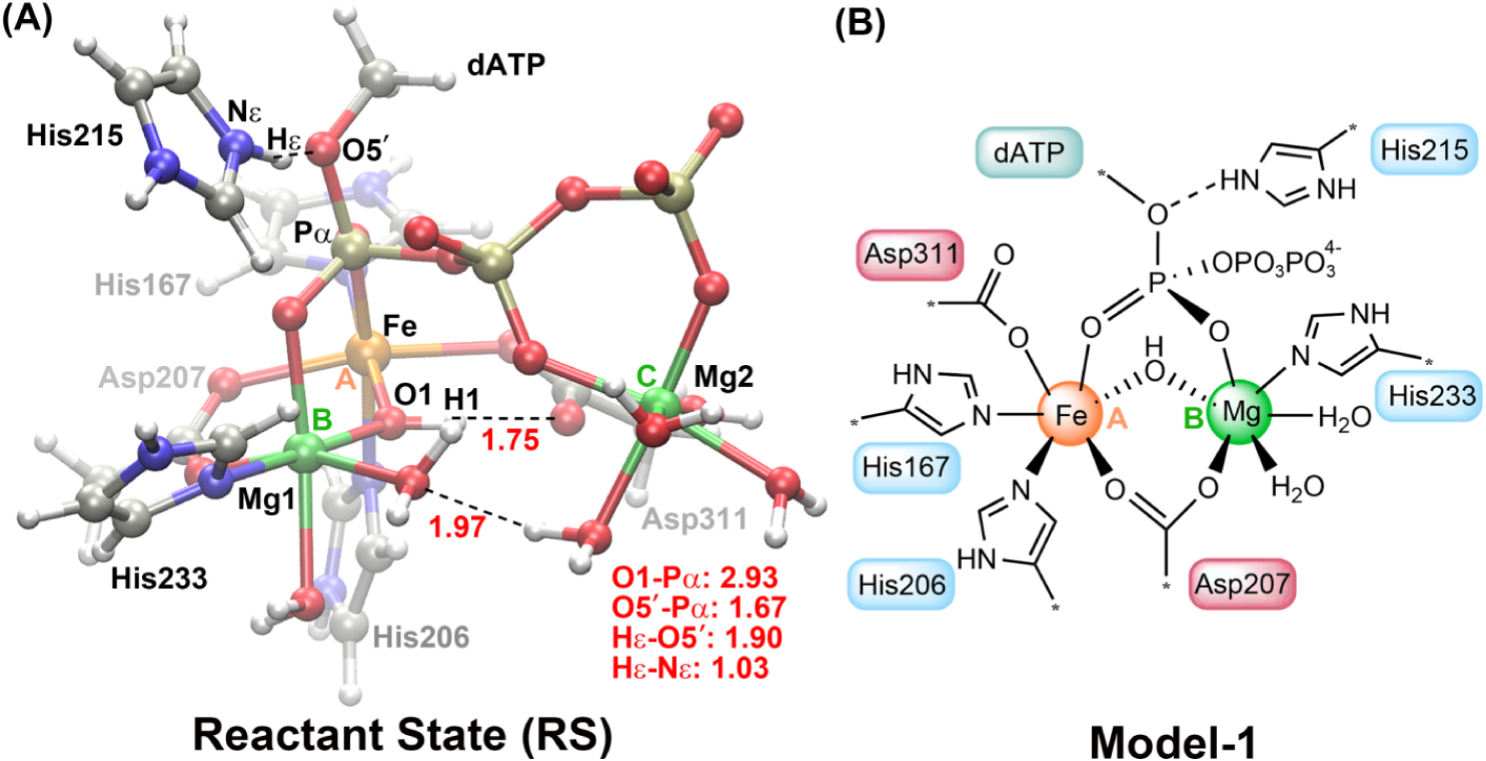
(A) Optimized structure of Reactant State (RS) at the B3LYP/def2-SVP:MM level. Key distances are shown in the lower right corner (red in Å). Two key hydrogen bonds are represented by dashed lines (red in Å). (B) Schematic illustration of the selected QM region of Model-1. Two metal sites, sites A and B, are labeled and highlighted in orange and green, respectively. For clarity, the second Mg^2+^ at site C and its ligands are not shown here. All truncations originating from selecting the QM region are marked with asterisks.

The QM/MM geometry optimizations were performed using the B3LYP functional^66^ and the def2-SVP^67^ basis set for all elements, while the final electronic energies, natural charges on all QM atoms, and spin densities on Fe were calculated by single-point QM/MM calculations at the wB97M-V/def2-TZVP:MM level.^68^ Potential energy surface (PES) scans were carried out in detail along selected reaction coordinates. The reaction coordinate includes Pα–O5′ bond cleavage as well as the proton transfer between dATP and protonated His215 in SAMHD1 (see Figure S12).

## 3 Results and Discussion

We first present a detailed study of the reaction mechanism of SAMHD1 in Section 3.1, followed by an investigation of the catalytic ability of SAMHD1 to utilize Fe^3+^ (see Section 3.2). Next, we compare the catalytic efficiencies of Fe^2+^ and other metal ions based on the proposed reaction pathways to elucidate the origin of the metal selectivity (see Sections 3.3 and 3.4).

### 3.1 Reaction Mechanism

In the optimized geometry of the reactant state complex **RS**, based on the selected snapshot, Fe and Mg ions exhibit regular octahedral coordination, and both are surrounded by six ligands. These ligands include three bridging groups: Asp207, the hydroxide group O1H1, and dATP (see Figure 2). In this model, labeled as Model-1, Fe, as a ferrous iron at the active site, is coordinated by His167, His206, and Asp311, while Mg1, the Mg^2+^ at site B, is ligated by His233 and two water molecules. His233 is vital for stabilizing the binding of the Mg^2+^ ion at site B in SAMHD1, as neither the conserved His nor the metal ion at this site are observed in some other HD domain phosphatases and triphosphatases, such as 2′-deoxyadenosine monophosphatase YfbR (PDB Code 2PAU, see Figure S5) and phosphohydrolase OxsA (PDB Code 5TK8).^17,18,28,29^ The bridging hydroxide group O1H1 is oriented toward the carboxylate group of Asp311. This strong hydrogen bond between Asp311 and OH^–^ increases the nucleophilicity of O1. As shown in Figure 2, the *pro*-R_P_ oxygen on the Pα of dATP is bonded to Fe, whereas the *pro*-S_P_ oxygen is bonded to Mg1. The two active-site metal ions facilitate a shorter distance between the Pα atom and O1 of the hydroxide group to 2.93 Å in **RS**. The hydroxide is perfectly positioned for an in-line attack, with an O1–Pα–O5′ angle of 174.3°. Mg^2+^ at site C is coordinated by oxygen atoms from the Pβ and Pγ moieties of dATP. These interactions effectively assist the nucleophilic hydroxide to attack Pα–O5′ bond of dATP (see Figure S13).

We optimized the **RS** in three different spin states: singlet, triplet, and quintet (see Table S2). We found the quintet state to be the ground state featuring a high-spin Fe^2+^, which has a Mulliken spin density calculated to be 3.81. The singlet and triplet states are energetically higher by ∼20 kcal/mol, demonstrating that the Fe ion in SAMHD1 favors a high-spin state, similar to a binuclear center in the metallohydrolase plant purple acid phosphatase from red kidney beans (rkbPAP),^41^ which also utilizes high-spin iron to catalyze the hydrolysis of phosphomonoesters.^19^ Consequently, the open-shell quintet state is considered for all structures shown below.

A detailed mechanism of SAMHD1 was obtained according to the potential energy scanning results. Here, the nucleophile O1H1 anion attacks Pα of dATP in a directional manner, accompanied by a proton transfer from double-protonated His215 to 2′-deoxyribose sugar moiety. Figure 3A depicts the optimized transition state geometry, which exhibits a pentacoordinate phosphorus coordination at the Pα center. Notably, two critical bond distances emerge in this transition state: the forming Pα–O1 bond (1.80 Å) and the breaking Pα–O5′ bond (1.99 Å), showing a classic concerted but associated catalytic behavior in this triphosphohydrolase (see Figure 4).^69^ In **TS**, the O5′–Hε bond length is 1.26 Å and almost equal to the Nε–Hε bond (1.27 Å, see Figure 3A), indicating that the cleavage/formation of the P–O bonds and the proton transfer from His215 to O5′ are concerted, which is similar to the two-metal-mediated hydrolysis reactions by cGMP-specific phosphodiesterase-5 ^22^ and Pseudomonas diminuta phosphotriesterase.^20^

**Figure 3:**
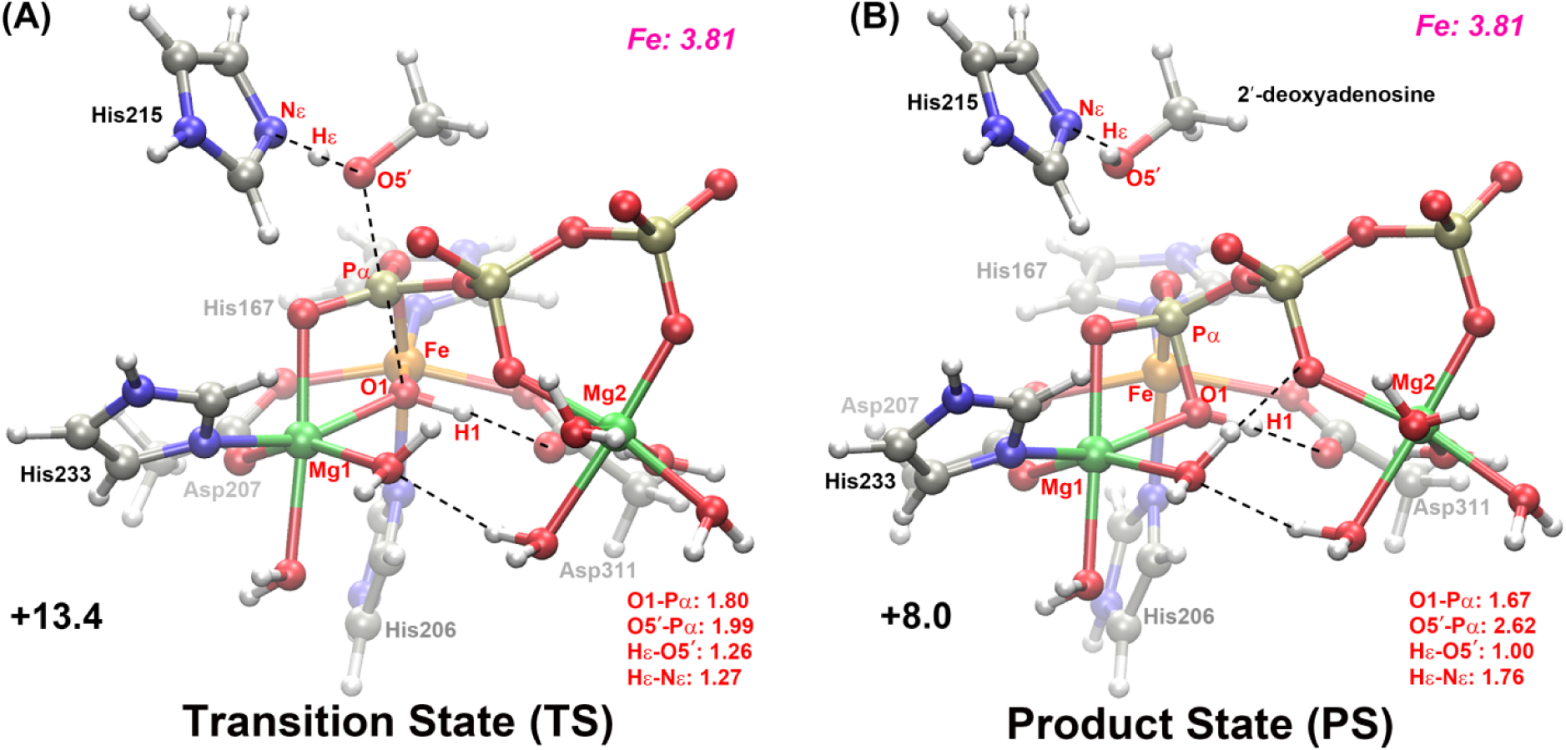
(A) Optimized structure of the transition state at the B3LYP/def2-SVP:MM level. (B) Optimized structure of the product state at the B3LYP/def2-SVP:MM level. The Mulliken spin density on Fe is shown in magenta italics in the upper right corner. All distances are given in Å and are shown in the lower right corner. Energies relative to **RS** are given in kcal/mol.

**Figure 4:**
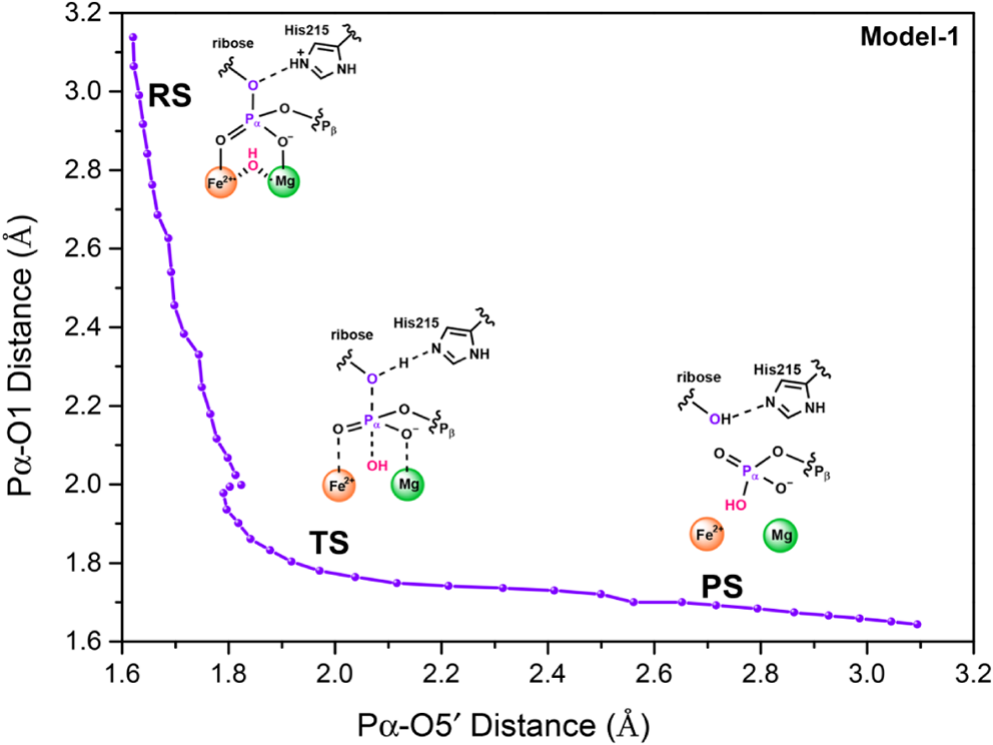
Hydrolysis reaction pathway projected onto the distances of the Pα–O5′ bond (*x*-axis) and the Pα–O1 bond (*y*-axis). Distances are collected from the obtained structures during PES scanning for Model-1.

The calculated activation energy for this S_N_2-type nucleophilic substitution process in SAMHD1 was determined to be 13.4 kcal/mol. A *k*_cat_ value of 0.4 s^–1^ has been measured for the hydrolysis of dATP by SAMHD1.^8^ This value corresponds to a barrier of about 18 kcal/mol, which is slightly higher than the barrier calculated here. This relatively low energy barrier in this study is expected as the reaction is driven by a strong nucleophilic character of the hydroxide ion (OH^–^) that initiates dATP hydrolysis in our computational model. Importantly, the binding process of dATP and the release of the products will also have a significant influence on the SAMHD1’s catalytic efficiency. While these are not investigated in our current study, future work would be necessary to explore substrate delivery and binding, as well as product release for a complete understanding of the catalytic turnover.

After **TS**, Pα gradually regenerates its new optimal tetrahedral geometry, and finally Hε shifts into O5′ when the Pα–O5′ distance increases to around 2.6 Å (see Figure 3B). The product state (**PS**) containing the obtained 2′-deoxyadenosine and inorganic triphosphates in this hydrolysis reaction has an energy of 8.0 kcal/mol relative to **RS**. It is worth noting that various classic mechanisms for phosphate hydrolysis have been well-established and extensively studied through experimental and computational approaches (see Figure S14).^69^ The key differences among these mechanisms primarily stem from the behavior of protons in hydroxide anions and protonated histidines in different enzymes during the corresponding hydrolysis reactions.^19,22,70,71^ In our suggested mechanism of SAMHD1, the transition state involves the cleavage/formation of four key chemical bonds, and a stable intermediate which usually accommodates a negatively charged leaving group in many phosphate hydrolysis enzymes^21,69^ (see Figure S14) that does not exist during the hydrolysis reaction catalyzed by SAMHD1. The protonated histidine His215 donates its proton to the leaving group, accompanied by the cleavage of the Pα–O5′ bond. Besides, the proton H1 in the hydroxide anion does not transfer to the adjacent Asp311, which was previously regarded to grab a proton from the bridging hydroxide^8^ to strengthen the nucleophilic capability of O1 and contribute to the stability of the product state.^22^

In SAMHD1, the bridging hydroxide between Fe and Mg centers is proposed to be a potent nucleophile that attacks the Pα site of dNTP. This dimetal-mediated in-line S_N_2 attack mechanism can be extended to other HD domain phosphodiesterases and triphosphatases (see Figure S4),^17,72^ where two metal ions are bound in a dinuclear configuration by the conserved HD motif. In contrast, the HD domain monophosphatases OxsA^28^ and YfbR^29^ coordinate only a single metal ion via their HD motifs. Notably, a key histidine residue, His233 in SAMHD1, which stabilizes the Mg^2+^ ion at site B, is absent in both OxsA and YfbR (see Figures S5 and S6). These differences may lead to a distinct metal-mediated hydrolytic mechanism employed by mononuclear HD domain monophosphatases.

### 3.2 Fe^3+^ Substitution Abolish the Catalytic Activity

Although the QM/MM calculation results demonstrate that a ferrous ion in the bimetallic center of SAMHD1 is sufficient to catalyze dATP hydrolysis, the possibility that the iron exists in its ferric state cannot be entirely ruled out as in the binuclear metallohydrolase rkbPAP, Fe^3+^ is used to catalyze the hydrolysis of phosphomonoesters with the help of a bridging hydroxide (see Figure S7).^19,41,73,74^ The 100 ns production MD trajectory of the Fe^3+^–Mg^2+^ system reveals that the bridging hydroxide group remains coordinated with both Mg^2+^ and Fe^3+^. Additionally, the presence of Fe^3+^ does not significantly alter the coordination environment compared to the Fe^2+^–Mg^2+^ system, and no other water molecules enter or leave the coordination sphere. To assess the catalytic efficiency of Fe^3+^ in SAMHD1, we subsequently performed energy minimization and potential energy scanning corresponding to the catalytic reaction with an Fe^3+^ ion substituting for the Fe^2+^ at site A in Model-1. A new starting molecular model of the Fe^3+^–Mg^2+^ system, Model-2 (see Figure 5A), was generated on the basis of the optimized structure with Fe^2+^ at site A and Mg^2+^ at site B. Then, we carried out additional energy minimizations and performed PES scanning along the previously used reaction coordinate to determine the energy surface associated with this Fe^3+^-involved reaction. The QM/MM calculations show that the ground state of Model-2 is a sextet with high-spin Fe^3+^, which strongly resembles Model-1 (see Tables S2 and S3). Inspection of the PES scanning results revealed a continuous increase in the energy evaluated at the B3LYP/def2-TZVP:MM level (orange line; see Figure 6A). Any attempts to locate a concerted or stepwise transition state for this reaction were unsuccessful. Nevertheless, to gauge the energy of a possible product in the Fe^3+^-substituted model, we constrained the Pα–O1 bond distance to 1.67 Å based on the product state in Model-1 and found it to be as much as 39.1 kcal/mol higher than the reactant state containing Fe^3+^ and Mg^2+^ (Figure S15). These results suggest that Fe^3+^ is unable to facilitate dATP hydrolysis by SAMHD1.

**Figure 5:**
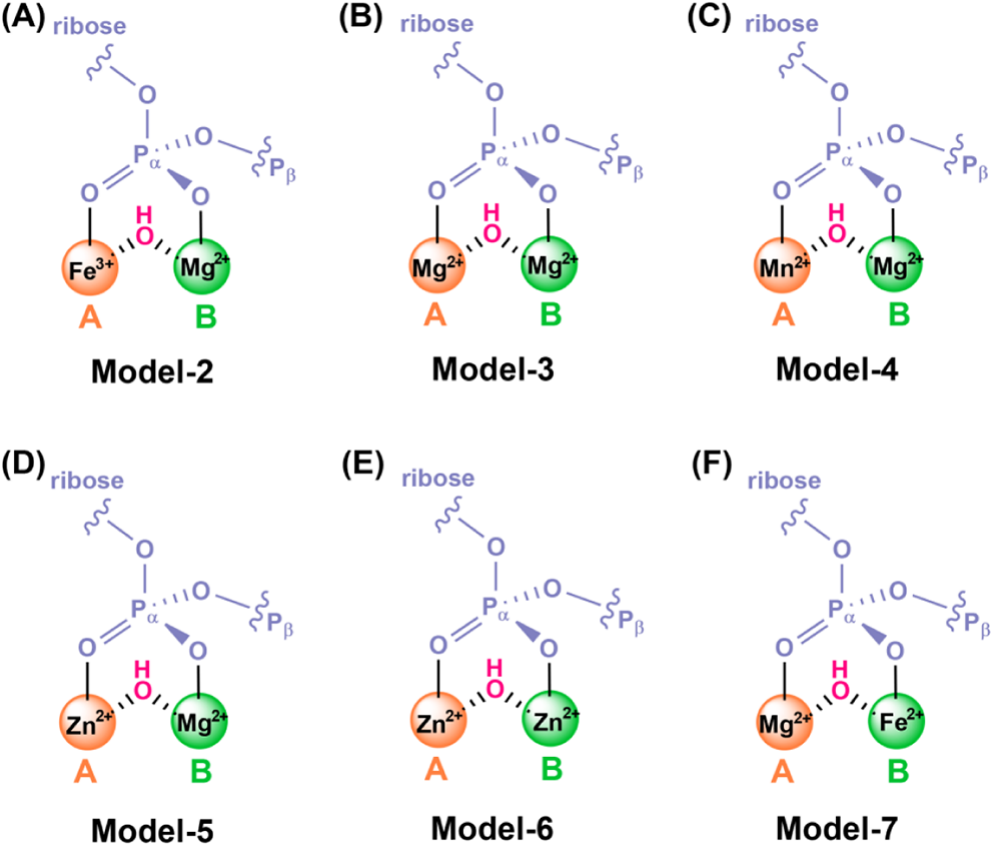
Six constructed models based on the optimized structures of the Fe^2+^–Mg^2+^ system Model-1. In (A) Model-2, (B) Model-3, and (C) Model-4, Fe^2+^ at site A is substituted by Fe^3+^, Mg^2+^, and Mn^2+^, respectively. In (D) Model-5 and (E) Model-6, Fe^2+^ is substituted by Zn^2+^, and Fe^2+^ as well as Mg^2+^ is substituted by Zn^2+^, respectively. (F) In Model-7, Fe^2+^ and Mg^2+^ swap their positions based on Model-1 (see Figure 2B).

**Figure 6:**
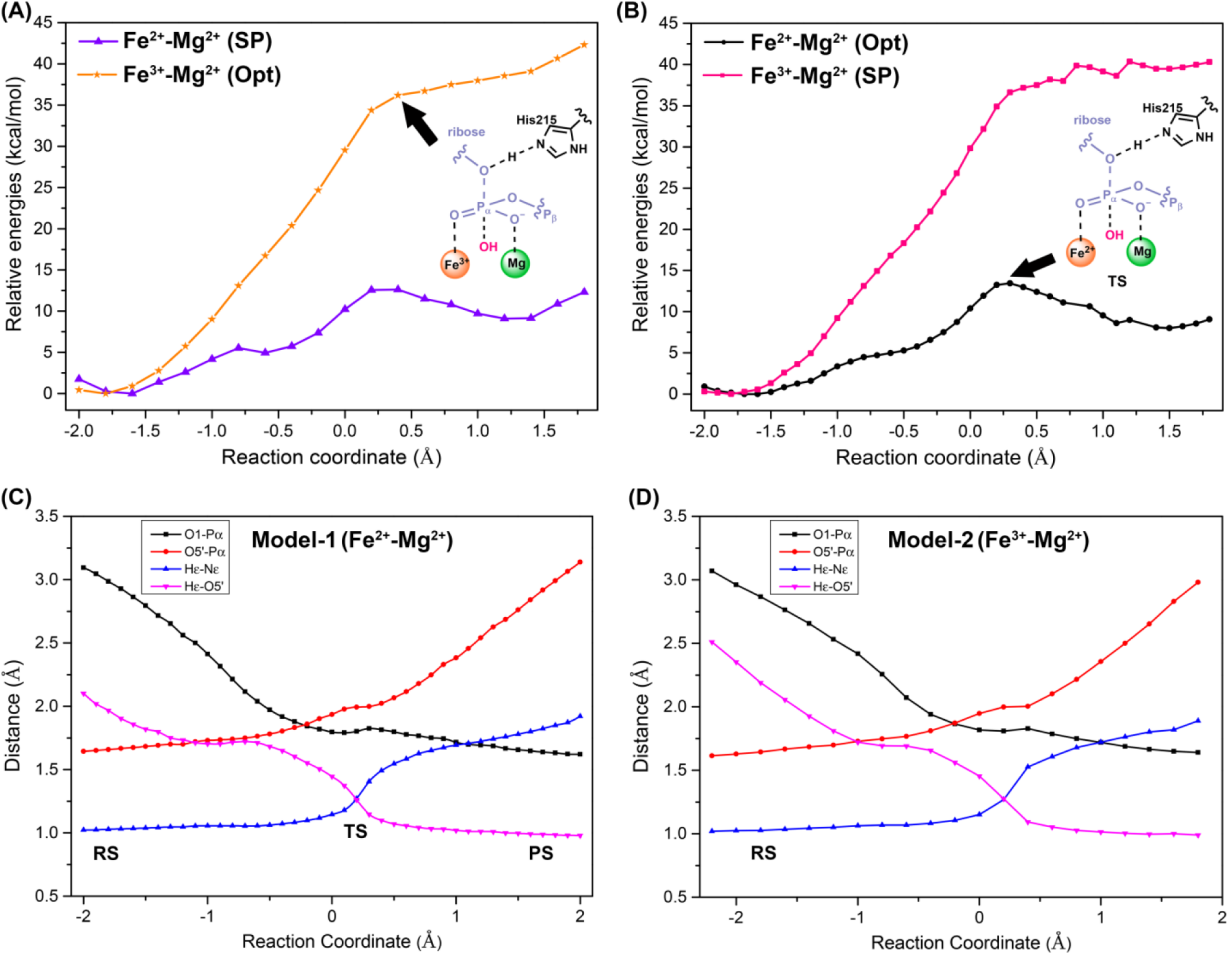
Energy profiles for Fe^3+^ substitutions in SAMHD1. (A) Energy-minimized pathway for the Fe^3+^–Mg^2+^ system (origin, stars, labeled “Opt”). Single-point QM/MM energies at the same level of theory are also shown for Fe^2+^–Mg^2+^ (purple, triangles, labeled “SP”) obtained by replacing Fe^3+^ with Fe^2+^ in all the energy-minimized Fe^3+^-–Mg structures along the reaction. (B) Energy minimized pathway for the Fe^2+^-Mg^2+^ system (black, circles, labeled “Opt”). Single-point QM/MM energies at the same level of theory are also shown for Fe^3+^–Mg^2+^ (pink, square, labeled “SP”) obtained by replacing Fe^2+^ with Fe^3+^ in all the energy-minimized Fe^2+^–Mg^2+^ structures along the reaction. (C) Distances of key chemical bonds along the reaction path using Model-1. (D) Distances of key chemical bonds along the reaction path using Model-2.

The observed catalytic inactivity in the SAMHD1 Fe^3+^–Mg^2+^ simulation system is totally different from the enzymatic behavior characteristic of rkbPAP,^73^ which employs Fe and Zn to construct a similar active site and invokes a bridging hydroxide to attack the phosphorus atom of phosphate (see Figure S7). Fe^3+^ was confirmed to be the form of Fe in rkbPAP by using electron paramagnetic resonance (EPR) measurements^75^ and quantum chemical calculations.^19^ However, the proposed S_N_2 reaction mechanism of rkbPAP is stepwise and different from the mechanism discussed above for SAMHD1 (see Figure S14B): when the bridging hydroxide attacks the phosphorus atom of methylphosphate, P–OCH_3_ bond of the substrate stretches and a stable trigonal-bipyramidal intermediate is formed and then a proton transfers to a leaving group CH_3_O^–^ with a barrier of ∼28 kcal/mol.^19^ By comparing the active sites of SAMHD1 and rkbPAP, we found that Fe^3+^ in rkbPAP is coordinated with a nitrogen ligand and five oxygen ligands, which include a deprotonated tyrosine, and the Fe^3+^ site in rkbPAP is very similar to the Mg1 site in SAMHD1 (see Figures 2 and S7), while Zn^2+^ is coordinated with two nitrogen ligands.^76^ Thus, the divergence in metal-binding residues between SAMHD1 and rkbPAP may contribute to the difference in the valences of iron. Given that metal coordination geometry may influence the catalytic mechanism and metal valence employed in the two enzymes, future studies using quantum chemical methods are required to systematically evaluate the catalytic role of Fe^2+^ in rkbPAP.

To quantify the effects of the Fe^3+^-induced changes in the structure of SAMHD1, single-point QM/MM energies at the same level of theory are also obtained for the Fe^2+^–Mg^2+^ model (see Figure 6A, purple line, labeled “SP”) by replacing Fe^3+^ with Fe^2+^ in all the energy-minimized Fe^3+^–Mg^2+^ structures along the reaction pathway without any further relaxation. In Figure 6A, the pathway and geometries correspond to the Fe^3+^–Mg^2+^ optimized pathway, whereas in Figure 6B, the pathway and geometries correspond to the Fe^2+^–Mg^2+^ optimized pathway. In Figure 6A, the energy profile highlighted in purple indicates that Fe^2+^ keeps its catalytic ability, and the calculated barrier of it is around 13 kcal/mol. On the contrary, Fe^3+^ instead eliminates the enzymatic activity of SAMHD1 when using the energy-minimized Fe^2+^–Mg^2+^ structures (see Figure 6B, pink line, labeled “SP”). By comparing the energy barrier in the Fe^2+^–Mg^2+^–SP system and the previously discussed system in Section 3.1, one can find that Fe^3+^ substitution at site A does not dramatically change the mechanistic character and geometries of the reaction (see Figure 6C,D). However, they can abolish the catalytic activity of SAMHD1.

To further explore the origin of the loss of SAMHD1 catalytic activity in the presence of Fe^3+^, natural population analysis (NPA) was conducted on the optimized reactant structures of Model-1 and Model-2 using the NBO program.^77^ As shown in Table S4, the NPA charges on Fe^2+^ and O1 in Model-1 were calculated to be 2.60 and −0.65, respectively, both of which are lower than the corresponding values for Fe^3+^ and O1 in Model-2 (3.01 and −0.52, respectively). This charge redistribution creates stronger electrostatic polarization effects in Model-2, where the elevated positive charge on Fe^3+^ substantially diminishes the electron density at O1, thereby attenuating its nucleophilicity relative to Model-1. A systematic comparative analysis of α-HOMO orbital distributions between Model-1 and Model-2 revealed an electron delocalization from the iron center to hydroxide ligands, which was exclusively observed in the Fe^2+^-containing system (Model-1, see Figure S16). This phenomenon in Model-1 makes the bridging hydroxide maintain a strong nucleophilic character, in contrast to Model-2 where the absence of such delocalization results in a comparatively weaker nucleophilicity of the hydroxide. Additionally, further Mayer bond order^78,79^ analysis revealed a notably higher bond order value of 0.49 for the Fe^3+^–O1 in Model-2 compared to the Fe^2+^–O1 bond (0.28) in Model-1, suggesting that the presence of Fe^3+^ makes the nucleophilic attack on the phosphate group of dATP less favorable compared to Fe^2+^.

In summary, our calculations demonstrate that the oxidation state of iron in SAMHD1 is crucial for the enzymatic reaction. The ferrous form of iron in SAMHD1 is confirmed to be active, while the ferric form is found to abolish the catalytic activity. In future experimental studies, EPR or Mössbauer spectroscopy could be employed to further determine the oxidation state of iron in SAMHD1 under physiological conditions and validate our computational results.

### 3.3 Metal Selectivity in SAMHD1

In the resolved crystal structure of SAMHD1, Fe at site A is coordinated with six ligands, two of which are nitrogen ligands, His167 and His206, while a hard Lewis acid metal, Mg at site B is coordinated by five oxygen ligands and one nitrogen ligand. Despite uncertainties regarding the roles of ligand residues in SAMHD1, Mn has been reported to be able to replace the metal Mg at site B while retaining the catalytic reaction with similar activity.^8^ A recent study proposed that the rise of O_2_ during the Great Oxidation Event induced a fundamental biogeochemical transition whereby Mg replaced Fe in many metalloproteins, constituting an essential evolutionary adaptation to mitigate oxidative damage in emerging aerobic environments.^80^ As observed in other phosphohydrolases, such as L. blandensis dGTPase,^17^ E. coli dGTPase,^72^ and HD domain phosphohydrolase OxsA,^28^ the metal center regions in their active sites are typically constructed using Mg and/or Mn, but not Fe (see Figures S17 and S18), underlying that SAMHD1 is chemically distinct from general phosphohydrolases. Inspired by the construction of catalytic metal sites in L. blandensis dGTPase^17^ and E. coli dGTPase,^81^ we further investigated whether SAMHD1 retains catalytic activity when the native Fe at site A is substituted with Mg or Mn.

We calculated the energy surfaces corresponding to the catalytic reactions in which the native Fe^2+^ at site A in Model-1 was substituted with Mg^2+^ (Mg^2+^-Mg^2+^, see Model-3 in Figure 5B) or Mn^2+^ (Mn^2+^–Mg^2+^, Model-4 in Figure 5C), although no SAMHD1 crystal structure with Mg^2+^ or Mn^2+^ bound at site A has been reported to date. The full catalytic mechanism was remapped for Model-3 and Model-4 on the basis of the pathway of Model-1, which is discussed above. It should be noted that in our QM/MM calculations of Model-4, Mn^2+^ was treated with the sextet ground state (see Table S5). The obtained geometries of the reactant states in Model-3 and Model-4 are highly similar to those in Model-1 by overlaying the optimized structures of the QM region (see Figure S19). The calculated energy barriers for Model-3 and Model-4 are 15.7 and 13.9 kcal/mol, respectively (see Table S5). According to these QM/MM calculations, SAMHD1 remains catalytically active when Fe^2+^ at site A is replaced by Mn^2+^ or Mg^2+^, but the catalytic efficiency follows the trend Fe^2+^ > Mn^2+^ > Mg^2+^. These data are in agreement with previous observations of SAMHD1 metal ion dependency, which demonstrate that Mg^2+^ and Mn^2+^ are capable of stimulating hydrolysis to different degrees depending on the substrate dNTP.^16^ In contrast, Zn^2+^ has been found to inhibit this reaction in the experiments on metal ion dependency,^16^ although Zn^2+^ is most commonly used in metallohydrolases.^82–84^ It should be stressed that a minimally stable zinc coordination sphere is usually made up of four protein ligands and adopts a slightly distorted tetrahedral or trigonal bipyramidal coordination geometry,^84–86^ but in SAMHD1 the used metals in the active and allosteric sites are octahedrally coordinated. We further computed the potential energy surfaces for Model-5 where native Fe^2+^ was replaced by Zn^2+^ (see Figure 5D), revealing a lower activation barrier of 13.3 kcal/mol. Moreover, a doubly Zn^2+^-substituted Model-6 was also simulated in this study, and the barrier was calculated to be 14.7 kcal/mol (see Table S5). Although the calculated barriers in Model-5 and Model-6 do not confirm Zn’s inhibitory role in SAMHD1, this unique metal selectivity is presumably associated with the coordination geometry. As the metal-binding geometries in both the active and allosteric sites of SAMHD1 are octahedral^8^ (see Figure S2) and theoretically not favorable for Zn^2+^ coordination,^86^ Zn may influence substrate binding or allosteric site formation to reduce the rate of dATP hydrolysis. Further studies are needed to clarify the exact mechanism of zinc inhibition in SAMHD1.

Furthermore, we explored the metal affinity of SAMHD1 using quantum chemical (QC) and QM/MM calculations. In the QC calculations, three metal–water complexes, Fe^2+^(H_2_O)_6_, Mg^2+^(H_2_O)_6_, Mn^2+^(H_2_O)_6_, were optimized using Q-Chem program^62,63^ at the level of B3LYP/def2-SVP, and then the energies of the three optimized geometries were re-evaluated by performing single-point calculations at the level of wB97M-V/def2-TZVP. By comparing the calculated QC and QM/MM energies of Model-1 and Model-4 (see Figures S20 and S21), we found that replacing Fe^2+^ with Mn^2+^ for **RS** was an endothermic process with an energy of 3.9 kcal/mol. This result aligns with experimental observations indicating that the Fe ion at site A is resistant to substitution by Mn.^8^ Similarly, in Mg^2+^–Mg^2+^ system, the hard Lewis acid Mg^2+^ at site A is coordinated with two histidines, leading to an endothermic substitution of Fe^2+^ by Mg^2+^ for **RS**, which is in agreement with the features in other magnesium-dependent proteins,^87^ where magnesium exhibits a stronger preference for coordination with hard Lewis base ligands,^33,34^ such as water molecules and carbonyl oxygen.^80,88,89^ These comparisons highlight the high affinity of SAMHD1 for Fe^2+^ at site A relative to Mn^2+^ and Mg^2+^.

Overall, since Fe^2+^ is a relatively soft Lewis acid while Mg^2+^ is hard,^33,34^ the replacement of Fe^2+^ at site A by Mg^2+^ in the SAMHD1 active site is an endothermic process, and the Mg^2+^–Mg^2+^ bimetallic center slightly increases the energy barrier of dATP hydrolysis according to our QM/MM calculations. Although Mn^2+^ is slightly harder than Fe^3+^ and still supports dATP hydrolysis, the calculation results reveal that the binding energy of Fe^2+^ for site A in SAMHD1 (see Figure S21) is larger than Mn^2+^. Thus, SAMHD1 exemplifies a conserved evolutionary paradigm where contemporary biochemical systems retain the latent capacity to reactivate ancestral Fe^2+^-dependent metabolic states under pre-Great Oxidation Event conditions.^80,90^

### 3.4 Site Selectivity in SAMHD1

Fe has rarely been found to catalyze the enzymatic reaction in the HD domain superfamily, except for diiron-dependent oxygenase.^9,10^ While several oxygenases in this family, such as mammalian myoinositol oxygenase (MIOX),^24^ bacterial phosphonate oxygenase PhnZ,^23^ and nonheme iron oxygenase TmpB^91^ strictly coordinate dual iron ions to construct bimetallic centers and bind dioxygen for their catalytic function, SAMHD1 instead demonstrates an evolutionarily distinct metallocofactor strategy to bind dNTP, utilizing a single Fe^2+^ at site A as well as Mg^2+^ at site B.^15^ This distinction suggests that magnesium plays a critical role in defining the active site architecture of SAMHD1 (see Figure 1) and exhibits a stronger inclination than iron to occupy site B. To investigate why Fe^2+^ preferentially occupies site A rather than site B, we performed a metal position exchange based on their respective native locations in the experimentally resolved crystal structure (PDB code 6TX0)^8^ and further carried out QM/MM calculations for the exchanged system.

Based on the **RS** structure shown in Figure 2, a new model, Model-7, was constructed, in which Fe^2+^ occupies site B while Mg^2+^ is positioned at site A (see Figure 5F). To assess the geometric effects during this exchange, single-point calculations were performed on a nonoptimized reactant state structure, where all atomic coordinates remained identical to those in the **RS** of Model-1, except for the swapped Fe^2+^ and Mg^2+^ (see Figure S22). The calculations show that the energy of this nonoptimized reactant state structure is 35.0 kcal/mol higher than **RS**. Then, energy minimizations for this nonoptimized reactant state of Model-7 were done to obtain **RS-M7,** an optimized reactant state of Model-7 (see Figure S23A). Similarly, based on the structure of **RS-M7**, single-point calculations were performed on a new nonoptimized **RS** structure, where all atomic coordinates remained identical to those in **RS-M7** of Model-7, except for the reswapped Fe^2+^ and Mg^2+^ (see Figure S23B). And this nonoptimized **RS** structure has an energy of −28.7 kcal/mol relative to the **RS-M7**, indicating that Model-1 is more favorable than Model-7 for the coordination mode in the active site of SAMHD1.

## 4 Conclusions

In this study, we employed molecular dynamics (MD) simulations and QM/MM calculations to investigate the mechanistic details of dNTP hydrolysis mediated by collaborative two-metal catalysis and elucidate the specific role of iron in human SAMHD1. This triphosphohydrolase, an exceptional member of the HD domain family, plays a critical role in regulating dNTP pools in inhibiting the replication of HIV-1 and other retroviruses.

To examine the role of iron, one of the two metals employed by SAMHD1 to construct its active site, we investigated the reaction mechanism using dATP as a substrate and probed the effect of single ion substitutions in SAMHD1 through QM/MM simulations. Our calculations demonstrate that Fe^2+^ in SAMHD1 assists in catalyzing the dATP hydrolysis via an unusual concerted P–O bond cleavage and proton transfer pathway with a barrier of 13.4 kcal/mol. Single-ion substitution at site A from Fe^2+^ to Fe^3+^ abolishes catalysis in the reaction catalyzed by SAMHD1. Further NPA results reveal that Fe^3+^ substantially diminishes the electron density at the oxygen atom of the nucleophile hydroxide anion, thereby attenuating its nucleophilicity relative to the Fe^2+^–Mg^2+^ system and inhibiting dATP hydrolysis. Additionally, Mg^2+^ and Mn^2+^ are capable of supporting the hydrolysis of dNTP without the assistance of Fe^2+^ but the combined QC and QM/MM calculations for the Mg^2+^-substituted and Mn^2+^-substituted models also reveal that neither Mn^2+^ nor Mg^2+^ is favored over metal site A in the binding pocket when compared with the native metal iron, with the energy required for substitution of Fe^2+^ ranging from 4 to 10 kcal/mol. The calculated sufficiently low energy barriers observed in the simulated Zn^2+^-substituted models imply that zinc may influence SAMHD1 activity by disrupting substrate binding or allosteric site formation rather than inhibiting the hydrolysis reaction itself.

SAMHD1 plays an important role in inhibiting early-stage retroviral infection by depleting the dNTP pool to levels insufficient for viral reverse transcription. However, pathogens have wisely evolved some resistance proteins that effectively counteract the catalytic activity of SAMHD1.^92^ We believe that these insights gained from the present QM/MM calculations will serve as a valuable foundation for modulating SAMHD1 activity and guiding the design of novel drugs for antiviral therapies.

## Supplementary Material



## Notes

Additional computational results, optimized structures, energies for all stationary points, and Cartesian coordinates of QM region from QM/MM calculations (PDF)
