# Supplementary material for: Reaction Mechanism and Metal Selectivity of Human SAMHD1 Elucidated by QM/MM Calculations
Source: ACS Catal. 2025 Jun 1;15(12):10176–87. doi: 10.1021/acscatal.5c01682 (PMC12186264; doi:10.1021/acscatal.5c01682)
Supplement: Supplementary file 1 [file cs5c01682_si_001.pdf]

Supporting Information for

Reaction Mechanism and Metal-Selectivity of Human SAMHD1 Elucidated by QM/MM Calculations

Wen-Hao Deng[a,b], Harry Lewin[b], Rong-Zhen Liao*[,a] and Edina Rosta*[,b]

[a]Key Laboratory of Material Chemistry for Energy Conversion and Storage, Ministry of Education, Hubei Key Laboratory of Bioinorganic Chemistry and Materia Medica, Hubei Key Laboratory of Materials Chemistry and Service Failure, School of Chemistry and Chemical Engineering, Huazhong University of Science and Technology, Wuhan 430074, P. R. China.
[b]Department of Physics and Astronomy, University College London, Gower Street, London WC1E 6BT, United Kingdom.

*Corresponding authors: e.rosta@ucl.ac.uk; rongzhen@hust.edu.cn

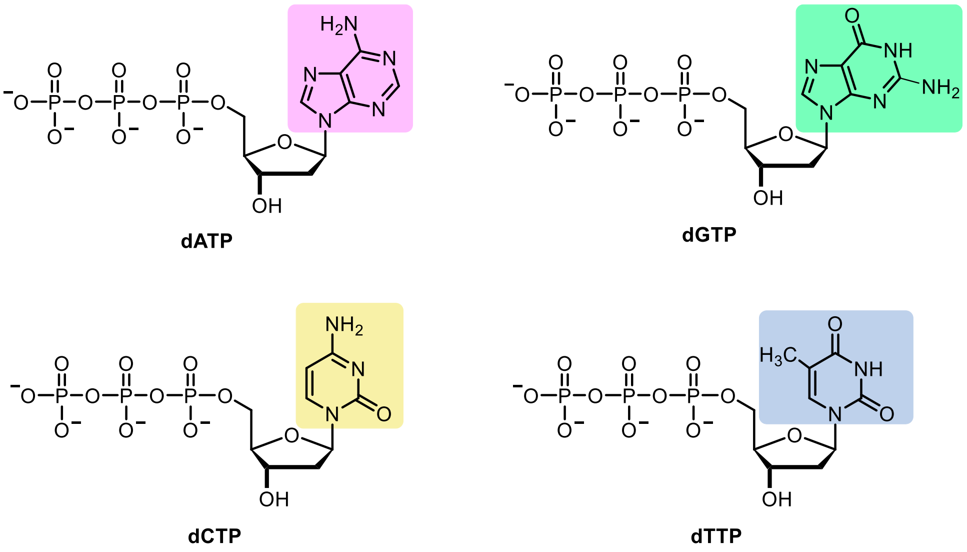

Figure S1. Four types of dNTP: dATP, dGTP, dCTP, and dTTP. The base groups in these molecules are highlighted.

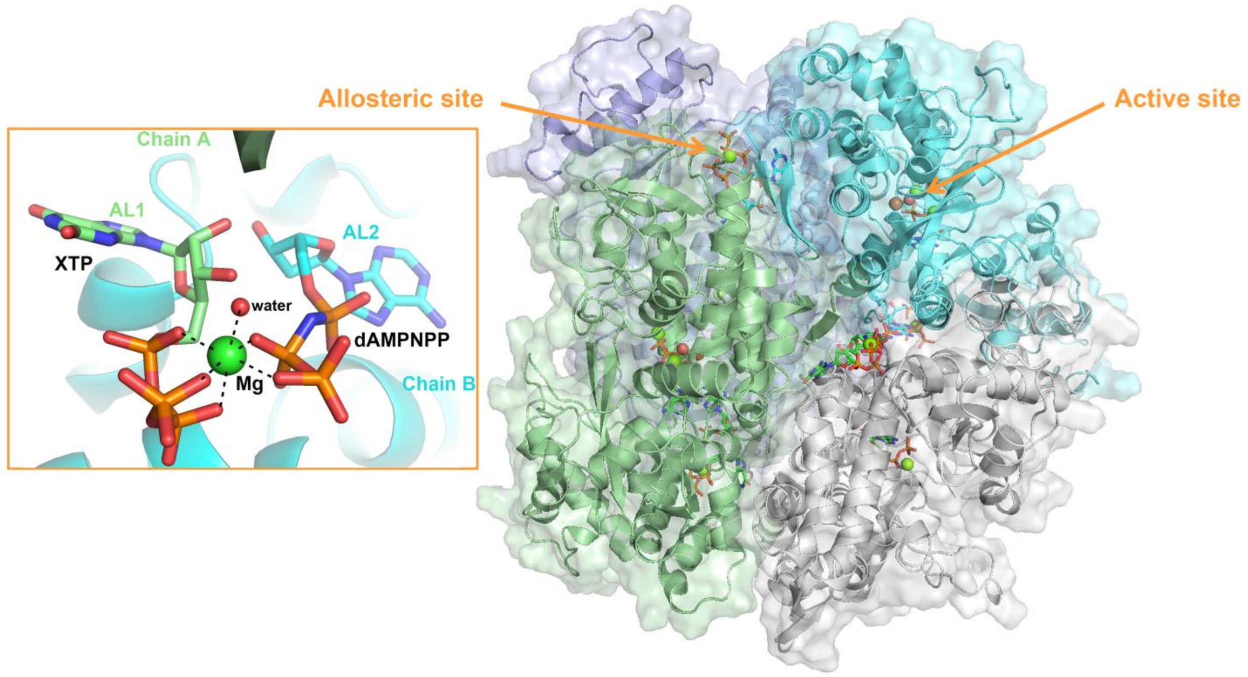

Figure S2. Overview of the D137N-SAMHD1 structure (PDB code 6TX0).[1] It consists of four identical chains, each containing an active site that accommodates the substrate dNTP along with three metal ions. It should be mentioned that this resolved structure is a D137N mutation. Thus, the allosteric sites AL1 and AL2 shown on the left side are located in the interface between each pair of monomers, where xanthosine-5'-triphosphate (XTP) from chain A and 5'-(α,β-imido)triphosphate dAMPNPP from chain B coordinate with a magnesium ion.[1] This structure has a mutation in the allosteric site (D137) so that XTP at AL1 is capable of activating SAMHD1 rather than GTP. The details of the active site are shown in **Figure 1**.

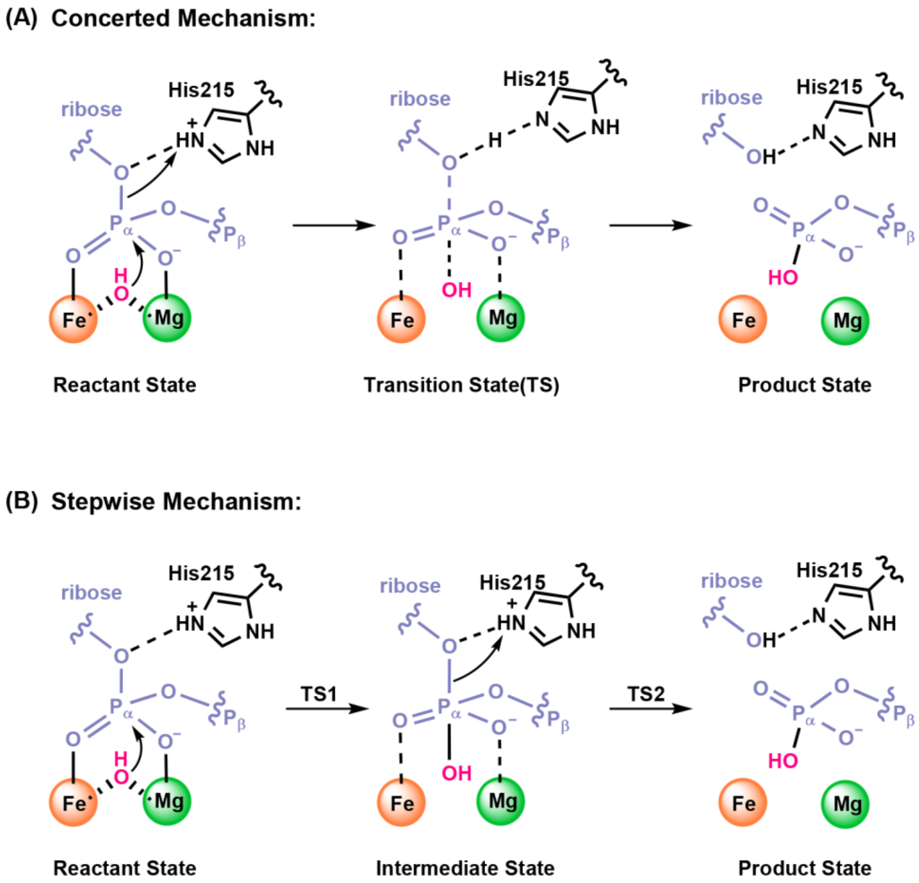

Figure S3. Two possible reaction mechanisms of dNTP hydrolysis by SAMHD1. (A) A concerted mechanism of SAMHD1 initiates with a nucleophilic attack on the Pα atom by the bridging hydroxide anion.[1] (B) A stepwise mechanism involves a nucleophilic attack and proton transfer.

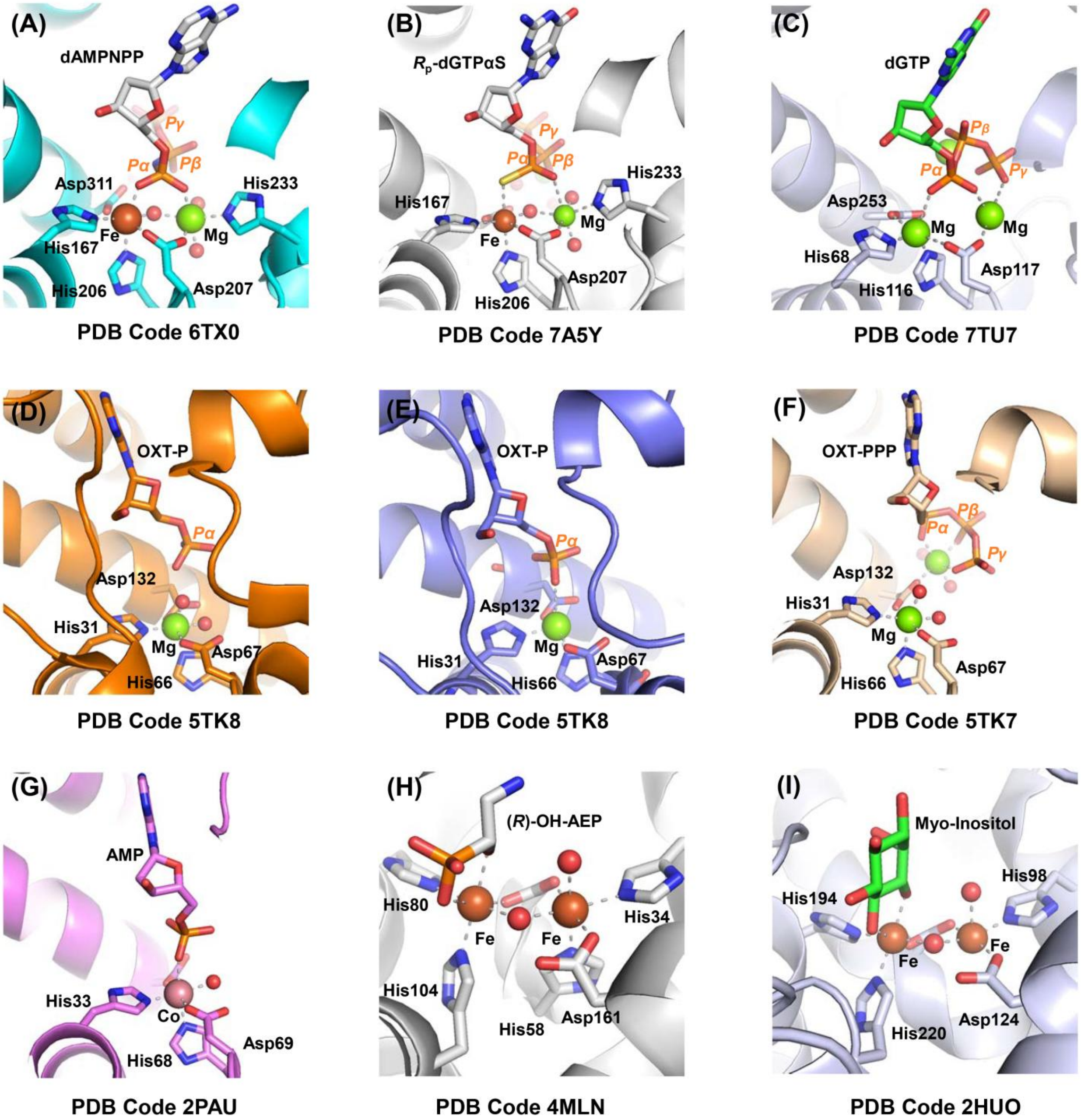

Figure S4. Overview of the active sites of several representative members in the HD domain family. The protein backbone are shown in cartoon representation. Fe and Mg ions are represented as brown and green spheres, respectively. Coordinated waters are shown as red spheres. (A) and (B) SAMHD1's residues that coordinate dAMPNPP (PDB Code 6TX0)[1] and Rp-dGTPαS (PDB Code 7A5Y),[2] respectively. In the active sites, Fe ion is coordinated with six ligands, including a bridging hydroxide group. In SAMHD1, the two oxygen atoms at α phosphate of the substrate coordinate with Mg and Fe ions, respectively. There is no other sites for water moleculars to bind with the Fe center. (C) Active site of dGTPase, which is one of hexameric dNTPase homologs and catalyse the hydrolysis of dGTP (PDB Code 7TU7, see **Figure S16**).[3] (D) Active site of OxsA,[4] which catalyzes the hydrolysis of oxetanocin-5'-monophosphatase (OXT-P, see **Figure S17**). This complex likely represents an inactive enzyme-substrate

complex because two water molecules binds the Mg ion, while OXT-P does not coordinate the Mg ion. (E) A predicted protein complex formed by OxsA.[4] This predicted structure was generated using AlphaFold3.[5] In this active site, only one oxygen atom of OXT-P coordinates with Mg ion. Thus, unlike SAMHD1, a water molecule in OxsA may attack Pα site to trigger the cleavage of the Pα–O bond of OXT-P.[4] (F) Active site of OxsA which binds oxetanocin-5'-triphosphate (OXT-PPP). (G) Active site of 5'-deoxynucleotidase YfbR (PDB Code 2PAU).[6] YfbR is a phosphatase that catalyzes the hydrolysis of AMP to yield 2'-deoxyriboadenosine and inorganic phosphate. (H) Active site of diiron dependent enzyme PhnZ (PDB Code 4MLN).[7] One of the Fe ions is coordinated with a water molecule and a hydroxide ion.[7-9] (I) Active site of diiron dependent enzyme MIOX (PDB Code 2HUO).[10]

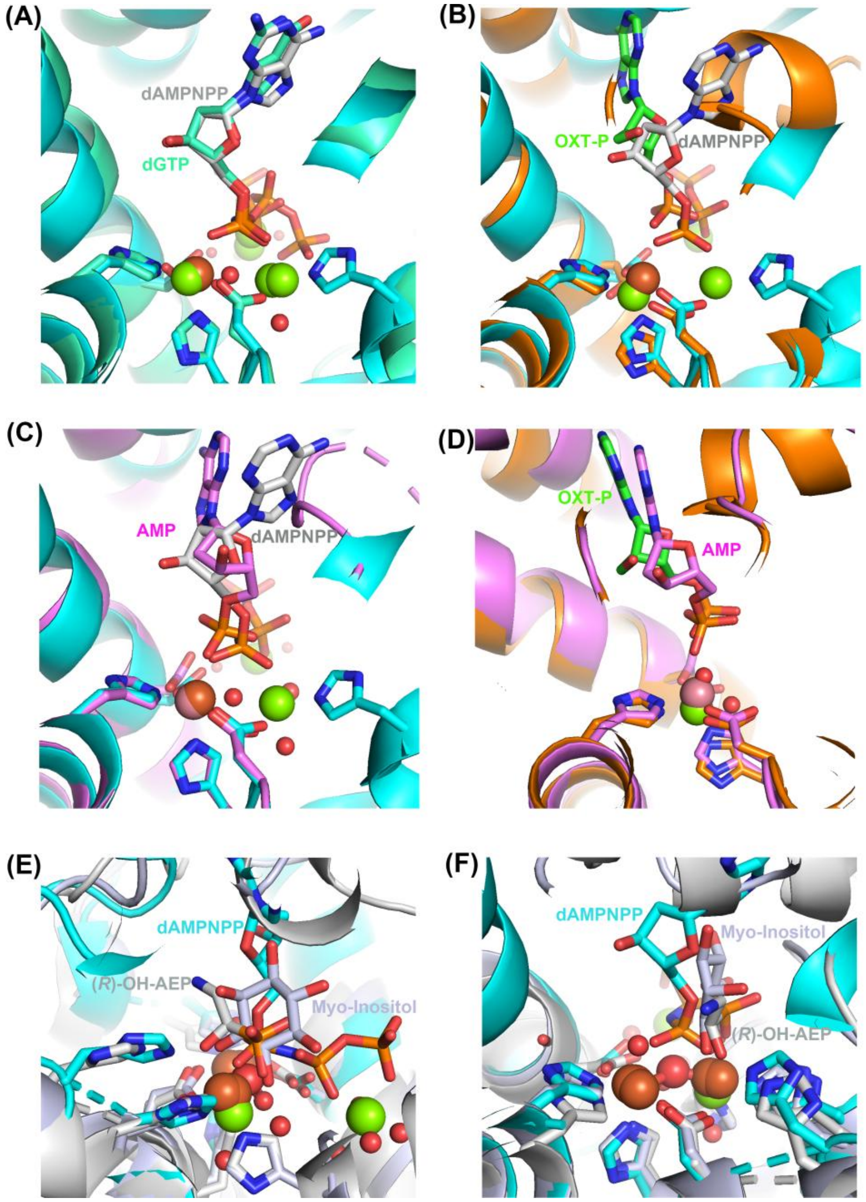

Figure S5. Overlay of the active sites of the representative members in the HD domain family: (A) SAMHD1 (PDB Code 6TX0, in cyan),[1] and dGTPase from *Leeuwenhoekiella blandensis* (PDB Code 7TU7, light green). Both are HD domain triphosphatases. (B) SAMHD1 (cyan) and OxsA (PDB Code 5TK8, brown),[4] (C) SAMHD1 (cyan) and YfbR (PDB Code 2PAU, purple),[6] (D) OxsA (brown), and YfbR (PDB Code 2PAU, fuchsia). Both are HD domain monophosphatases. In the active sites of OxsA, YfbR and dGTPase from *Leeuwenhoekiella blandensis*, a conserved histidine, which binds the second metal ion at site B, is absent. In SAMHD1, this histidine, His233, coordinates the second metal Mg ion. More details of the active sites are shown in **Figure S4**. (E) and (F) SAMHD1 (cyan) and PhnZ (PDB Code 4MLN, grey),[7] and MIOX (PDB Code 2HUO, light purple).[10] The protein backbone are shown in cartoon representation. Fe and Mg ions are represented as brown and green spheres, respectively. Coordinated water molecules are shown as red spheres. It demonstrates that the conserved residues, i.e., the histidines and aspartic acids, coordinate well with the Fe or Mg ions.

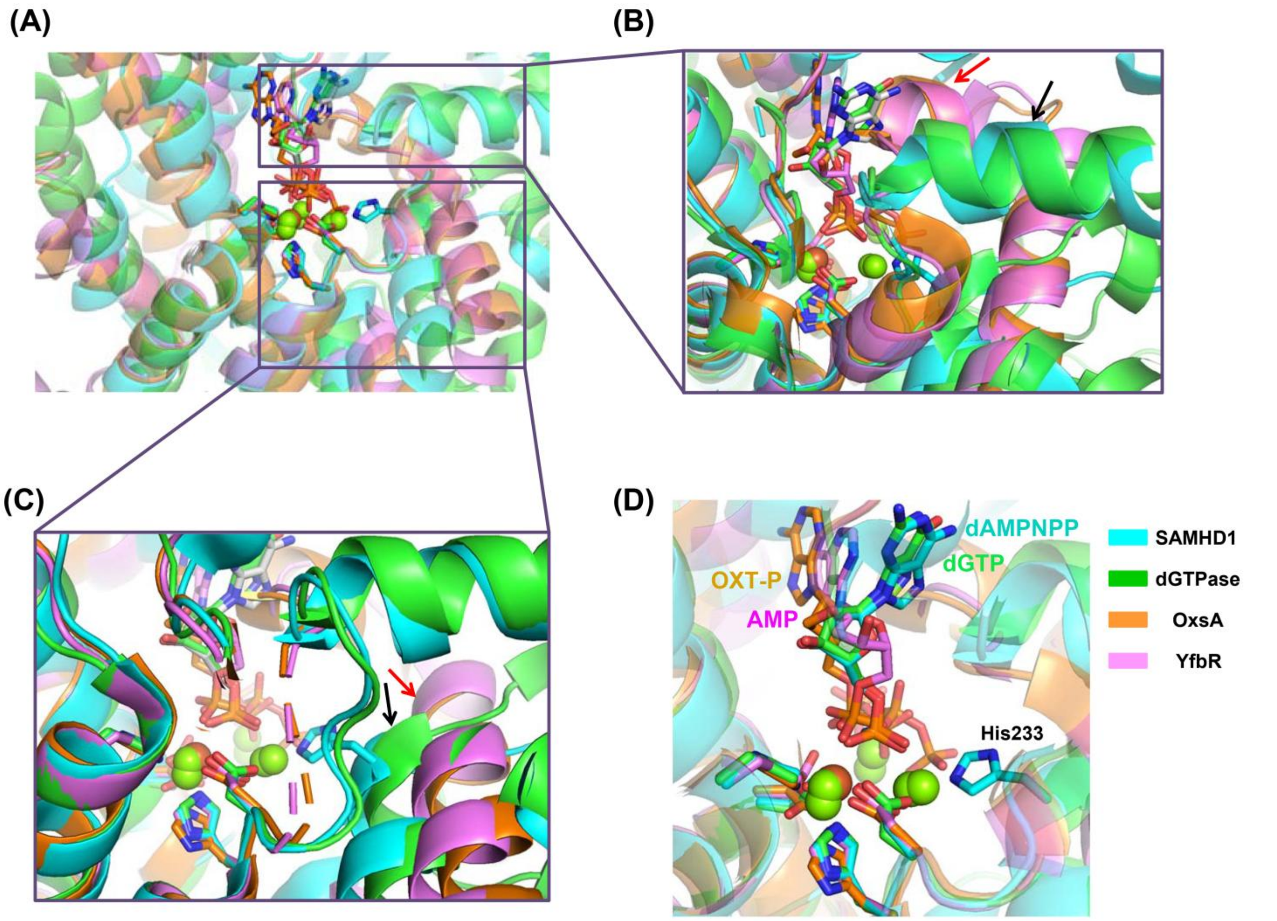

Figure S6. (A) Overlay of the active sites of SAMHD1 (in cyan, PDB Code 6TX0, triphosphatase),[1] dGTPase (in green, PDB Code 7TU7, triphosphatase),[3] OxsA (in brown, PDB Code 5TK8, monophosphatase)[4] and YfbR (in fuchsia, PDB Code 2PAU, monophosphatase).[6] It demonstrates that these four structures are overlayed well based on the conserved residues around their HD motifs, which coordinate with the metal ions. Fe and Mg ions are represented as brown and green spheres, respectively. Regarding the structures, dGTPases share more similarity with SAMHD1 than OxsA or YfbR. (B) and (C) Two short α-helix parts from SAMHD1 and dGTPase are different from that in the two monophosphatases OxsA (in brown) and YfbR (in fuchsia). These distinctions highlighted by arrows result in different binding conformation of the substrate (see **Figures S5A-S5D**). (D) Active sites bound with the substrates in the four enzymes, showing that the 2'-deoxynucleoside group of the dNTP substrate in the HD domain triphosphatases SAMHD1 (in cyan) and dGTPase (in green) adopts a conserved conformation within the binding pocket. More details of the active sites are shown in **Figures S4** and **S5.**

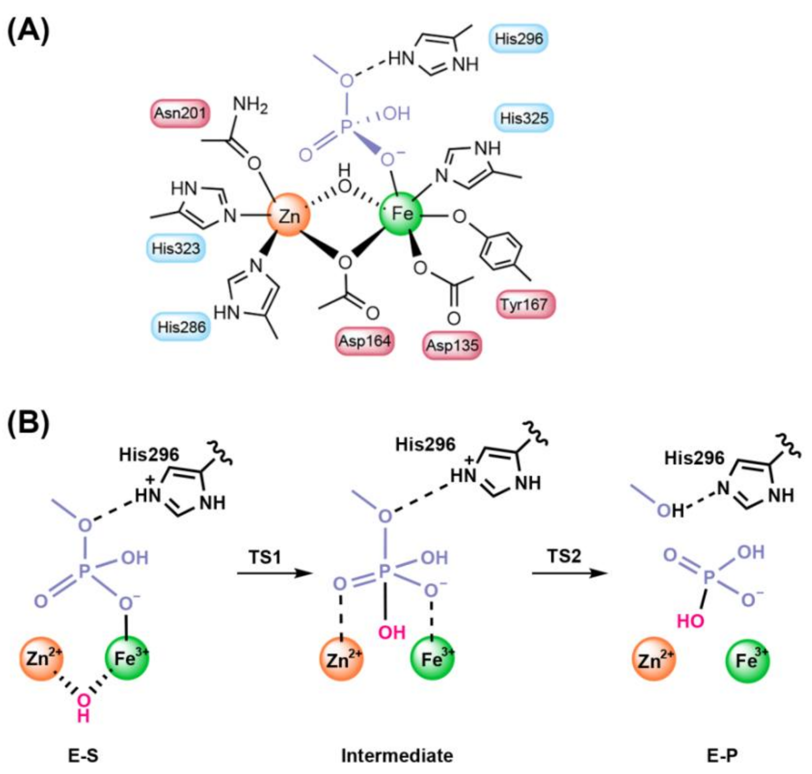

Figure S7. (A) Schematic illustration of the active site of rkbPAP.[11] The substrate is methyl-monophosphate and highlighted in light blue. (B) A proposed hydrolysis mechanism of rkbPAP by Russo et al.[12]

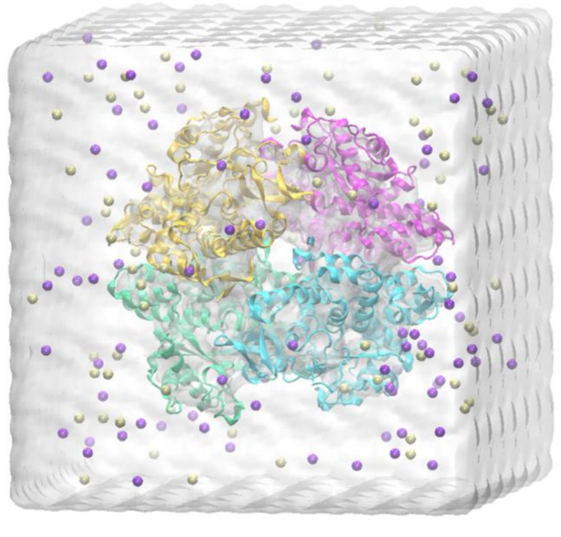

Figure S8. The final model constructed using CHARMM_GUI[13,14] consists of 201597 atoms, including 1944 amino acid residues, 56409 TIP3P water molecules, 73 chloride ions, 89 potassium ions, and the substrate dATP and GTP.

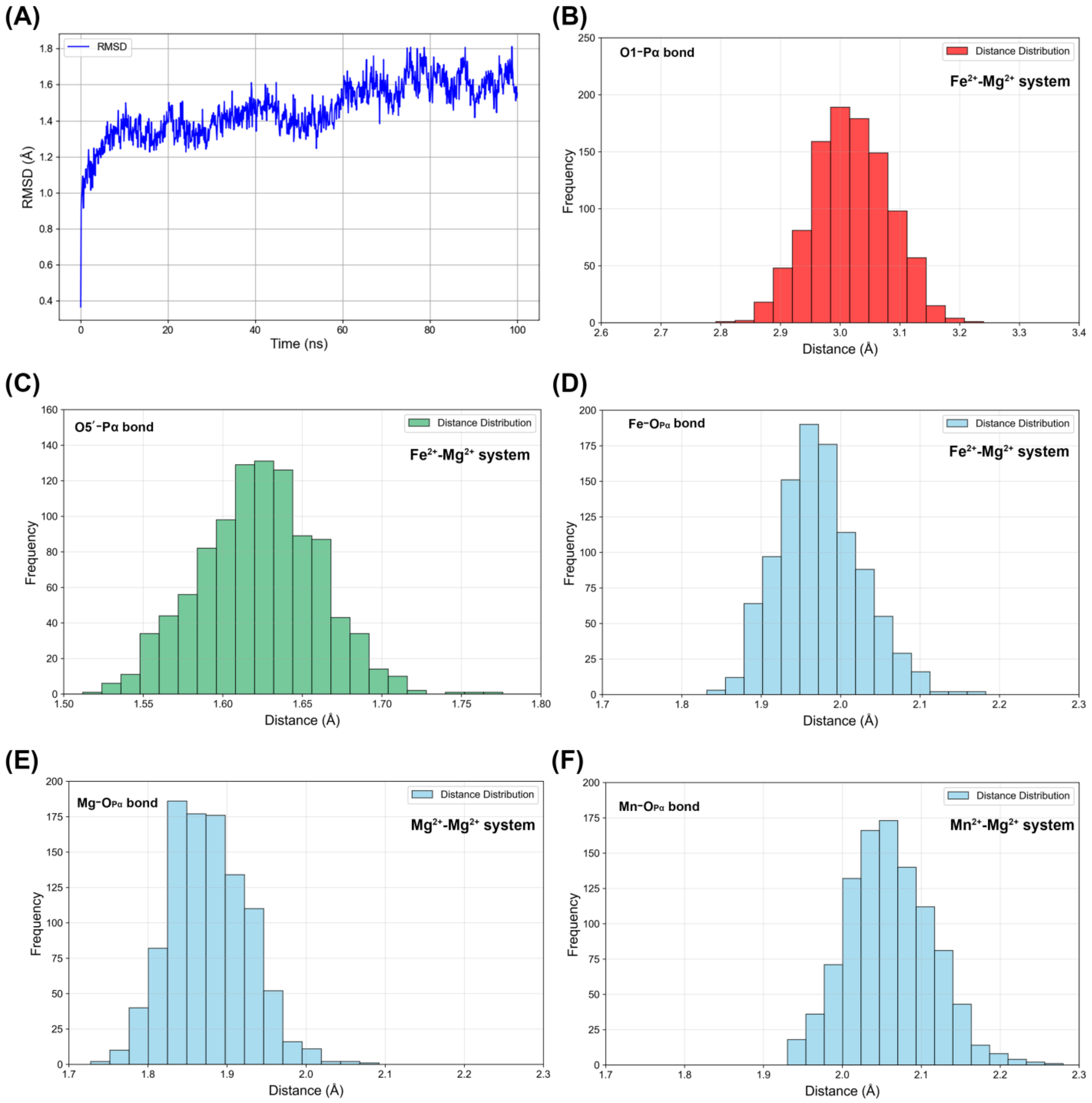

Figure S9. (A) RMSD of backbone atoms for the 100 ns molecular dynamic simulation for SAMHD1 with the substrate dATP molecules, Fe^{2+}, and Mg^{2+} ions. The average of this production runs is 1.461 Å. We also carried out two additional 100 ns production runs using the same equilibrated structure, which was obtained from the 25 ns equilibration with the NPT ensemble. In the two additional production simulations, the calculated average RMSD values are 1.462 Å and 1.443 Å, respectively. (B) Distribution of distances from the O1 of a hydroxide group to Pα of a dATP molecule, which locates in the active site of chain A. 1000 snapshots were selected based on the 100 ns production runs. (C) Distribution of distances of O5'–Pα bond in the dATP molecule. (D), (E), and (F) Three trajectory from different systems, Fe^{2+}-Mg^{2+}, Mg^{2+}-Mg^{2+}, and Mn^{2+}-Mg^{2+}, were compared. Distribution of the distances from the $O_{P\alpha}$ of the dATP molecule to the metal iron at site A (see **Figure 2**) in chain A.

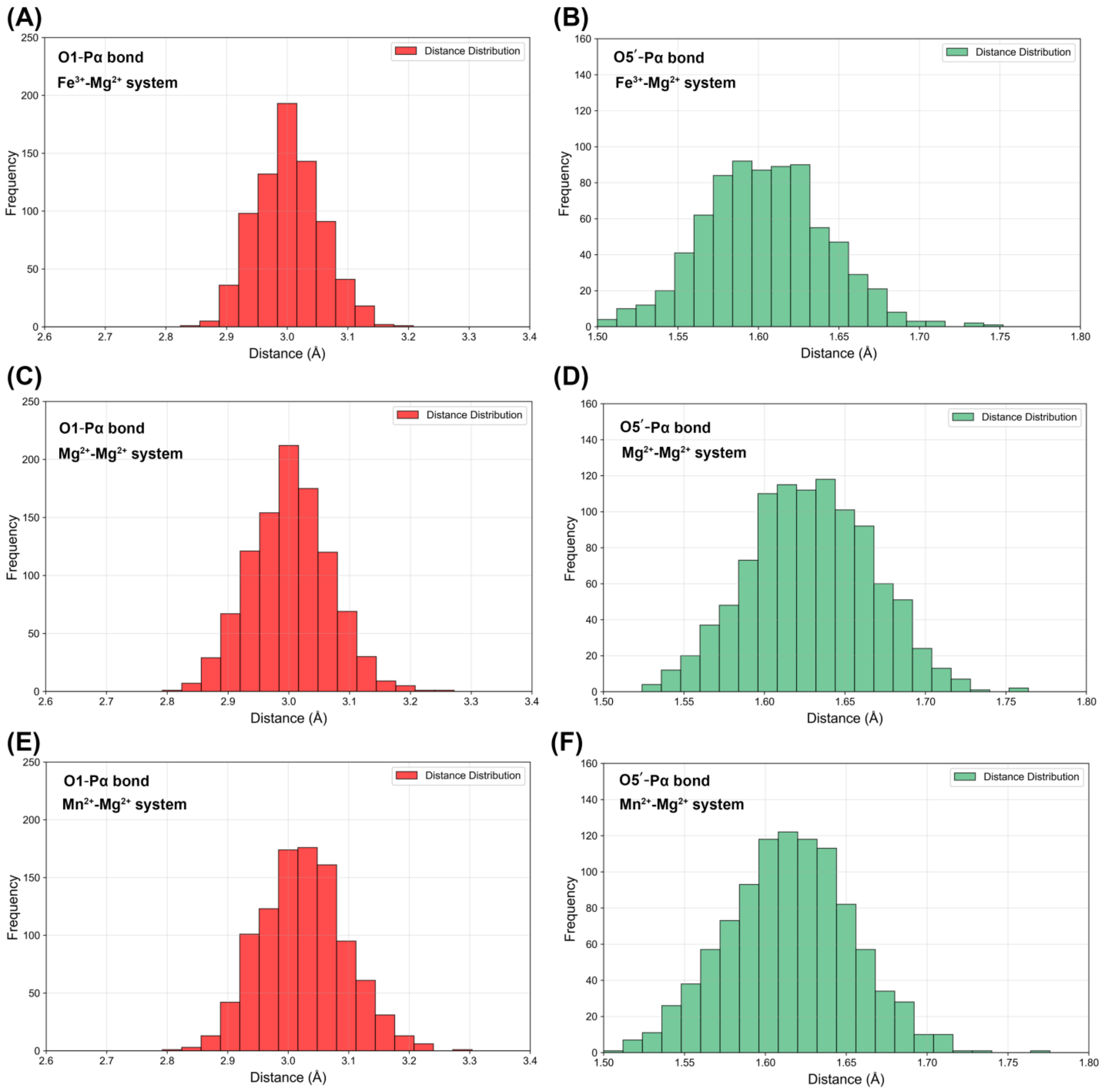

Figure S10. Results of the 100 ns molecular dynamic simulation for the three system, Fe^{3+}-Mg^{2+}, Mg^{2+}-Mg^{2+}, and Mn^{2+}-Mg^{2+}. (A), (C) and (E) Distribution of distances from the O1 of the hydroxide group in chain A to Pα of a dATP molecule, which locates in the active site of chain A. 1000 snapshots were selected based on the 100 ns production runs. Chain A was used to calculate these distances. (B) Distribution of distances of O5'–Pα bond in the dATP molecule.

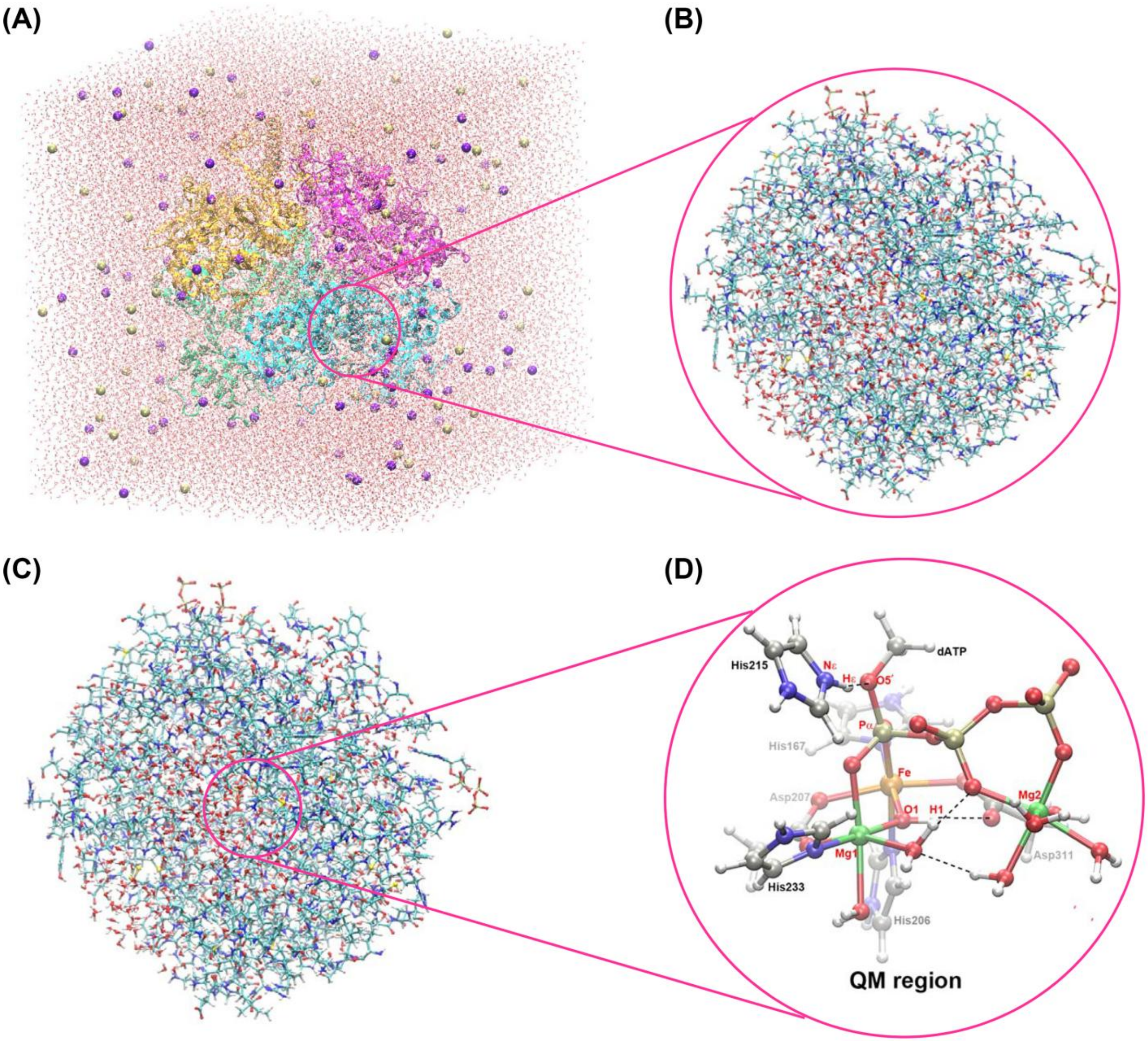

Figure S11. (A) The full equilibrated system. The four chains of SAMHD1 are solvated by a water box. (B) and (C) The full equilibrated system was trimmed to a sphere of 25 Å centred on the ferric iron ion Fe in chain A. This small part consists of 7758 atoms. (D) The structure of the selected QM region for QM/MM calculations.

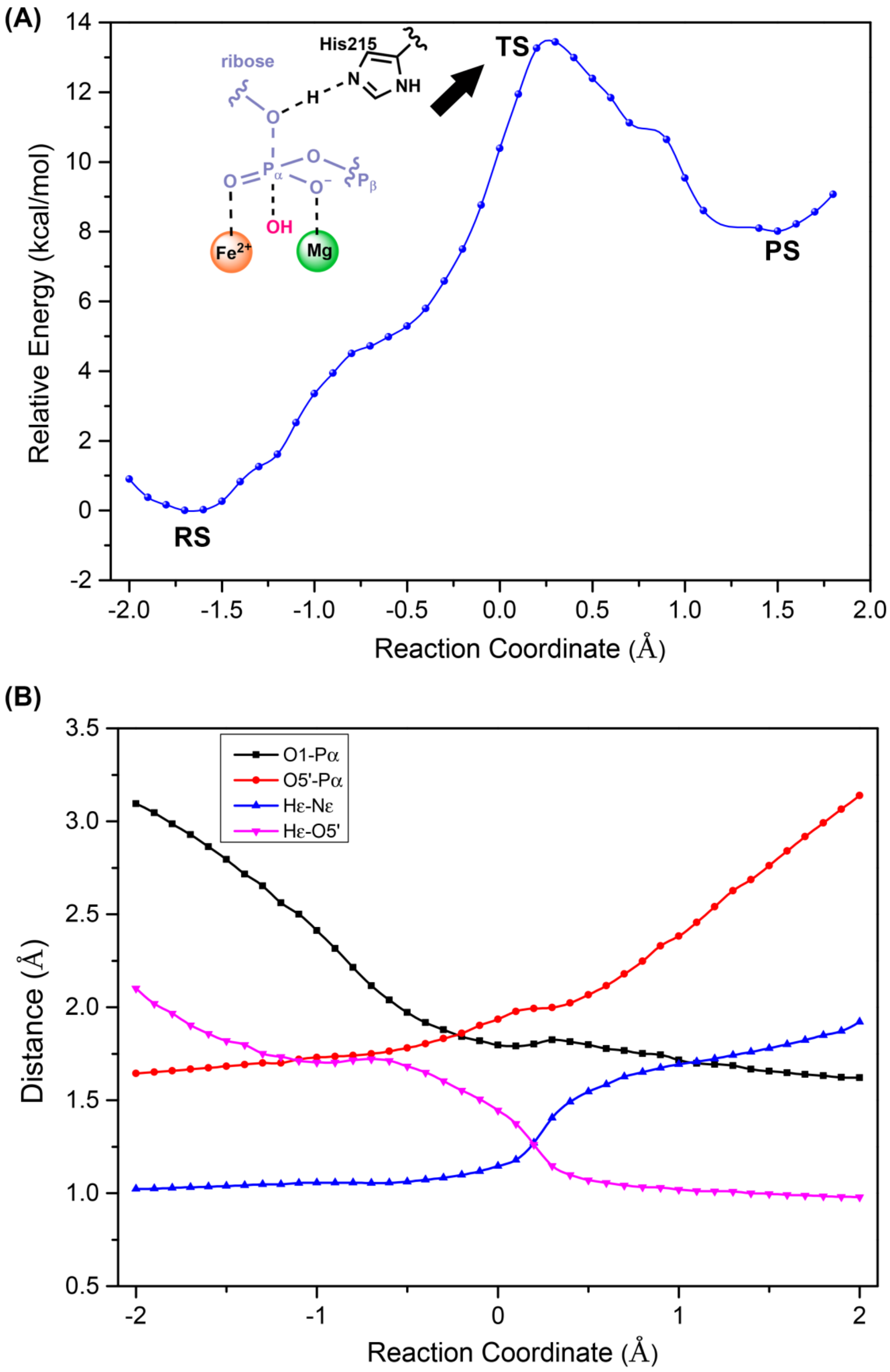

Figure S12. (A) QM/MM minimized paths of hydrolysis of dATP with the help of His215. The reaction coordinate is d(O1–Pα bond) − d(O5'–Pα bond) + 0.5*d(Hε–Nε) − 0.5*d(Hε–O5'). Considering that the transition state in this reaction involves the cleavage/formation of four key chemical bonds: Pα–O1, Pα–O5', Nε–Hε (H215), and Hε–O5', and the importance of the cleavage of Pα–O5' bond of dATP, we used different weights for the P–O bonds cleavage/formation and proton transfer from His215 to dATP based on the previous analogous QM/MM studies.[15-17] (B) Distances of key chemical bonds along the reaction path.

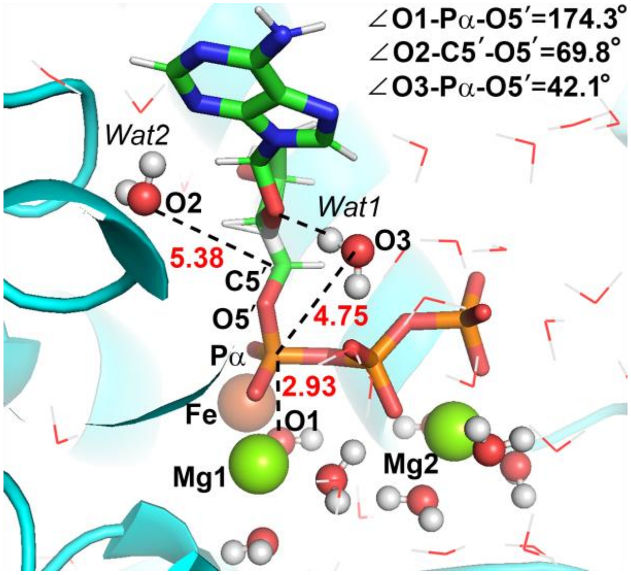

Figure S13. Overview of the active site in the optimized structure of the reactant state. Two water molecules from the MM region are included; however, neither acts as a nucleophile to attack C5' or Pα to initiate the reaction. Critical bond distances are indicated in red, and bond angles (in degrees) are shown at the upper right corner. The critical distances (in Å, red) of bonds are represented by dashed lines.

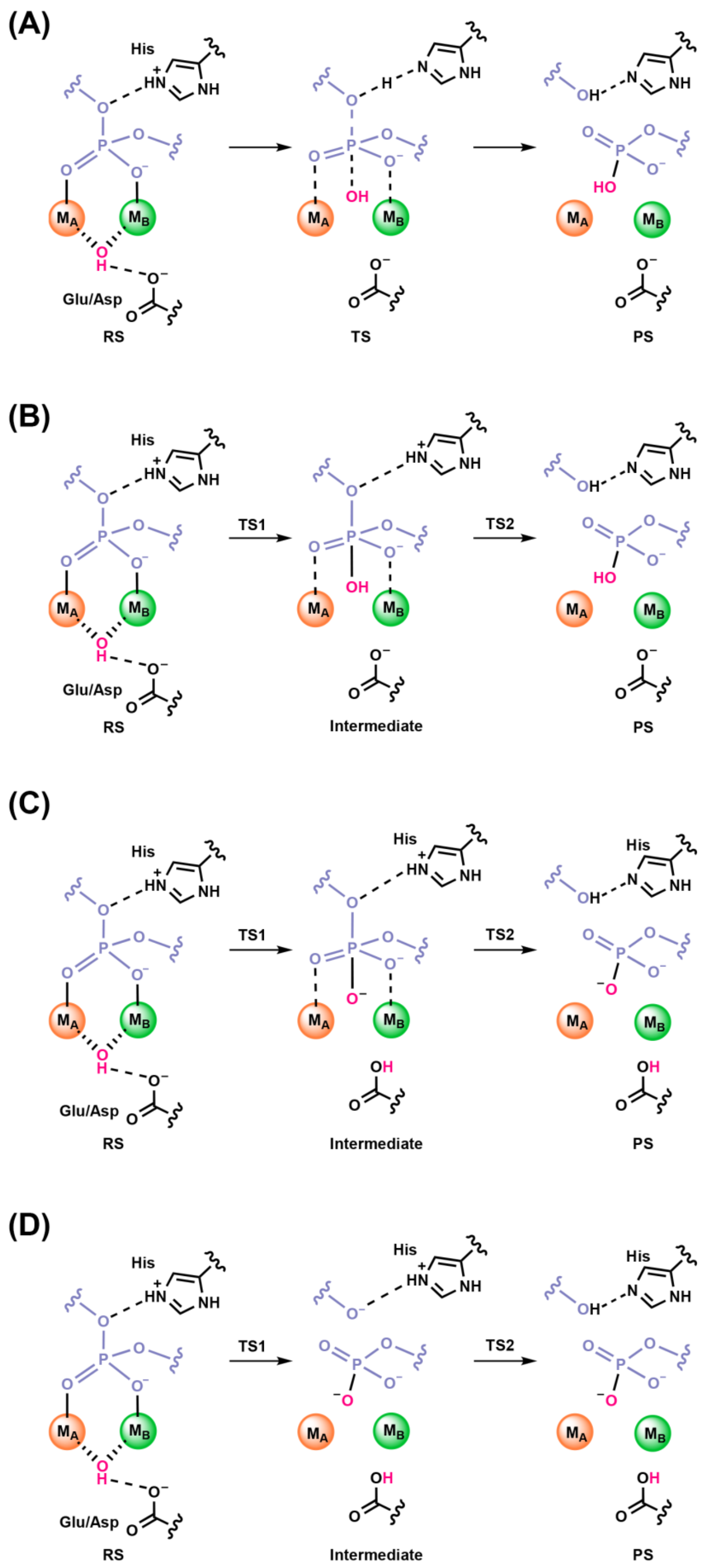

Figure S14. Previously proposed mechanisms of phosphates hydrolysis by bi-metal-dependent phosphohydrolases.[12,18-22]

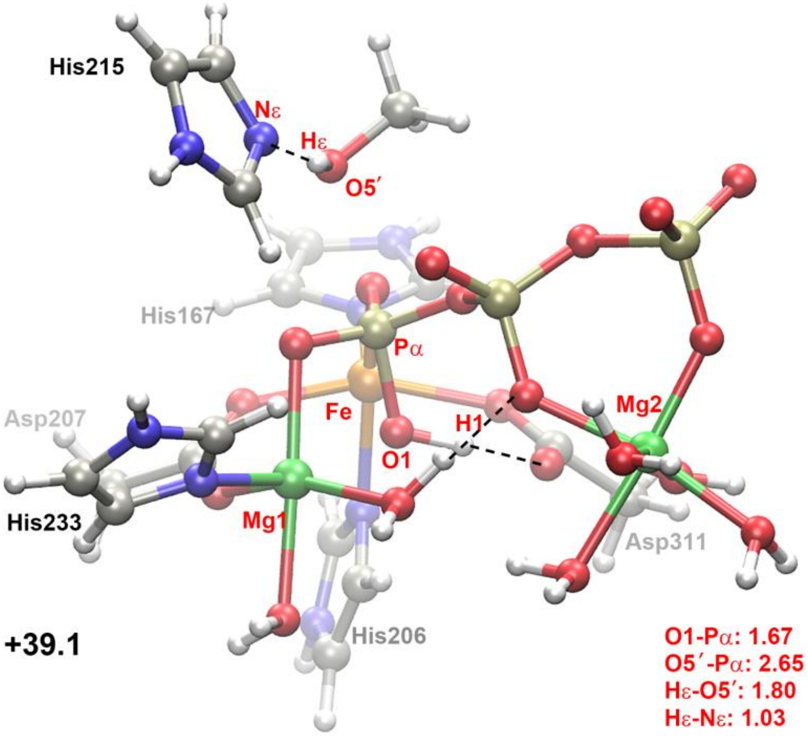

Figure S15. The optimized structure with the Pα−O1 bond distance of 1.67 Å, to gauge the energy of a possible product state in the Fe^{3+}-substituted model. The iron ion in this structure is ferric. We constrained the Pα−O1 bond distance to 1.67 Å in this state based on the obtained product state in Model-1 (see **Figure 3**), and then optimized it. The energy of this structure was calculated to be 39.1 kcal/mol relative to **RS** of Model-2 and shown in the lower left corner. All distances are given in Å and shown in the lower right corner. Key hydrogen bonds are represented by dashed lines.

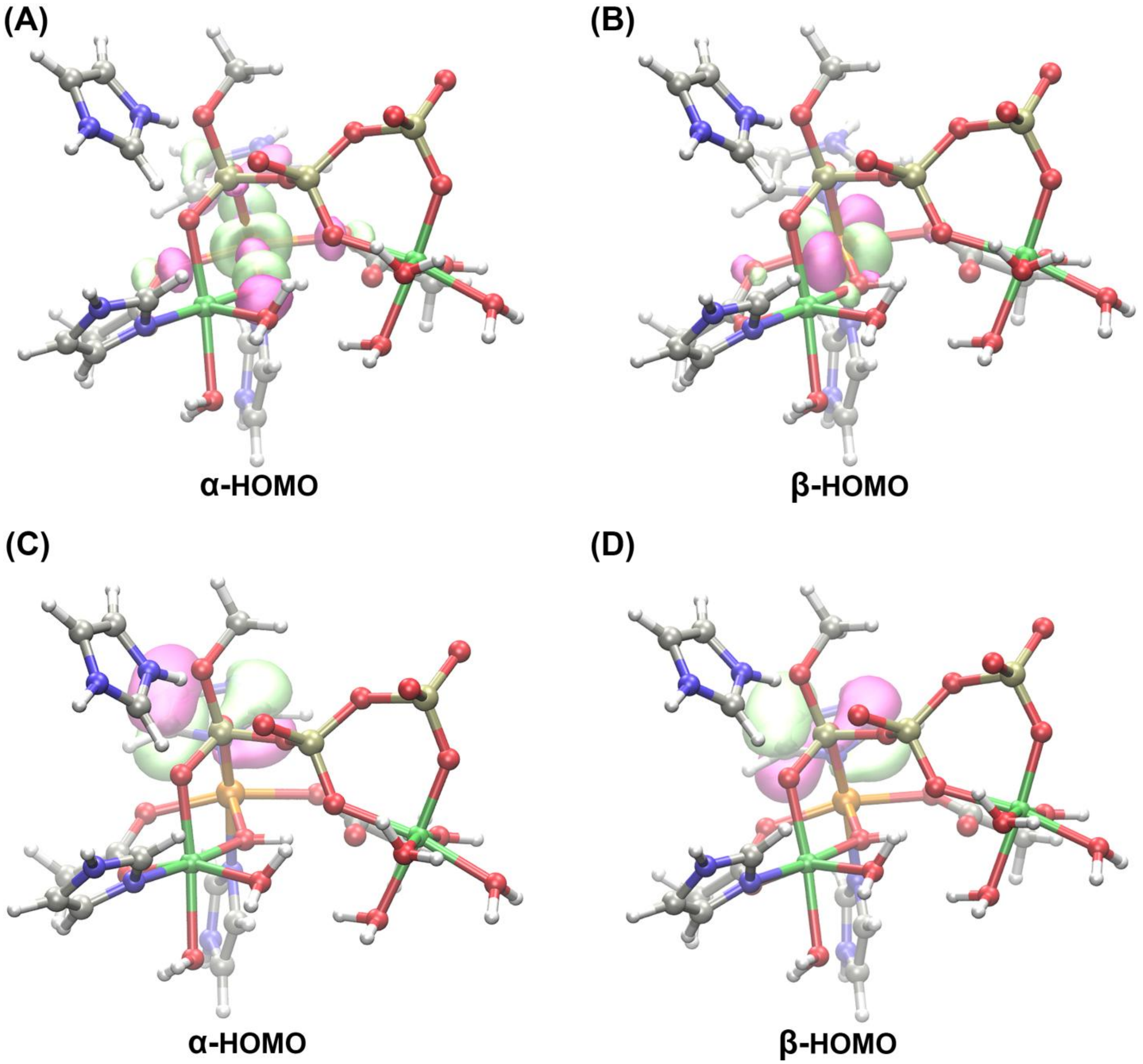

Figure S16. (A) and (B) HOMO orbitals obtained from the QM/MM calculations on Model-1, which contains a Fe^{2+} in the active site. (C) and (D) HOMO orbitals obtained from the QM/MM calculations on Model-2, which contains a Fe^{3+} in the active site. Details of structures are shown in **Figure 2.**

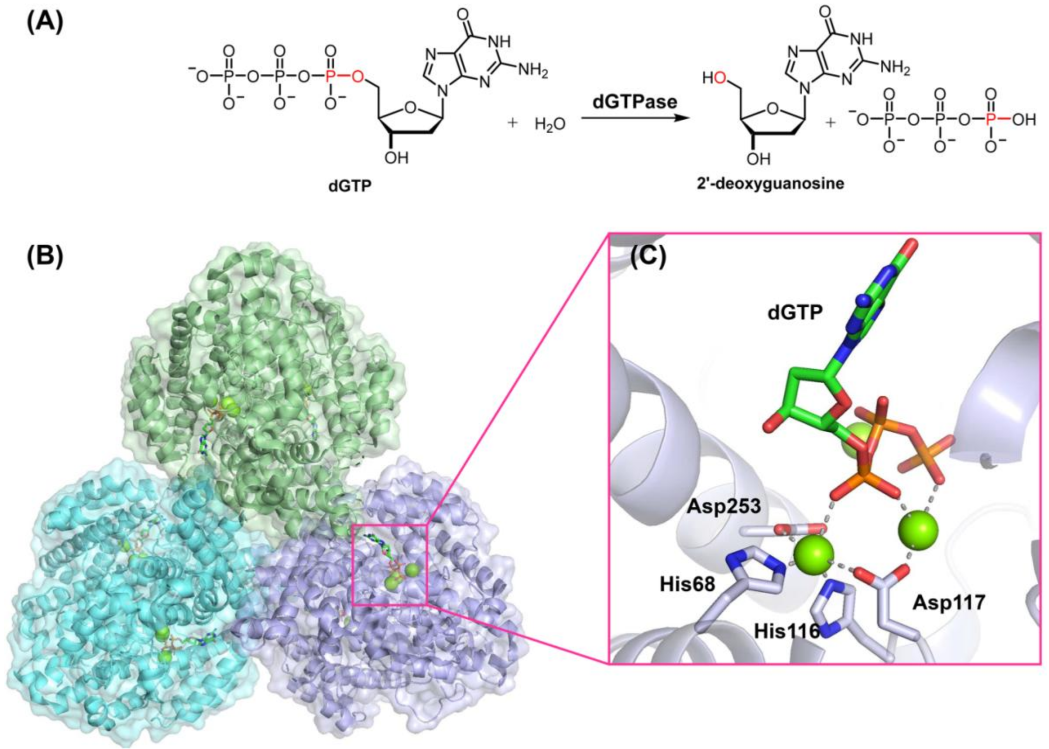

Figure S17. (A) Hydrolysis of dGTP by dGTPase from *Leeuwenhoekiella blandensis*.[3] (B) Crystal structures of hexameric L. blandensis dGTPase at 2.85 Å (PDB code 7TU7). (C) Active site bound to dGTP in a monomer. Green spheres correspond to magnesium ions.

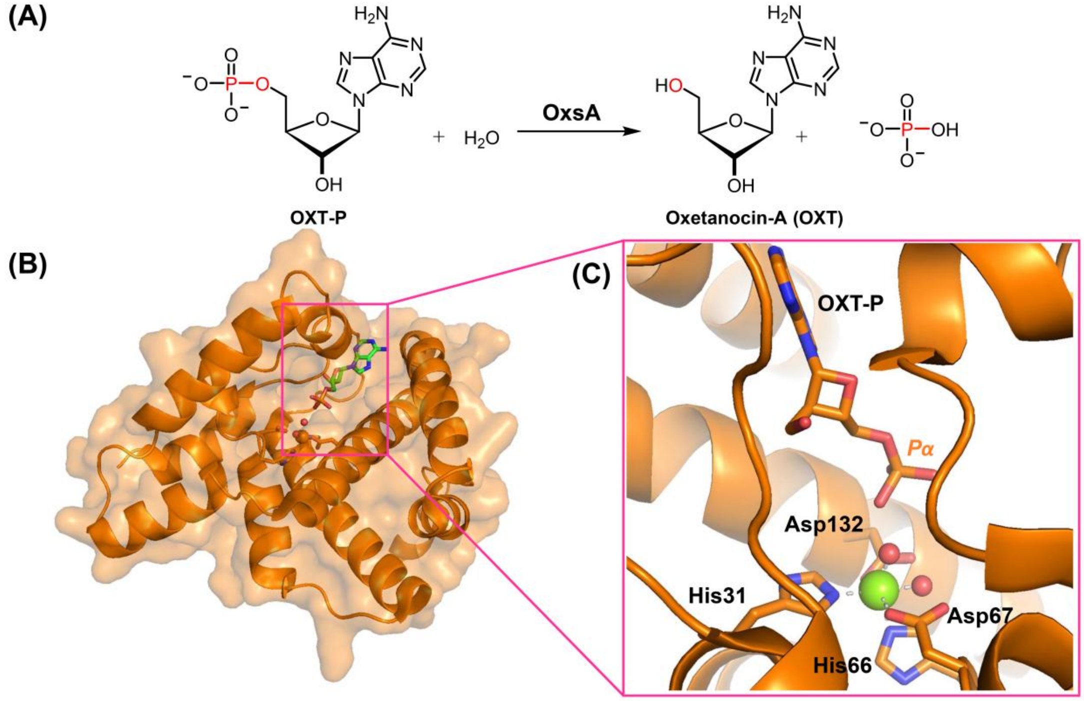

Figure S18. (A) Hydrolysis of oxetanocin-5-triphosphate (OXT-P) by OxsA from *Bacillus megaterium.*[4] (B) Crystal structures of OxsA at 1.64 Å (PDB code 5TK8). (C) Active site bound to the substrate OXT-P.[4] Green sphere corresponds to magnesium ions. Red spheres correspond to water molecules.

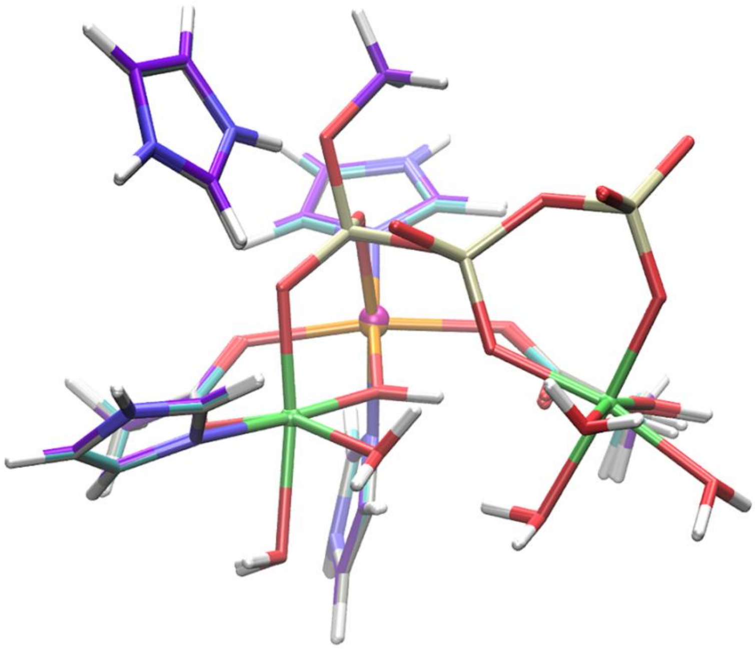

Figure S19. Overlay of the optimized structures of Model-1 (carbon atoms shown in grey), Model-3 (carbon atoms shown in cyan), and Model-4 (carbon atoms shown in purple). It demonstrates that the geometries of these optimized reactant states in different models are almost similar.

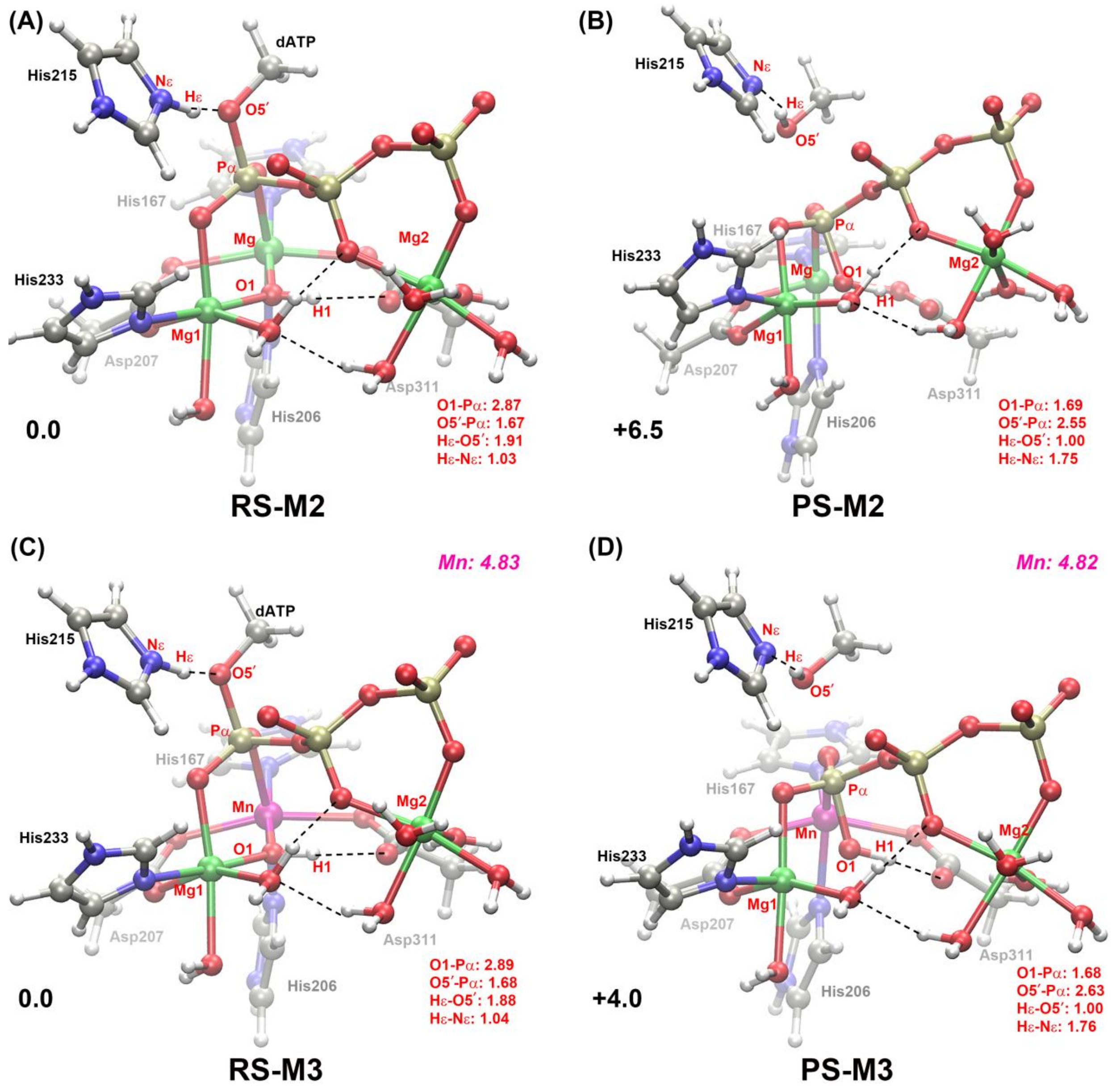

Figure S20. (A) and (B) In Model-3, Fe^{2+} is replaced by Mn^{2+}. The optimized structure of reactant state (**RS-M2**) and product state (**PS-M2**) at the B3LYP/def2-SVP:MM level. Relative energies are shown in the left corner. The energy of RS-M2 is set to the ground state. All distances are given in Å and shown in the right corner. (C) and (D) In Model-3, Fe^{2+} is replaced by Mn^{2+}. The optimized structure of reactant state (**RS-M3**) and product state (**PS-M3**) at the B3LYP/def2-SVP:MM level. Relative energies are shown in the left corner. The energy of RS-M2 is set to the ground state. All distances are given in Å and shown in the right corner. Key hydrogen bonds are represented by dashed lines.

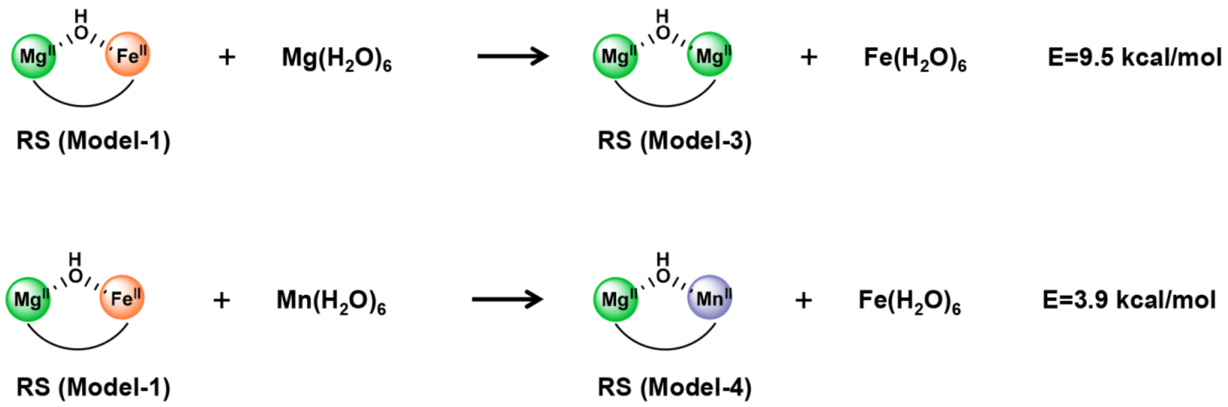

Figure S21. (A) Simulate the process, in which Fe^{2+} in the reactant state is replaced by Mg^{2+}. This process is endothermic with an energy of 9.5 kcal/mol. (B) Replacing Fe^{2+} with Mn^{2+} for RS was an endothermic process with energies of 3.9 kcal/mol.

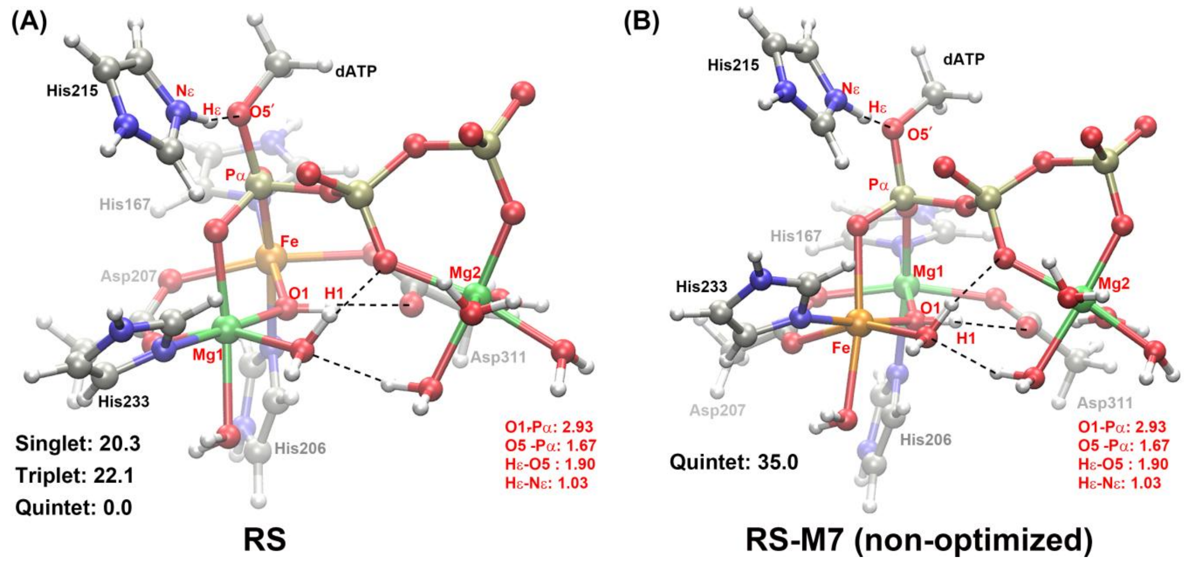

Figure S22. (A) Optimized structure of Reactant State (**RS**) at the B3LYP/def2-SVP:MM level. Key distances are given in Å (red). (B) Exchange the positions of Mg and Fe in RS of Model-1. After exchanging, single-point calculations were done for this un-optimized structure at the B3LYP/def2-TZVP:MM level. The distances of all key chemical bonds are the same as in **RS**. Energies are given in kcal/mol relative to **RS.** Key hydrogen bonds are represented by dashed lines.

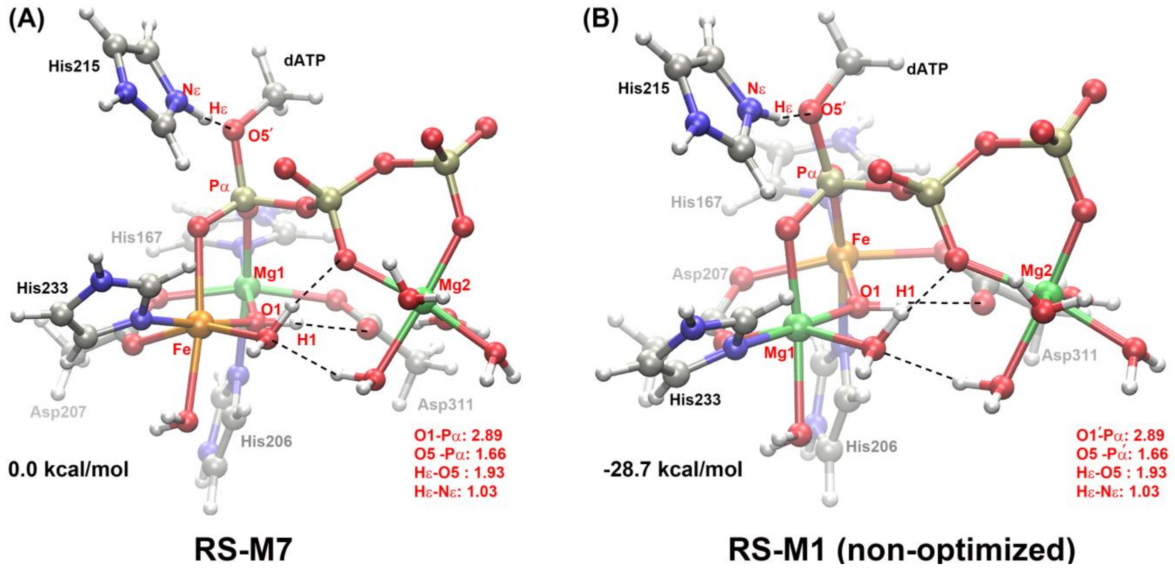

Figure S23. (A) Optimized structure of reactant state (**RS-M7**) of Model-7 at the B3LYP/def2-SVP:MM level. Key distances are given in Å (red). (B) Exchange the positions of Mg and Fe in **RS-M7** of Model-7. After exchanging, single-point calculations were done for this un-optimized structure at the B3LYP/def2-TZVP:MM level. The distances of all key chemical bonds are the same as in **RS-M7**. Energies are given in kcal/mol relative to **RS-M7.** Key hydrogen bonds are represented by dashed lines.

Table S1. Histidine residues in chain A and their protonation states, based on contacts observed in the crystal structure (PDB code 6TX0).

Residue ID	Chosen protonation	Contact	Predicted pK_a
123	HSD	water	6.78
125	HSD	water	6.17
129	HSD	water	5.19
162	HSD	Asp319	5.47
167	HSD	Fe	3.49
180	HSD	water	6.22
206	HSD	Fe	-0.31
210	HSE	dATP	-1.17
215	HSP	dATP	8.75
233	HSD	Mg	5.25
243	HSE	water	5.47
321	HSE	HSE321(B)	4.85
322	HSD	Arg318	-0.03
364	HSE	Asp361(B)	6.15
370	HSE	Gln375	3.69
376	HSE	dATP	5.87
517	HSD	water	5.66

Table S2. Relative energies of different spin states in Model-1. It shows that the quintet is the ground state when the iron is ferrous. Energies are given in kcal/mol.

	**Singlet**	**Triplet**	**Quintet**
ΔE (QM/MM)	20.3	22.1	0.0
Spin population on Fe	-	1.9	3.8

Table S3. Relative energies of different spin states in Model-2. It shows that the sextet is the ground state when the iron is ferrous. Energies are given in kcal/mol.

	**Doublet**	**Quartet**	**Sextet**
ΔE (QM/MM)	26.4	14.4	0.0
Spin population on Fe	1.0	3.0	4.3

Table S4. NPA analysis results about the charges of four selected atoms.

	**Fe**	**O1**	**Pα**	**O5'**
Model-1 (Mg/Fe^{2+})	2.60e	-0.65e	1.32e	-0.44e
Model-2 (Mg/Fe^{3+})	3.01e	-0.52e	1.32e	-0.43e

Table S5. The calculated energy barriers for different models.

	Metal in site A	Metal in site B	Multiplicity	Energy barrier (kcal/mol)
Model-1	Fe^{2+}	Mg^{2+}	5	13.4
Model-2	Fe^{3+}	Mg^{2+}	6	>40
Model-3	Mg^{2+}	Mg^{2+}	1	15.7
Model-4	Mn^{2+}	Mg^{2+}	6	13.9
Model-5	Zn^{2+}	Mg^{2+}	1	13.3
Model-6	Zn^{2+}	Zn^{2+}	1	14.7

Cartesian coordinates for all the optimized structures of QM region

RS (Model-1)

N	14.52843167	-8.46315418	9.88915806
H	13.64892289	-7.89029478	9.79505537
C	15.79799801	-7.95186561	9.76531408
H	16.01893750	-6.96053273	9.36138483
C	14.63671515	-9.72560446	10.36199265
H	13.78739736	-10.36905780	10.59604065
N	15.91616519	-10.06003988	10.53384455
C	16.65134395	-8.95424951	10.16155396
H	17.73573412	-8.95054320	10.24014869
N	17.55477657	-9.76477220	15.16334652
H	17.52208198	-8.99242631	15.83878217
C	17.95810689	-11.05567126	15.44123972
H	18.39877126	-11.38363722	16.37853434
C	16.95967182	-9.77787278	13.94708770
H	16.57282113	-8.88531004	13.45794684
N	16.95543967	-10.99829649	13.44273480
C	17.55996562	-11.80973958	14.36993366
H	17.68438610	-12.86841772	14.16338531
C	20.84065337	-10.18166984	12.24865041
H	21.45531831	-9.97044832	11.36051080
H	20.27640972	-9.26916024	12.48547649
C	19.89004444	-11.33400637	11.86031829
O	20.27815489	-12.52430212	12.08912146
O	18.83043548	-11.01068458	11.28628205
H	21.49115784	-10.46961180	13.11625879
C	20.28381794	-15.75775207	5.40361833
H	19.78877008	-15.00700280	4.79197083
C	21.40405574	-16.49834721	5.13508168
H	22.01558620	-16.55397898	4.23715969
N	19.88286319	-16.10401878	6.67658950
H	19.05621176	-15.74051539	7.17660284
N	21.65681223	-17.26158083	6.25797622
H	22.49232638	-17.89630169	6.43484022
C	20.71715139	-17.01821973	7.17154941
H	20.60994596	-17.51636941	8.12954550
N	22.77191093	-16.65845731	10.52440254
H	23.16352401	-17.43675729	9.94297603
C	23.52509897	-15.69146584	11.13724917
H	24.59995141	-15.61267061	10.99820806
C	21.48141976	-16.43458633	10.83567328
H	20.66904834	-17.09464922	10.54238912
N	21.35527222	-15.34396022	11.58097467
C	22.63113269	-14.85766115	11.76665577
H	22.81276072	-13.92961983	12.30427710
C	13.31004476	-13.38549824	13.72512637
H	12.90315858	-14.39930057	13.87254161
H	13.73835982	-13.07791952	14.69491745
C	14.47038680	-13.45150765	12.71890356
O	14.75180315	-12.49748924	11.98465827
O	15.14117193	-14.53609172	12.83285003
H	12.47785903	-12.68140018	13.48476782
Fe	16.81275745	-11.87493215	11.41697327
Mg	19.37461146	-14.35949961	11.90348470
Mg	15.39346057	-18.45219625	12.24743076
C	16.48721872	-14.24641316	6.92175970
H	15.97062949	-13.35794631	7.30971283
H	15.84928031	-15.12864181	7.09633681
O	17.76081760	-14.41266748	7.60088287
P	17.66957564	-14.23718167	9.25491696
O	17.01377882	-12.88929244	9.45517028
O	19.07240574	-14.54008198	9.76902938
O	16.62138204	-15.42108109	9.64797326
P	16.76432493	-17.06214578	9.55098988
O	16.82505998	-17.57529838	11.00592378
O	17.92271393	-17.41349042	8.64094525
O	15.34177689	-17.41549076	8.91634549
P	14.38627863	-18.82151815	9.11586204
O	14.26917202	-18.96016572	10.67660429
O	13.07504519	-18.47409313	8.44475638
O	15.17664771	-19.97730561	8.50092050
H	16.67180638	-14.11323571	5.84437859
O	17.57482968	-13.65062472	12.12220435
H	16.78930882	-14.18138506	12.35454290
O	14.31263398	-19.63537853	13.74737229
H	13.67601433	-19.15339172	14.34820237
H	14.89425317	-20.15806949	14.34439859
O	14.19796058	-16.90609649	12.86596842
H	14.52188056	-15.94851618	12.85132413
H	13.29821162	-16.91958411	13.26977893
O	16.43310691	-20.33580447	12.07530099
H	16.90019357	-20.40971375	11.20419978
H	15.73852658	-21.00598380	11.98831356
O	20.09606040	-14.55539706	14.05760023
H	20.83776883	-15.13737731	13.82810183
H	20.51835236	-13.70679615	14.29475086
O	16.78171274	-17.95825422	13.87102144
H	17.33790793	-17.18630145	13.62461270
H	17.44366501	-18.67798957	13.99316132
O	18.72979147	-16.41392021	12.43894636
H	19.41407501	-17.10946354	12.65576853
H	18.08913192	-16.85486186	11.81959730

TS (Model-1)

N	14.48664678	-8.36765557	9.81476881
H	13.60415158	-7.79434769	9.73138826
C	15.75730052	-7.86198581	9.68098328
H	15.98100967	-6.86720497	9.29333121
C	14.58986359	-9.63084421	10.27428898
H	13.74028968	-10.27415632	10.50777458
N	15.87214971	-9.97030078	10.42973676
C	16.61092004	-8.86734426	10.05910196
H	17.69657046	-8.86990183	10.11149951
N	17.49358066	-9.85102502	15.02646229
H	17.47782684	-9.05319158	15.67133255
C	17.91772723	-11.12430126	15.34312068
H	18.33549637	-11.41190687	16.30653507
C	16.93770763	-9.89568171	13.79557574
H	16.55699503	-9.01768892	13.27579133
N	16.96669543	-11.13006681	13.31424347
C	17.55916778	-11.91023970	14.27504705
H	17.69292125	-12.97969629	14.14244968

C	20.81078873	-10.22846466	12.24319316
H	21.41983734	-10.06552850	11.34150905
H	20.28782555	-9.28662896	12.45983145
C	19.80195305	-11.34181968	11.90128132
O	20.07438778	-12.53272937	12.25911383
O	18.79612860	-10.98684562	11.25255552
H	21.46053573	-10.51857667	13.10944483
C	20.24605008	-15.55143432	5.40411269
H	19.80273882	-14.82993681	4.71940592
C	21.33537637	-16.36019390	5.20065161
H	21.95990210	-16.50587307	4.31614929
N	19.77694588	-15.76644142	6.68549222
H	18.81285854	-15.15260174	7.23984106
N	21.52591759	-17.05562166	6.37793816
H	22.32344750	-17.71430287	6.58721882
C	20.56513872	-16.68216042	7.24447459
H	20.41785747	-17.09586670	8.23598945
N	22.82494910	-16.64751403	10.54178993
H	23.20400993	-17.42153434	9.94252363
C	23.58838248	-15.69369484	11.15911546
H	24.66180239	-15.61928543	11.01499076
C	21.54474921	-16.44128714	10.88846559
H	20.73024610	-17.09591369	10.58869255
N	21.43292877	-15.37134525	11.66953010
C	22.71078948	-14.88298567	11.83632333
H	22.91219704	-13.97816004	12.40580287
C	13.38021922	-13.36918926	13.66679548
H	12.99926817	-14.39651101	13.78331671
H	13.80152284	-13.08103415	14.64574497
C	14.53824521	-13.37038753	12.65478881
O	14.75591920	-12.41008699	11.90524126
O	15.27689161	-14.41521721	12.75843114
H	12.52756677	-12.67927109	13.45068907
Fe	16.74029049	-11.75402912	11.18035828
Mg	19.47878127	-14.45572229	11.91468717
Mg	15.41493739	-18.45724423	12.29267388
C	16.66119897	-14.35521857	6.99696643
H	16.05569738	-13.53709763	7.40938818
H	16.09933619	-15.29504398	7.13886954
O	17.92012377	-14.38756781	7.69807887
P	17.69891095	-14.18511084	9.66877971
O	17.03027073	-12.81047345	9.41521025
O	19.20186176	-14.49264362	9.84440369
O	16.63597886	-15.43323528	9.62786923
P	16.78461544	-17.07036485	9.60799937
O	16.81242196	-17.51364219	11.09969013
O	17.95287820	-17.49544251	8.76140800
O	15.35515846	-17.42731978	8.98899788
P	14.39219081	-18.82088150	9.18062005
O	14.26220313	-18.94528419	10.74756310
O	13.08288564	-18.47122239	8.50606241
O	15.17525968	-19.98847088	8.58260462
H	16.81183478	-14.16807652	5.91659959
O	17.51109404	-14.01801577	11.45433477
H	16.64496196	-14.31750125	11.86024579
O	14.32140869	-19.62517541	13.78073867
H	13.67308682	-19.15295123	14.37672073
H	14.89180250	-20.16062791	14.37743502
O	14.28925091	-16.85857912	12.97496121
H	14.60688442	-15.91933946	12.87918711
H	13.36546645	-16.85234325	13.32463392
O	16.41846299	-20.35605201	12.11113820
H	16.90451404	-20.44099003	11.24980676
H	15.69103509	-20.98521375	11.98742539
O	20.02200262	-14.58821346	14.09518881
H	20.72507694	-15.24025699	13.94018279
H	20.49667885	-13.76353310	14.32373205
O	16.81565369	-18.00483007	13.93111449
H	17.37973500	-17.24400606	13.67621468
H	17.46805960	-18.73598951	14.04389878
O	18.76704724	-16.44224141	12.47563226
H	19.43805997	-17.15285687	12.69279866
H	18.09594963	-16.87549046	11.87045812

PS (Model-1)

N	14.4537232654	-8.3004707301	9.7700910895
H	13.5695017553	-7.7287685751	9.6771447779
C	15.7264919025	-7.7990362654	9.6427938160
H	15.9562294134	-6.8053860201	9.2580637847
C	14.5467499986	-9.5550670061	10.2474419980
H	13.6923953644	-10.191989768	10.4802820103
N	15.8278879633	-9.8940029999	10.4223096312
C	16.5744232613	-8.7987581071	10.0425583280
H	17.6598745116	-8.8021464816	10.0976856739
N	17.5191590061	-9.7888769084	15.0776945101
H	17.5136487137	-9.0019310900	15.7370602586
C	17.9030636121	-11.0784657832	15.3793679727
H	18.3242136374	-11.3894343239	16.3337391668
C	16.9483606462	-9.8013097117	13.8548889380
H	16.5915712105	-8.9061590983	13.3480360645
N	16.9310583756	-11.0323589722	13.3629664857
C	17.5086896957	-11.8422383447	14.3097543021
H	17.6090945988	-12.9135489470	14.1672094098
C	20.8037988771	-10.2664434863	12.2736598976
H	21.4058373532	-10.1385863775	11.3621200615
H	20.3053604336	-9.3076159876	12.4735654892
C	19.7627880063	-11.3598344193	11.9728838804
O	19.9911654575	-12.5461766814	12.3718348427
O	18.7663687519	-10.9874556728	11.3162460768
H	21.4561711615	-10.5510572192	13.1385261462
C	20.2815583084	-15.7619306164	5.2100632321
H	19.8326879918	-15.0568204803	4.5120553181
C	21.4137467960	-16.5162759480	5.0213545976
H	22.0606203463	-16.6160176422	4.1522511615
N	19.7863049285	-15.9863391314	6.4743096377
H	18.5293435838	-14.9864579931	7.1998716909
N	21.6182285049	-17.1986990061	6.2004105631
H	22.4267813633	-17.8265752367	6.4379063767
C	20.6111773900	-16.8562048961	7.0396294988
H	20.4815444027	-17.2854096488	8.0299030223
N	22.8574884313	-16.6315842523	10.5168204768
H	23.2301632822	-17.3935110734	9.8970233020
C	23.6319437880	-15.7066090474	11.1627534642
H	24.7053379869	-15.6352156196	11.0182788279
C	21.5809352044	-16.4273385644	10.8763089611
H	20.7573609908	-17.0520691404	10.5397683101
N	21.4835629710	-15.3869030005	11.7010004048
C	22.7667446859	-14.9150115461	11.8761774496
H	22.9844713344	-14.0352846856	12.4785639626
C	13.3232945480	-13.3268684443	13.7116076210
H	12.9515995857	-14.3601226289	13.7995920126

H	13.7203606190	-13.0549593961	14.7051106811
C	14.4971242730	-13.2982050439	12.7317830217
O	14.7304351311	-12.3375130595	11.9925837730
O	15.2397686982	-14.3453089957	12.8533289487
H	12.4733042714	-12.6409163950	13.4843209000
Fe	16.6894784158	-11.5962352878	11.2587488669
Mg	19.5568855484	-14.5241541203	12.0887611577
Mg	15.4224843270	-18.4527776562	12.3418626985
C	16.7102858201	-14.1758165479	6.8706560880
H	16.1420292509	-13.3342627887	7.2906802233
H	16.1360366976	-15.0976098662	7.0729055993
O	17.9864808571	-14.2012204624	7.5018968192
P	17.6272600419	-14.2063314405	10.1010228432
O	16.9893213364	-12.8743550175	9.6934866542
O	19.1272903747	-14.4823987032	10.0487708346
O	16.6657919870	-15.4177228458	9.6570662881
P	16.8006192717	-17.0749254436	9.6781411118
O	16.8125378613	-17.4620649876	11.1867772783
O	17.9662799206	-17.5302402704	8.8548751837
O	15.3672613162	-17.4069756473	9.0620936775
P	14.4023258300	-18.8040970230	9.2293504609
O	14.2636116274	-18.9285658339	10.7982842527
O	13.0980571015	-18.4515919470	8.5478606508
O	15.1879779747	-19.9718337255	8.6378167571
H	16.8176126705	-14.0293399572	5.7808120813
O	17.4731016669	-14.2092869529	11.7641244176
H	16.5257982030	-14.3113979861	12.1725577238
O	14.3202934479	-19.6163399902	13.8163495330
H	13.6665352186	-19.1477172258	14.4087823231
H	14.8820555368	-20.1625264495	14.4114051045
O	14.3024566646	-16.8490134309	13.0487325243
H	14.5950592725	-15.9099559776	12.9407271476
H	13.3679764119	-16.8507583127	13.3717815340
O	16.3966818353	-20.3656230779	12.1333811235
H	16.9102871728	-20.4547993403	11.2878560107
H	15.6398473968	-20.9465768401	11.9575732180
O	20.1254679692	-14.6454947430	14.2012906591
H	20.8403171259	-15.2935789552	14.0922218182
H	20.5728583568	-13.8185499233	14.4789071961
O	16.8326247046	-18.0334913294	13.9812085421
H	17.4197141236	-17.2912267861	13.7224440805
H	17.4669772811	-18.7817657697	14.0896437661
O	18.8373240642	-16.5156503359	12.5794566734
H	19.4982169958	-17.2485884083	12.7552061882
H	18.1519147531	-16.9021091941	11.9590812289

RS (Model-2)

N	14.5809215606	-8.541072073	9.9091042845
H	13.6928800854	-7.963628989	9.8283375207
C	15.8433317821	-8.023184956	9.7426448970
H	16.0455094492	-7.030769545	9.3307106181
C	14.6975984909	-9.788138231	10.3977246127
H	13.8560983765	-10.433522388	10.6533735374
N	15.9913959458	-10.112767523	10.5456630804
C	16.7153937956	-9.007329885	10.1326406487
H	17.8002987783	-8.991690988	10.1710564617
N	17.4759182990	-9.728193672	15.0996469851
H	17.4239377736	-8.937692166	15.7556928071
C	17.9105152274	-10.994944102	15.4276168273
H	18.3247312075	-11.283864093	16.3895135737
C	16.9570683010	-9.777225383	13.8546186736
H	16.5672760552	-8.909608380	13.3242002405
N	17.0267238711	-11.014292871	13.3792163686
C	17.6027921309	-11.788075009	14.3572286822
H	17.7682325878	-12.850078743	14.2115090074
C	20.7392349128	-10.154971441	12.2556889064
H	21.3483466343	-9.928065354	11.3676200823
H	20.1559904681	-9.254715180	12.4910870043
C	19.8322032178	-11.328489439	11.8678670243
O	20.2178845626	-12.499710212	12.0904758254
O	18.7433671707	-11.031862147	11.2793335154
H	21.3973619146	-10.430412955	13.1236547683
C	20.2593510061	-15.819824179	5.3508676573
H	19.7252935520	-15.064067552	4.7811480273
C	21.4049853925	-16.503955049	5.0344906046
H	22.0072867956	-16.501516887	4.1271202829
N	19.8944474860	-16.235877899	6.6181482057
H	19.0645306067	-15.958271152	7.1567095919
N	21.7032266732	-17.303710171	6.1202001643
H	22.5595673175	-17.916877270	6.2780480659
C	20.7752764675	-17.136844840	7.0558410020
H	20.7122957401	-17.689089401	7.9909483515
N	22.7758949622	-16.632868748	10.4440572144
H	23.1749306413	-17.407448569	9.8559018270
C	23.5247078384	-15.689817080	11.0965243479
H	24.6014956445	-15.610025823	10.9751479478
C	21.4823040833	-16.405093813	10.7312842757
H	20.6707032796	-17.046549663	10.3977022284
N	21.3496246562	-15.331006606	11.5028286434
C	22.6270900660	-14.864011425	11.7293093130
H	22.8144305356	-13.955400611	12.2972470657
C	13.4928552762	-13.316278665	13.7387673527
H	13.1526965254	-14.337618167	13.9684736963
H	13.9338173622	-12.908055020	14.6651910700
C	14.6195718077	-13.401765076	12.7067749724
O	14.9607139721	-12.397125018	12.0293371248
O	15.2300652890	-14.507810563	12.7079076208
H	12.6116507633	-12.684111548	13.4762864684
Fe	16.8840794081	-11.873288003	11.3680798204
Mg	19.4144933120	-14.388622876	11.8679130760
Mg	15.4009871891	-18.502512038	12.1805455244
C	16.4606624741	-14.141084994	6.9269479135
H	15.9439367681	-13.236208557	7.2772967591
H	15.8255090964	-15.017367254	7.1348002458
O	17.7362084234	-14.280179989	7.6145851880
P	17.6537001383	-14.157884626	9.2533771169
O	17.0037478314	-12.783310916	9.5047387787
O	19.0550293831	-14.423568151	9.7773985630
O	16.6016334835	-15.308425103	9.6717611467
P	16.7228424412	-16.973096629	9.5125192836
O	16.7997784588	-17.502186963	10.9598791874
O	17.8876625130	-17.272063999	8.5928854843
O	15.3057192585	-17.290651637	8.8746647575
P	14.3490100909	-18.731867982	9.0422154495
O	14.2692053162	-18.894636486	10.5982083798
O	13.0273131351	-18.373840804	8.4053331105
O	15.1524324174	-19.848611629	8.3845193288
H	16.6505522668	-14.050480735	5.8456958512
O	17.5790986322	-13.568841671	12.0572020166
H	16.8001900264	-14.141356264	12.2591323672
O	14.3138219259	-19.666224667	13.6344124095

H	13.7119039042	-19.187011118	14.2741258270
H	14.8848202599	-20.246471900	14.1908008792
O	14.2197450747	-16.930992568	12.8221457597
H	14.5194754258	-15.991030038	12.7499043256
H	13.3116138626	-16.946085243	13.2158150587
O	16.5924630757	-20.247338851	11.9598715054
H	16.9477323961	-20.331179917	11.0375137968
H	16.2143382855	-21.127146423	12.1166100577
O	20.0981979956	-14.544929274	14.0139731542
H	20.8046815224	-15.197332871	13.8807945628
H	20.5559599761	-13.737776727	14.3239157844
O	16.7596208045	-17.984049712	13.8389781988
H	17.3007497507	-17.199683855	13.6152303556
H	17.4333671175	-18.696402801	13.9531407248
O	18.6765459693	-16.338766917	12.3836661708
H	19.3580783077	-17.031337438	12.6334605126
H	18.0325422670	-16.801004138	11.7767133669

PS (Model-2, d(P-O)=1.67 Å, unstable)

N	14.5200031745	-8.3990221680	9.8002302238
H	13.6277162578	-7.8199548799	9.7139087244
C	15.7903551691	-7.8955340602	9.6308882733
H	16.0052208590	-6.9031332638	9.2348792257
C	14.6109709939	-9.6250975191	10.3197193055
H	13.7637239441	-10.2625361160	10.5786088132
N	15.9094517278	-9.9477252116	10.4913574373
C	16.6549911648	-8.8660303157	10.0519076222
H	17.7400489205	-8.8611691546	10.0854683696
N	17.4556745296	-9.8026657733	15.1076342822
H	17.4148631338	-9.0229247388	15.7816633257
C	17.8754505746	-11.0800640109	15.4061380501
H	18.3013104091	-11.3815728290	16.3602006212
C	16.9178760489	-9.8146784648	13.8747937391
H	16.5313466257	-8.9291655268	13.3729215824
N	16.9591657554	-11.0454216645	13.3716236298
C	17.5384362804	-11.8506940885	14.3246337716
H	17.6794574531	-12.9170336594	14.1802721629
C	20.6954305986	-10.2120695176	12.2577280802
H	21.2888244849	-10.0660905034	11.3427881052
H	20.1698500174	-9.2686563955	12.4594743065
C	19.7118256620	-11.3467333078	11.9725710820
O	19.9779514668	-12.5124400589	12.3391204866
O	18.6533150152	-11.0276916147	11.3297358546
H	21.3641158716	-10.4793494806	13.1184287085
C	20.2554339474	-15.6442306583	5.2947006245
H	19.7897796309	-14.9409993496	4.6061752707
C	21.3771577082	-16.4071938639	5.0755817238
H	22.0058536960	-16.5075268999	4.1937326669
N	19.7973072307	-15.8599021575	6.5742209783
H	18.4718639200	-14.8627274370	7.2793903023
N	21.6111711160	-17.0861273440	6.2493344098
H	22.4152853235	-17.7308332025	6.4633986683
C	20.6338798148	-16.7326790729	7.1167735169
H	20.5379132711	-17.1592432221	8.1113179790
N	22.8714614473	-16.6045349209	10.4877183790
H	23.2446332670	-17.3855405446	9.8858840883
C	23.6418049436	-15.6665087424	11.1175794247
H	24.7178872738	-15.6062720616	10.9894615274
C	21.5943533650	-16.3972192841	10.8343268418
H	20.7770105808	-17.0411135832	10.5190112406
N	21.4911590475	-15.3379409851	11.6360783516
C	22.7744252540	-14.8602908389	11.8101594287
H	22.9914191123	-13.9697750880	12.3964260730
C	13.4870441614	-13.3022060617	13.7255797164
H	13.1578336378	-14.3340828155	13.9190543317
H	13.9101162816	-12.9191203194	14.6711213727
C	14.6242473209	-13.3408445759	12.7194531917
O	14.9500089815	-12.3419831956	12.0301176210
O	15.2702288991	-14.4342159480	12.7421455302
H	12.6080504868	-12.6711036224	13.4623576522
Fe	16.7338258735	-11.5911757143	11.3007486534
Mg	19.5976334669	-14.5245197254	12.0691267198
Mg	15.4067832389	-18.5546982776	12.2568353504
C	16.6372253496	-14.1558556276	6.8777463364
H	16.0150289332	-13.3291008686	7.2502855459
H	16.1175798126	-15.1000172652	7.1181171880
O	17.9105374948	-14.0731842242	7.5103388654
P	17.6016265520	-14.1091306520	10.1447957820
O	16.9491133104	-12.7482910664	9.7435831338
O	19.0951782722	-14.3567308420	10.0540981054
O	16.6043448709	-15.2826212103	9.7760413811
P	16.7444360972	-16.9790027522	9.6632060951
O	16.7609860257	-17.4152671786	11.1561452945
O	17.9335488663	-17.3240482328	8.8284018546
O	15.3234931326	-17.2603829805	9.0255027324
P	14.3641807817	-18.7041285492	9.1221784001
O	14.2620458177	-18.8883999701	10.6813758138
O	13.0481572640	-18.3332147893	8.4805729257
O	15.1728837242	-19.8105708803	8.4609403698
H	16.7542422324	-14.0492102880	5.7872421057
O	17.4755021099	-13.9647673770	11.7988935810
H	16.5577244430	-14.2834478674	12.1873416588
O	14.3052993877	-19.7119679442	13.6830071418
H	13.6974837466	-19.2305744702	14.3143555200
H	14.8728826879	-20.2939507872	14.2418313572
O	14.2837858694	-16.9522643445	12.9914217146
H	14.5220508490	-16.0330515868	12.7610262279
H	13.3409237772	-16.9642039822	13.2993566830
O	16.6068687498	-20.2771002463	11.9932010885
H	16.9866377593	-20.3317274293	11.0757867418
H	16.2307636169	-21.1656768262	12.1034223604
O	20.1824615132	-14.6382518408	14.1563042867
H	20.8826855767	-15.3072711316	14.0743093749
H	20.6307045525	-13.8367351429	14.5016362358
O	16.7752924739	-18.0782063045	13.9453435140
H	17.3210185945	-17.3023628651	13.7134026208
H	17.4451726786	-18.7992370046	14.0352359763
O	18.7525804608	-16.4116638408	12.5163116881
H	19.4085766996	-17.1411871147	12.7380350282
H	18.0630597933	-16.8391265264	11.9204617135

RS (Model-3)

N	14.5554087774	-8.4750901686	9.8873506652
H	13.6767405829	-7.9000597268	9.8020944654
C	15.8258067602	-7.9667125514	9.7670977029
H	16.0499305856	-6.9738790102	9.3698492583
C	14.6621874790	-9.7446574889	10.3426824357
H	13.8098488874	-10.3867302807	10.5690205690

N	15.9399742573	-10.0880286927	10.5072200288
C	16.6757909516	-8.9777480464	10.1487313261
H	17.7604120498	-8.9731471883	10.2253969347
N	17.5094282292	-9.7598407135	15.1718252571
H	17.4649533305	-8.9849750280	15.8437118478
C	17.9513173171	-11.0370553912	15.4530909950
H	18.3803681452	-11.3509930862	16.4010669505
C	16.9401296383	-9.7889095471	13.9429664908
H	16.5315177791	-8.9071861031	13.4511580525
N	16.9850991769	-11.0065282591	13.4328205096
C	17.5972218013	-11.8001608183	14.3701147966
H	17.7519376055	-12.8562764346	14.1666565500
C	20.7829706369	-10.1961785393	12.2742694685
H	21.3871681417	-9.9610331912	11.3852349069
H	20.1920596737	-9.3016418731	12.5152787196
C	19.8659609564	-11.3761178385	11.8832650607
O	20.2763697735	-12.5541936023	12.1253901547
O	18.8015698183	-11.0853695656	11.2964842037
H	21.4460784475	-10.4641777737	13.1395748441
C	20.2662953686	-15.7597322924	5.3821024739
H	19.7709720824	-15.0046065255	4.7755382270
C	21.3830898644	-16.5029528816	5.1100250992
H	21.9994044060	-16.5529917167	4.2131469180
N	19.8610556741	-16.1101947892	6.6532441378
H	19.0418466093	-15.7348070560	7.1610784430
N	21.6300320908	-17.2761615585	6.2277386188
H	22.4653146388	-17.9110066124	6.4023514285
C	20.6906460077	-17.0333365233	7.1418441086
H	20.5791682906	-17.5389906139	8.0955147187
N	22.7578420047	-16.6656496489	10.4908540983
H	23.1502096509	-17.4387894465	9.9021368709
C	23.5088066336	-15.7044120125	11.1155929223
H	24.5831952673	-15.6198154595	10.9786531562
C	21.4669300545	-16.4481537729	10.8056774612
H	20.6549992698	-17.1038331609	10.5017967314
N	21.3390028428	-15.3674741911	11.5646856514
C	22.6134003826	-14.8806300758	11.7554903759
H	22.7937491836	-13.9578199635	12.3024399262
C	13.3967494639	-13.3202607391	13.7569409019
H	13.0253907442	-14.3278449250	14.0036133613
H	13.8611261021	-12.9174144790	14.6739558476
C	14.5095675609	-13.4482728954	12.7016244746
O	14.8382476558	-12.4834303061	11.9940256746
O	15.0990605345	-14.5775685954	12.7447986649
H	12.5320608351	-12.6614060153	13.4995527435
Mg	16.8270730701	-11.9229203802	11.3802909972
Mg	19.3666520686	-14.3961867836	11.9128296072
Mg	15.3665845294	-18.4778542250	12.2126834665
C	16.4845479916	-14.2250189596	6.9403784237
H	15.9745993623	-13.3302280535	7.3228031918
H	15.8422252814	-15.1017647454	7.1251874941
O	17.7576792104	-14.3925981524	7.6194092929
P	17.6634106978	-14.2302941119	9.2822252245
O	17.0005850361	-12.8789346074	9.4821602569
O	19.0701867889	-14.5340271368	9.7777991503
O	16.6130032607	-15.4157551447	9.6580772844
P	16.7469898795	-17.0591068331	9.5380088073
O	16.8043584231	-17.5886654721	10.9872198802
O	17.9048887687	-17.4034296338	8.6255149319
O	15.3244058355	-17.4019976904	8.8972640481
P	14.3731012911	-18.8179020660	9.0723277927
O	14.2603310547	-18.9818703848	10.6301835833
O	13.0585035888	-18.4668459286	8.4097867983
O	15.1654571941	-19.9615870455	8.4381924869
H	16.6659003818	-14.1020159378	5.8602424950
O	17.5693239337	-13.6883776442	12.0957039985
H	16.7950815968	-14.2391513636	12.3052269598
O	14.2832827417	-19.6612207548	13.7098078581
H	13.6603405554	-19.1740123097	14.3212197666
H	14.8696003549	-20.1852098911	14.3020563880
O	14.1419234129	-16.9417683896	12.8065940259
H	14.4538419353	-15.9832709745	12.7745087067
H	13.2510410896	-16.9616023871	13.2299811549
O	16.4446480528	-20.3360658039	12.0490994846
H	16.8845036912	-20.4072276117	11.1634810066
H	15.7993427157	-21.0578157729	12.0137071069
O	20.0970925813	-14.5648041927	14.0639383561
H	20.8465787500	-15.1458230896	13.8609451768
H	20.5063825695	-13.6997843152	14.2599460742
O	16.7377595051	-17.9692272162	13.8499317957
H	17.2940030099	-17.1970548345	13.6050010787
H	17.4014341749	-18.6865917628	13.9740837948
O	18.7148295621	-16.4456833530	12.4341299079
H	19.3963845821	-17.1456839210	12.6458878832
H	18.0732134732	-16.8791170231	11.8108363663

TS (Model-3)

N	14.4912069317	-8.3714765283	9.8091549763
H	13.6104133773	-7.7965536386	9.7221638373
C	15.7626057681	-7.8680170162	9.6849102021
H	15.9907737615	-6.8740245482	9.2976346219
C	14.5906650835	-9.6353681373	10.2704638070
H	13.7363653477	-10.2767458175	10.4915265535
N	15.8700379954	-9.9788588355	10.4383228250
C	16.6113114256	-8.8748883185	10.0711695118
H	17.6967593843	-8.8744522832	10.1284469051
N	17.4849645772	-9.8674751623	15.0598688873
H	17.4660081150	-9.0729536817	15.7089667408
C	17.9138660487	-11.1405877049	15.3693914495
H	18.3401742629	-11.4306716833	16.3275710190
C	16.9184686361	-9.9123567210	13.8325634572
H	16.5305952934	-9.0341876046	13.3184560400
N	16.9471353240	-11.1435078000	13.3458453022
C	17.5490145406	-11.9234163881	14.3008649155
H	17.6886958358	-12.9920482129	14.1653505195
C	20.7608062417	-10.2409247402	12.2425291722
H	21.3596856744	-10.0657972619	11.3363884716
H	20.2194669864	-9.3102658212	12.4626214243
C	19.7787645520	-11.3818264798	11.9151654071
O	20.0947366305	-12.5613391910	12.2677319825
O	18.7434728684	-11.0734419796	11.2861137649
H	21.4255115380	-10.5155518901	13.1036304919
C	20.2349058732	-15.5332048713	5.4618158355
H	19.7929966747	-14.8108493736	4.7773596641
C	21.3253679489	-16.3398090576	5.2524008144
H	21.9444010406	-16.4838376620	4.3655229080

N	19.7644591913	-15.7523902393	6.7414878368
H	18.7500454618	-15.1165721009	7.3106344749
N	21.5192671372	-17.0383379305	6.4257299139
H	22.3150397025	-17.7011732630	6.6295319593
C	20.5572183032	-16.6672990611	7.2930502193
H	20.4167725906	-17.0875194438	8.2828743178
N	22.8155710251	-16.6647185493	10.5646710095
H	23.1961247379	-17.4438732883	9.9730513125
C	23.5750540629	-15.6947566503	11.1614724349
H	24.6475974087	-15.6175306552	11.0128378410
C	21.5361499488	-16.4641713287	10.9171708584
H	20.7277815486	-17.1348340913	10.6372832832
N	21.4199625607	-15.3829174720	11.6819883592
C	22.6952689337	-14.8809177962	11.8322705934
H	22.8922313226	-13.9645173965	12.3842845910
C	13.4340364410	-13.3763496538	13.6557223380
H	13.0508397140	-14.3978618278	13.8075642704
H	13.8703222675	-13.0587974403	14.6188177251
C	14.5825901458	-13.4224650638	12.6377896226
O	14.8445048825	-12.4750532102	11.8825693509
O	15.2840923670	-14.4889066268	12.7468058841
H	12.5817663677	-12.6906332053	13.4284339639
Mg	16.7495858261	-11.7783658073	11.2006888154
Mg	19.4608833821	-14.4677532027	11.9244549718
Mg	15.4096453669	-18.4869654367	12.2745050967
C	16.6422408892	-14.3543670704	7.0125865955
H	16.0389339526	-13.5310100769	7.4155969871
H	16.0855101077	-15.2948127008	7.1617280661
O	17.8959352956	-14.3765423897	7.7276995044
P	17.6806918728	-14.1580865072	9.6754222397
O	17.0099937158	-12.7922978027	9.4181362085
O	19.1795935670	-14.4780484166	9.8586790421
O	16.6238778272	-15.4119874656	9.6689710706
P	16.7679195915	-17.0523938316	9.6090838769
O	16.8031552884	-17.5215086515	11.0921197507
O	17.9307710285	-17.4610994561	8.7472727363
O	15.3361527075	-17.4007371532	8.9909633675
P	14.3888885365	-18.8119179649	9.1586969665
O	14.2637144040	-18.9641338496	10.7226610313
O	13.0754673964	-18.4642405611	8.4914710153
O	15.1811076310	-19.9632795883	8.5424172069
H	16.8022601199	-14.1766350207	5.9323232206
O	17.4949630603	-13.9479625639	11.4884537087
H	16.6581837921	-14.3313262076	11.8851421151
O	14.3226969752	-19.6549937536	13.7646928817
H	13.6821370901	-19.1728433167	14.3610939559
H	14.8998920262	-20.1806271103	14.3638128415
O	14.2680811677	-16.9019758758	12.9452520609
H	14.5920432004	-15.9648691255	12.8529560406
H	13.3478784961	-16.8919242752	13.3040153443
O	16.4417831823	-20.3635164927	12.0912920022
H	16.9118852052	-20.4430882140	11.2202805599
H	15.7418727554	-21.0277748726	11.9980955943
O	20.0114210372	-14.5991455150	14.1040809315
H	20.7179664330	-15.2449008324	13.9381152832
H	20.4837005518	-13.7691494817	14.3175236088
O	16.7990621047	-18.0137076313	13.9190933411
H	17.3548983808	-17.2469965792	13.6644323204
H	17.4592979165	-18.7371542520	14.0341975039
O	18.7386257539	-16.4376992444	12.4675010785
H	19.4123247097	-17.1438769415	12.6913992481
H	18.0708069078	-16.8774244961	11.8610362432

PS (Model-3)

N	14.4606067736	-8.3015796942	9.7601924510
H	13.5790093441	-7.7272421488	9.6633866518
C	15.7335611622	-7.8017044728	9.6436506540
H	15.9664672224	-6.8081971533	9.2601072583
C	14.5500171661	-9.5586953179	10.2342175064
H	13.6916325157	-10.1951095256	10.4539588004
N	15.8280647953	-9.9021552335	10.4184700917
C	16.5770593398	-8.8047367975	10.0470397456
H	17.6624062303	-8.8052794622	10.1078169337
N	17.5040136441	-9.7764916216	15.1187997719
H	17.4949627819	-8.9969671565	15.7872716613
C	17.8819274060	-11.0700832514	15.4084673492
H	18.3062295578	-11.3918324190	16.3577518582
C	16.9274106250	-9.7790000670	13.8964967938
H	16.5702105933	-8.8777521514	13.4006848177
N	16.9030594270	-11.0031905673	13.3921429597
C	17.4800984363	-11.8215408499	14.3321962279
H	17.5794418442	-12.8921746723	14.1797846709
C	20.7326228132	-10.2965517664	12.2860209818
H	21.3226181727	-10.1485667050	11.3695585989
H	20.2013423318	-9.3559242908	12.4885339606
C	19.7362753940	-11.4341678495	12.0019392644
O	20.0282834764	-12.6051697217	12.3981176274
O	18.7049101724	-11.1277146546	11.3633947694
H	21.4030639951	-10.5552403634	13.1463091136
C	20.2806949503	-15.7480257341	5.2259133408
H	19.8332321336	-15.0394521365	4.5304051264
C	21.4068152063	-16.5100630660	5.0311185866
H	22.0504145538	-16.6115936357	4.1597730106
N	19.7905361819	-15.9702133485	6.4925282178
H	18.5219123808	-15.0090265436	7.2194484946
N	21.6124155688	-17.1947388876	6.2086070890
H	22.4181489980	-17.8270529552	6.4432057208
C	20.6119397813	-16.8461249019	7.0530243162
H	20.4843752731	-17.2754038315	8.0433356818
N	22.8346869358	-16.6597587261	10.5170228391
H	23.2132326572	-17.4205622885	9.8994214652
C	23.6019349818	-15.7265708382	11.1600944361
H	24.6744021246	-15.6459414407	11.0143518701
C	21.5568557082	-16.4659139245	10.8783195369
H	20.7379505100	-17.0989968680	10.5460828610
N	21.4518985258	-15.4247094031	11.7003272962
C	22.7309518497	-14.9410044979	11.8729191405
H	22.9402301709	-14.0566596035	12.4714334955
C	13.4096795492	-13.3274757227	13.7404986715
H	13.0369443956	-14.3514924355	13.8992334043
H	13.8310959485	-12.9944639628	14.7049793900
C	14.5659581180	-13.3744075236	12.7411265951
O	14.8557101888	-12.4303422872	11.9944404394
O	15.2531790281	-14.4540348774	12.8550976453
H	12.5548434748	-12.6543413691	13.4922414302
Mg	16.6827496469	-11.6255113402	11.2677427853
Mg	19.5296144887	-14.5572688634	12.0845233949
Mg	15.4045271779	-18.4994070802	12.3225310801

C	16.6888764556	-14.2151262531	6.8876223952
H	16.1165982704	-13.3759604386	7.3056067500
H	16.1139572347	-15.1391814269	7.0781009877
O	17.9577329960	-14.2418596460	7.5354062222
P	17.6206296119	-14.1755684981	10.0633480582
O	16.9865402256	-12.8497020474	9.6466305816
O	19.1165131696	-14.4828018226	10.0398797709
O	16.6419755723	-15.4043389935	9.7007710350
P	16.7852873601	-17.0606052195	9.6798123883
O	16.7936540900	-17.4826985334	11.1805014828
O	17.9559285697	-17.4948179785	8.8519188971
O	15.3550653218	-17.3899516291	9.0551378840
P	14.3965686802	-18.7958259204	9.2001408098
O	14.2607411866	-18.9489982991	10.7656116923
O	13.0892836044	-18.4378853128	8.5271797148
O	15.1862418366	-19.9496239071	8.5874225302
H	16.8087128474	-14.0624475470	5.7996468097
O	17.4645812479	-14.1091907768	11.7461909509
H	16.5491123244	-14.3499584402	12.1502742936
O	14.3014114553	-19.6705891937	13.7972771711
H	13.6609712214	-19.1908078439	14.3952740361
H	14.8693345248	-20.2112076612	14.3922489180
O	14.2675902388	-16.9155234747	13.0309928934
H	14.5685345394	-15.9787685190	12.9282682087
H	13.3333724355	-16.9144531782	13.3546485330
O	16.4406114963	-20.3696858747	12.1248838900
H	16.9147865639	-20.4475162919	11.2549920638
H	15.7500590559	-21.0437122391	12.0310621484
O	20.1103439303	-14.6793521593	14.2106164883
H	20.8322401818	-15.3181515947	14.0941705601
H	20.5519284827	-13.8412646955	14.4616236165
O	16.7985414389	-18.0589011565	13.9806540110
H	17.3763747969	-17.3086798699	13.7248144780
H	17.4418046228	-18.7986583162	14.0906836376
O	18.7846352113	-16.5203361199	12.5755281389
H	19.4519841142	-17.2438871683	12.7665218662
H	18.1060654858	-16.9238031536	11.9543558934

RS (Model-4)

N	14.5008077478	-8.4124644105	9.8465840308
H	13.6248389301	-7.8308529446	9.7691088033
C	15.7767958123	-7.9189697523	9.7239115225
H	16.0128973453	-6.9257497724	9.3368609183
C	14.5935115814	-9.6850535774	10.2937491907
H	13.7348729019	-10.3179471293	10.5231550865
N	15.8679090910	-10.0426014624	10.4484345199
C	16.6167533734	-8.9408126580	10.0958692555
H	17.7019299018	-8.9502467897	10.1689541471
N	17.5504945403	-9.7510492636	15.2126474869
H	17.5024442963	-8.9902299384	15.9014345454
C	17.9904069484	-11.0351057333	15.4647571528
H	18.4236501560	-11.3690348053	16.4040432295
C	16.9684517203	-9.7532613717	13.9902792167
H	16.5558016233	-8.8611098804	13.5214471388
N	17.0041593385	-10.9607696145	13.4573526846
C	17.6247401124	-11.7754442709	14.3700975709
H	17.7707018243	-12.8287796013	14.1446455265
C	20.8426911957	-10.1883847252	12.2842350193
H	21.4508195164	-9.9581445186	11.3965335274
H	20.2624595469	-9.2873214719	12.5269781761
C	19.9072261275	-11.3490302631	11.8821976623
O	20.2761101517	-12.5386928716	12.1437562151
O	18.8715964203	-11.0290483241	11.2628331114
H	21.4990930372	-10.4691694691	13.1495235322
C	20.2819225211	-15.7894744647	5.3488849789
H	19.7888196552	-15.0314374564	4.7440137183
C	21.4013056926	-16.5290112965	5.0783470076
H	22.0217574241	-16.5722525343	4.1842776068
N	19.8698411804	-16.1488111827	6.6152137784
H	19.0483112814	-15.7708565083	7.1199190732
N	21.6433588943	-17.3089461627	6.1924500491
H	22.4810234032	-17.9393811533	6.3710016502
C	20.6982054041	-17.0734686810	7.1027847459
H	20.5823974781	-17.5862997497	8.0523905485
N	22.7722680302	-16.6560138768	10.4716443017
H	23.1648577625	-17.4288578415	9.8826625196
C	23.5227117240	-15.7015562040	11.1076950013
H	24.5978472678	-15.6173594860	10.9752904326
C	21.4796728106	-16.4331288001	10.7755600976
H	20.6666500755	-17.0814843276	10.4594449639
N	21.3503899541	-15.3552105378	11.5382272199
C	22.6253960994	-14.8761340553	11.7429916898
H	22.8059033277	-13.9571470966	12.2963965307
C	13.2844626682	-13.4019173483	13.7710891676
H	12.8773877341	-14.4182663137	13.8982469923
H	13.6963775892	-13.1071034364	14.7518060351
C	14.4566102691	-13.4526446943	12.7790456242
O	14.7393245310	-12.4803262688	12.0655195019
O	15.1237859775	-14.5382508451	12.8725273488
H	12.4560248201	-12.6950271325	13.5254793805
Mn	16.7931935943	-11.8803432362	11.3625576232
Mg	19.3757776314	-14.3908866604	11.8874417019
Mg	15.3708041862	-18.4634805049	12.2202744033
C	16.5023573609	-14.3008418911	6.8988366032
H	15.9876426972	-13.4151595491	7.2954252793
H	15.8655964423	-15.1842814098	7.0719142365
O	17.7776317701	-14.4680778859	7.5746692157
P	17.6777347254	-14.2669521502	9.2347326856
O	17.0092899454	-12.9171549084	9.3943424669
O	19.0830188812	-14.5475497228	9.7493543692
O	16.6291359130	-15.4467113594	9.6337517118
P	16.7553351036	-17.0899795655	9.5270817873
O	16.8139664665	-17.6063319129	10.9808590740
O	17.9082545173	-17.4474006500	8.6136196494
O	15.3279597159	-17.4296207709	8.8941612653
P	14.3658692218	-18.8338232716	9.0879427641
O	14.2499330915	-18.9767446704	10.6484080099
O	13.0544388103	-18.4814448508	8.4195042152
O	15.1509821403	-19.9907050792	8.4682619636
H	16.6822050110	-14.1596548103	5.8212744686
O	17.5684321104	-13.7129324535	12.0709925594
H	16.7850385364	-14.2354957355	12.3220215053
O	14.2898818827	-19.6379158205	13.7256293507
H	13.6600367853	-19.1548287079	14.3330310170

H	14.8743589375	-20.1621284080	14.3189874067
O	14.1644080906	-16.9107764661	12.8165942540
H	14.4970585519	-15.9576071876	12.8415786009
H	13.2736701714	-16.9334973679	13.2394530153
O	16.4196771010	-20.3423264949	12.0597355645
H	16.8751304187	-20.4159637824	11.1824563713
H	15.7421399699	-21.0318132836	11.9935961261
O	20.1049606706	-14.5597479772	14.0369804296
H	20.8572613709	-15.1395758606	13.8414203922
H	20.5091665207	-13.6859169416	14.1998077084
O	16.7544483397	-17.9537720405	13.8471722581
H	17.3177416964	-17.1901250763	13.5898513092
H	17.4127708724	-18.6760761269	13.9738156174
O	18.7314856653	-16.4484034784	12.4145430795
H	19.4145133243	-17.1465235598	12.6281712522
H	18.0910196422	-16.8846322054	11.7931161717

TS (Model-4)

N	14.4504908980	-8.2954691636	9.7561165607
H	13.5695963607	-7.7186146558	9.6719180267
C	15.7253788741	-7.7998285621	9.6379316532
H	15.9627922630	-6.8050030274	9.2618069298
C	14.5390405512	-9.5606018887	10.2127590399
H	13.6820828785	-10.1986145107	10.4337070496
N	15.8159310850	-9.9096867875	10.3803087975
C	16.5672630700	-8.8114052130	10.0227476608
H	17.6526607931	-8.8181430073	10.0859814364
N	17.5152320613	-9.8527575368	15.1008413443
H	17.4921910070	-9.0713318636	15.7668101059
C	17.9379438829	-11.1333220448	15.3874059020
H	18.3672981757	-11.4406816499	16.3388898032
C	16.9388699141	-9.8740609146	13.8782219107
H	16.5501241615	-8.9851529916	13.3834130060
N	16.9558201176	-11.0976093248	13.3736919796
C	17.5620395178	-11.8970402094	14.3091598687
H	17.6939084016	-12.9639685302	14.1526210791
C	20.8141525409	-10.2405050799	12.2605599426
H	21.4165757776	-10.0703531303	11.3559268146
H	20.2836992098	-9.3037979594	12.4813173414
C	19.8153216879	-11.3621779613	11.9228288854
O	20.0936795476	-12.5513288882	12.2777782523
O	18.8034267064	-11.0198053084	11.2751591159
H	21.4705897067	-10.5260471313	13.1227620078
C	20.2439343051	-15.5519376686	5.4598778413
H	19.8009893769	-14.8261019065	4.7798925297
C	21.3345610625	-16.3571213221	5.2493249981
H	21.9538883453	-16.4962992051	4.3625995289
N	19.7739871603	-15.7793041522	6.7375737941
H	18.7845768253	-15.1586329857	7.2866378328
N	21.5274538620	-17.0613363913	6.4198339684
H	22.3245293946	-17.7233906751	6.6230309122
C	20.5650550717	-16.6963715446	7.2880849874
H	20.4210700843	-17.1205011445	8.2757135225
N	22.8266504208	-16.6637779382	10.5651929299
H	23.2068111675	-17.4435460580	9.9742978917
C	23.5858987742	-15.6929103682	11.1609421390
H	24.6586705550	-15.6165778732	11.0136897537
C	21.5470077704	-16.4624918723	10.9165692353
H	20.7384273227	-17.1328426238	10.6366428816
N	21.4308377408	-15.3800155561	11.6797124956
C	22.7059686385	-14.8779917982	11.8299905172
H	22.9028614562	-13.9609989362	12.3810948777
C	13.3866359808	-13.4060851484	13.6687177093
H	12.9943111604	-14.4300293858	13.7768930299
H	13.8058795544	-13.1274745202	14.6512139296
C	14.5467578824	-13.4129183364	12.6651335847
O	14.7754308620	-12.4480345387	11.9225929159
O	15.2774259557	-14.4612420260	12.7608404685
H	12.5453672294	-12.7045807313	13.4486627475
Mn	16.7260660753	-11.7095264919	11.1812096747
Mg	19.4681818162	-14.4711717747	11.9116194838
Mg	15.4147562248	-18.4898802885	12.2641583005
C	16.6561252553	-14.3746723545	6.9986976083
H	16.0519066206	-13.5533423757	7.4054431186
H	16.0957181944	-15.3141102735	7.1440589156
O	17.9097238097	-14.4022831117	7.7091546146
P	17.6886982274	-14.1941036015	9.6634797240
O	17.0140411340	-12.8324530806	9.3961103491
O	19.1951171847	-14.4916306649	9.8425813124
O	16.6342006289	-15.4520839637	9.6304408616
P	16.7802052301	-17.0880253091	9.5900844432
O	16.8159140103	-17.5464548843	11.0765898615
O	17.9423183253	-17.5067201933	8.7309062626
O	15.3474439866	-17.4390859989	8.9738297393
P	14.3908941928	-18.8389548214	9.1520553745
O	14.2655504161	-18.9811299738	10.7175400778
O	13.0791356979	-18.4861054081	8.4834517247
O	15.1744093241	-19.9997892596	8.5414852358
H	16.8131880673	-14.1927628981	5.9189265666
O	17.5044293718	-14.0295752588	11.4589952027
H	16.6444574181	-14.3421614199	11.8663438594
O	14.3238194864	-19.6494075480	13.7597605447
H	13.6824445928	-19.1683144793	14.3560202914
H	14.8985200968	-20.1780558519	14.3585583236
O	14.2745303687	-16.8941274509	12.9238612232
H	14.6013513763	-15.9563836781	12.8478871214
H	13.3576749195	-16.8860803483	13.2907290680
O	16.4392863472	-20.3748278120	12.0897913778
H	16.9123683514	-20.4555421172	11.2206390126
H	15.7322477078	-21.0304829631	11.9892991942
O	20.0106619815	-14.5991656997	14.0919124286
H	20.7198438584	-15.2418351128	13.9245967691
H	20.4799472486	-13.7667928287	14.3009418144
O	16.8036500260	-18.0151102060	13.9095146610
H	17.3658103091	-17.2542025312	13.6501155610
H	17.4590211670	-18.7421987697	14.0287525338
O	18.7578976926	-16.4581967311	12.4600692412
H	19.4273976589	-17.1677611472	12.6850529816
H	18.0924740991	-16.8933654707	11.8497035056

PS (Model-4)

N	14.4147048645	-8.2264516397	9.6959043038

H	13.5317777026	-7.6514094737	9.6081559769
C	15.6909008846	-7.7335334518	9.5842532626
H	15.9324125705	-6.7373814508	9.2158399707
C	14.4948116492	-9.4881952912	10.1549500930
H	13.6330910328	-10.1204393355	10.3734460214
N	15.7713988133	-9.8399764636	10.3311784144
C	16.5286409120	-8.7446851005	9.9749720431
H	17.6141933563	-8.7532894443	10.0347227320
N	17.5356980076	-9.7542070540	15.1395534820
H	17.5272783744	-8.9833139460	15.8184545555
C	17.9008470995	-11.0548303383	15.4139784627
H	18.3245753727	-11.3907116808	16.3588114550
C	16.9508667905	-9.7374705130	13.9220594292
H	16.5994548729	-8.8266660017	13.4399771246
N	16.9091026477	-10.9562625447	13.4065852915
C	17.4853068789	-11.7909829254	14.3326149699
H	17.5713727748	-12.8611614988	14.1691186934
C	20.7802584177	-10.3055294092	12.2965675557
H	21.3751694595	-10.1666576214	11.3819553388
H	20.2602245920	-9.3579206459	12.4955853177
C	19.7669633886	-11.4258593485	12.0058038217
O	20.0230653722	-12.6024513155	12.4113540401
O	18.7585124352	-11.0913615149	11.3454896923
H	21.4426021304	-10.5719372673	13.1595681926
C	20.2854495352	-15.8063788151	5.1755985891
H	19.8368414628	-15.1002518159	4.4784524591
C	21.4244412646	-16.5508043879	4.9913653049
H	22.0760238763	-16.6417893066	4.1252759640
N	19.7830163435	-16.0418988740	6.4349183522
H	18.5379069416	-15.0332051730	7.1625048560
N	21.6263374068	-17.2377364138	6.1685312904
H	22.4379668451	-17.8594825401	6.4111625423
C	20.6108579724	-16.9078447319	7.0021644752
H	20.4775762074	-17.3430137748	7.9895479848
N	22.8496089339	-16.6577872819	10.5119975367
H	23.2278766077	-17.4151679932	9.8900474891
C	23.6175784815	-15.7298406738	11.1618908279
H	24.6900122227	-15.6482232006	11.0167520667
C	21.5715757504	-16.4642403853	10.8730614384
H	20.7516862708	-17.0919365690	10.5329420929
N	21.4674687009	-15.4283871104	11.7022363993
C	22.7468642778	-14.9478972379	11.8790550076
H	22.9573108110	-14.0683857681	12.4842727242
C	13.3507950875	-13.3575267409	13.7431708743
H	12.9666878987	-14.3846656468	13.8474363468
H	13.7509175869	-13.0740933565	14.7321129743
C	14.5251522042	-13.3622904868	12.7650923311
O	14.7799098122	-12.4099689773	12.0192441338
O	15.2496495242	-14.4179553951	12.8869228484
H	12.5088675046	-12.6646697406	13.5048185472
Mn	16.6543262453	-11.5562898354	11.2331738467
Mg	19.5420175787	-14.5672574321	12.0908940047
Mg	15.4106558984	-18.4917449454	12.3359533397
C	16.7255551564	-14.2056945676	6.8491015179
H	16.1645990551	-13.3663078864	7.2823921389
H	16.1469377819	-15.1269298607	7.0419185309
O	18.0034011788	-14.2479345982	7.4756611012
P	17.6319818983	-14.2218325372	10.0768070679
O	16.9961325551	-12.9056096267	9.6399101706
O	19.1328325851	-14.5044007600	10.0465219171
O	16.6764218028	-15.4488152516	9.6527505941
P	16.8024912150	-17.1025915886	9.6810610176
O	16.8018656677	-17.4890871569	11.1908911914
O	17.9710525529	-17.5716979083	8.8689166322
O	15.3714527325	-17.4313214993	9.0570395893
P	14.4001150464	-18.8236912936	9.2174560701
O	14.2601224941	-18.9568488869	10.7853851559
O	13.0971958580	-18.4616028940	8.5378506601
O	15.1805421041	-19.9919315425	8.6196995654
H	16.8301857306	-14.0468870971	5.7607476444
O	17.4683756186	-14.2099742770	11.7454114784
H	16.5379988270	-14.3596700125	12.1596130528
O	14.3061183995	-19.6560957475	13.8132854750
H	13.6612538861	-19.1778152433	14.4075359909
H	14.8711639293	-20.1977356111	14.4096782403
O	14.2830056105	-16.8980513264	13.0438018029
H	14.5876999237	-15.9607137640	12.9558881160
H	13.3498988334	-16.8959747312	13.3703231360
O	16.4141303539	-20.3852814505	12.1360550972
H	16.9083802995	-20.4698387865	11.2783105033
H	15.6869457963	-21.0127710896	12.0015931864
O	20.1188990965	-14.6844648655	14.2140863497
H	20.8392507808	-15.3256921723	14.1018597531
H	20.5612844473	-13.8479208327	14.4682948838
O	16.8137841996	-18.0607811504	13.9863961131
H	17.3990381465	-17.3177038372	13.7253365282
H	17.4509890869	-18.8060135007	14.0966272045
O	18.8151989914	-16.5455245501	12.5846830391
H	19.4797382621	-17.2739488449	12.7655220760
H	18.1321020986	-16.9371262454	11.9630483547

RS (Model-5)

N	14.5649672971	-8.5180746045	9.9200574822
H	13.6847465247	-7.9440708479	9.8345682479
C	15.8332580671	-8.0059994854	9.7874050881
H	16.0507549451	-7.0138573765	9.3834063051
C	14.6761608881	-9.7831363352	10.3844191285
H	13.8300394082	-10.4275151205	10.6256736221
N	15.9576899032	-10.1172147975	10.5408664810
C	16.6907759893	-9.0097705343	10.1701603999
H	17.7749863135	-9.0081040637	10.2440499106
N	17.5296932576	-9.7607882343	15.1618854217
H	17.4847821015	-8.9890776908	15.8377646220
C	17.9752386602	-11.0385478077	15.4355628953
H	18.4058799151	-11.3558774915	16.3817017282
C	16.9574631121	-9.7833295433	13.9351446518
H	16.5461360433	-8.9004645188	13.4478863870
N	17.0041066564	-10.9990900063	13.4216505922
C	17.6203555097	-11.7971385481	14.3500087290
H	17.7723055299	-12.8522654143	14.1417685910
C	20.8435415752	-10.1595351237	12.2652490204
H	21.4569275125	-9.9456770339	11.3770531416
H	20.2777560124	-9.2479492737	12.5021629727
C	19.8918826327	-11.3110705455	11.8730117433
O	20.2624325887	-12.5020145598	12.1271751629

O	18.8479771839	-10.9835721994	11.2740360991
H	21.4950751276	-10.4454424467	13.1327319539
C	20.2688712183	-15.7530605686	5.3731881770
H	19.7761693124	-14.9990684257	4.7631171865
C	21.3849031187	-16.4989995942	5.1058563245
H	22.0032053116	-16.5524310459	4.2106559528
N	19.8589682801	-16.0995246616	6.6437322837
H	19.0384374291	-15.7170831155	7.1473199996
N	21.6271452247	-17.2699180625	6.2260860324
H	22.4612563293	-17.9051136590	6.4042098210
C	20.6852554359	-17.0229567390	7.1368093224
H	20.5695443421	-17.5266075060	8.0910428103
N	22.7516815916	-16.6367888526	10.4889007111
H	23.1407398861	-17.4126240943	9.9019053114
C	23.5084946641	-15.6830278735	11.1182168196
H	24.5836161334	-15.6050214398	10.9813371212
C	21.4610897500	-16.4067875318	10.7958398108
H	20.6444085775	-17.0540578843	10.4868285416
N	21.3387255240	-15.3246070689	11.5541031903
C	22.6168740671	-14.8504576504	11.7524364054
H	22.8033153083	-13.9302338706	12.3017070733
C	13.3050170585	-13.3926073207	13.7427412557
H	12.9059501821	-14.4124792343	13.8657146366
H	13.7153727453	-13.0976494906	14.7242488191
C	14.4784715798	-13.4266136765	12.7495436556
O	14.7631312648	-12.4454390182	12.0518341727
O	15.1457873004	-14.5147174136	12.8322728857
H	12.4708042091	-12.6917604829	13.5010151870
Zn	16.8193731458	-11.8622181355	11.4021826791
Mg	19.3604773638	-14.3383700104	11.8808248018
Mg	15.3806690105	-18.4395628881	12.2222872197
C	16.4894765390	-14.2475455989	6.9144083649
H	15.9750341706	-13.3564641208	7.2992349460
H	15.8500939916	-15.1275913600	7.0951758567
O	17.7612317941	-14.4093084190	7.5955174440
P	17.6576329981	-14.2120343638	9.2588467673
O	16.9886148189	-12.8644814813	9.4237855545
O	19.0679106456	-14.5041031072	9.7583680359
O	16.6149788331	-15.4041519015	9.6469990018
P	16.7513692437	-17.0450950609	9.5347047335
O	16.8137536417	-17.5663790370	10.9873174456
O	17.9060009680	-17.3973317271	8.6213116463
O	15.3261888041	-17.3959070604	8.9017492759
P	14.3744716585	-18.8076530376	9.0914798280
O	14.2606026408	-18.9559126288	10.6517539473
O	13.0598176985	-18.4628699193	8.4253764782
O	15.1648082049	-19.9593914466	8.4691267683
H	16.6723922636	-14.1191902022	5.8354959655
O	17.5504164896	-13.6441473130	12.0319746880
H	16.7609475569	-14.1658472109	12.2705198343
O	14.3010275486	-19.6213664085	13.7226917447
H	13.6681594918	-19.1413856151	14.3294776427
H	14.8859119265	-20.1450037633	14.3160918043
O	14.1776281536	-16.8871146025	12.8226393871
H	14.5091977660	-15.9331576307	12.8273850159
H	13.2840051540	-16.9064677238	13.2395027687
O	16.4290333787	-20.3188176089	12.0570921434
H	16.8868118116	-20.3933460318	11.1810480086
H	15.7450405884	-21.0013746083	11.9847969333
O	20.0796696593	-14.5256328155	14.0394527271
H	20.8166983211	-15.1162779185	13.8177632231
H	20.5061169758	-13.6678706544	14.2299593620
O	16.7607249204	-17.9352450597	13.8517462745
H	17.3167215835	-17.1643337268	13.6007137790
H	17.4236844516	-18.6540128312	13.9739997129
O	18.7092117310	-16.3901185527	12.4219077679
H	19.3943855322	-17.0865342097	12.6339264448
H	18.0695177821	-16.8303109920	11.8010990775

TS (Model-5)

N	14.5023409857	-8.3698728926	9.8109050888
H	13.6177078855	-7.7987020322	9.7217973164
C	15.7708552596	-7.8591632333	9.6856348191
H	15.9930176034	-6.8651295325	9.2953364980
C	14.6048276108	-9.6294066093	10.2798816276
H	13.7565545925	-10.2761524614	10.5076754473
N	15.8874635549	-9.9589468283	10.4490869891
C	16.6268578293	-8.8564916376	10.0787583480
H	17.7117179431	-8.8543419189	10.1425872407
N	17.4848411713	-9.8693243677	15.0175483286
H	17.4630059400	-9.0781196384	15.6706792086
C	17.9177690620	-11.1428109455	15.3216611542
H	18.3380061492	-11.4353589042	16.2824907947
C	16.9258562482	-9.9067087581	13.7878679424
H	16.5372043183	-9.0268771323	13.2774140613
N	16.9621640339	-11.1359592909	13.2961398236
C	17.5619423882	-11.9216730495	14.2469990959
H	17.7038801874	-12.9880042438	14.1000973883
C	20.8037851360	-10.2314148342	12.2333535515
H	21.4076296210	-10.0649477871	11.3292038836
H	20.2774555266	-9.2917500499	12.4519850138
C	19.7988801519	-11.3530745559	11.9046024999
O	20.0821836855	-12.5382606885	12.2671738644
O	18.7825879307	-11.0093023573	11.2644263555
H	21.4599424899	-10.5167887876	13.0961916732
C	20.2237322083	-15.5215306277	5.4892914908
H	19.7830594555	-14.8002228301	4.8028116085
C	21.3144117765	-16.3281276847	5.2786274700
H	21.9319688996	-16.4731639129	4.3914074733
N	19.7514410589	-15.7368637029	6.7687732522
H	18.6955838965	-15.0664679150	7.3642486952
N	21.5095598232	-17.0249638696	6.4522550503
H	22.3026705880	-17.6897124387	6.6547691511
C	20.5461174639	-16.6500867667	7.3187628343
H	20.4079786476	-17.0695974314	8.3092522292
N	22.8283204006	-16.6616548054	10.5749345854
H	23.2048309652	-17.4445783975	9.9855552396
C	23.5911184405	-15.6895836736	11.1637904654
H	24.6637586296	-15.6167828832	11.0146705284
C	21.5509675127	-16.4593122417	10.9327214652
H	20.7409509124	-17.1299046524	10.6576507881
N	21.4395067610	-15.3750805520	11.6944233280
C	22.7156815544	-14.8728165536	11.8361186649
H	22.9165582620	-13.9550809028	12.3845564343
C	13.4106163031	-13.3745492590	13.6490369140
H	13.0284356792	-14.4002836888	13.7724967798
H	13.8340332236	-13.0795941764	14.6249797146
C	14.5683559073	-13.3895332902	12.6411385054
O	14.8077658423	-12.4319217897	11.8941667398
O	15.2870848646	-14.4455636006	12.7511066484
H	12.5613323920	-12.6847746028	13.4260348691

Zn	16.7369211766	-11.6837852122	11.1965086642
Mg	19.4820248904	-14.4656727356	11.9402855853
Mg	15.4164273425	-18.4790608105	12.2771075508
C	16.6346971340	-14.3263849308	7.0445631430
H	16.0322083242	-13.5010428090	7.4444467821
H	16.0805765989	-15.2664222641	7.2008916680
O	17.8870106235	-14.3389728669	7.7627146247
P	17.6814471489	-14.1586779070	9.7348972581
O	17.0111092911	-12.7893544768	9.4825532217
O	19.1877583562	-14.4673919336	9.8742000997
O	16.6251979037	-15.4121523708	9.6681529149
P	16.7742482730	-17.0506385654	9.6185815206
O	16.8104337746	-17.5170099539	11.1019913494
O	17.9369922519	-17.4615982903	8.7583997270
O	15.3425609025	-17.3996058814	8.9979923077
P	14.3949601498	-18.8084492418	9.1627064482
O	14.2681748902	-18.9590442704	10.7273125206
O	13.0818649503	-18.4604315464	8.4942194871
O	15.1851667690	-19.9617376618	8.5472889926
H	16.7950536045	-14.1560531999	5.9630384396
O	17.5196123019	-14.0255852375	11.5248162937
H	16.6447883498	-14.3185671736	11.9212156442
O	14.3229607693	-19.6457268051	13.7613755366
H	13.6783025960	-19.1682058347	14.3570062086
H	14.8946550851	-20.1787238798	14.3592772205
O	14.2859992499	-16.8820600463	12.9511251021
H	14.6065177994	-15.9448147059	12.8547711796
H	13.3613613499	-16.8732101656	13.2981797332
O	16.4433286844	-20.3608896173	12.0953335158
H	16.9149022102	-20.4412974723	11.2251293790
H	15.7404227767	-21.0215315227	11.9988897354
O	20.0338441109	-14.5996096839	14.1071350076
H	20.7401917882	-15.2428655845	13.9293379729
H	20.5070091805	-13.7748694443	14.3401431571
O	16.8099795480	-18.0174156557	13.9228529099
H	17.3731390373	-17.2565830332	13.6655215099
H	17.4635635826	-18.7469246722	14.0370781631
O	18.7713641994	-16.4575376331	12.4837062495
H	19.4355976281	-17.1742202315	12.7011359787
H	18.0993837955	-16.8821893031	11.8736976502

PS (Model-5)

N	14.4594977247	-8.3087247323	9.7641648230
H	13.5739094939	-7.7383643032	9.6681499478
C	15.7295048039	-7.8032233257	9.6367677877
H	15.9557187557	-6.8091338557	9.2520383935
C	14.5539217321	-9.5605319481	10.2471936510
H	13.7023077232	-10.2004898826	10.4814419844
N	15.8365166946	-9.8904855020	10.4242281022
C	16.5821385607	-8.7964299489	10.0423045213
H	17.6672643226	-8.7963618510	10.0987795456
N	17.4968011310	-9.7768162146	15.0677061278
H	17.4860087351	-8.9950959762	15.7332546706
C	17.8835893855	-11.0672827666	15.3613406401
H	18.2999889896	-11.3842919635	16.3162556034
C	16.9320111139	-9.7823094124	13.8415558025
H	16.5736556527	-8.8841226360	13.3413157576
N	16.9213311533	-11.0090766396	13.3404088444
C	17.4967577410	-11.8239805049	14.2835875014
H	17.6033199444	-12.8930947386	14.1317304602
C	20.7795279044	-10.2875501968	12.2705792413
H	21.3787820204	-10.1606256417	11.3573356492
H	20.2757720036	-9.3304218208	12.4658937772
C	19.7436134695	-11.3908738772	11.9846236466
O	19.9780981123	-12.5685171766	12.4015550086
O	18.7426951440	-11.0330180202	11.3253437406
H	21.4360359391	-10.5618203801	13.1356894899
C	20.2809529957	-15.7537938507	5.2228573115
H	19.8318893126	-15.0481834939	4.5254552578
C	21.4110821008	-16.5109142239	5.0314539827
H	22.0564224799	-16.6120831423	4.1612566408
N	19.7875778158	-15.9764947727	6.4879671505
H	18.5235264601	-14.9628262420	7.2199486719
N	21.6160627652	-17.1941248896	6.2099106064
H	22.4238588286	-17.8232938602	6.4452367489
C	20.6111029115	-16.8487329048	7.0511165027
H	20.4815825316	-17.2785473842	8.0411023840
N	22.8693826018	-16.6353719909	10.5176966937
H	23.2413397175	-17.3963325487	9.8962069330
C	23.6432694618	-15.7101738770	11.1636857557
H	24.7171102864	-15.6399719841	11.0216261232
C	21.5933730539	-16.4354455638	10.8814779327
H	20.7707581064	-17.0615719787	10.5451325517
N	21.4959084788	-15.3979036006	11.7096532165
C	22.7782315201	-14.9229030730	11.8821276291
H	22.9957307757	-14.0452831271	12.4876950063
C	13.3424440172	-13.3325637556	13.7337860753
H	12.9660379786	-14.3639948717	13.8226213594
H	13.7305277190	-13.0582819202	14.7300817253
C	14.5294477563	-13.3184465398	12.7694148717
O	14.7849750660	-12.3689257458	12.0238337759
O	15.2602308840	-14.3696397157	12.9160009450
H	12.4974516671	-12.6447553834	13.4954426488
Zn	16.6695015370	-11.5277775941	11.2648281207
Mg	19.5655738096	-14.5549103342	12.1117498605
Mg	15.4298021716	-18.4626783658	12.3400808141
C	16.7112114642	-14.1504883444	6.8804357102
H	16.1433736321	-13.3046454842	7.2932592463
H	16.1361097763	-15.0687442941	7.0958721118
O	17.9888863454	-14.1682211015	7.5057994928
P	17.6159800585	-14.2000737806	10.1679079490
O	16.9647827555	-12.8737318641	9.7786301637
O	19.1174745772	-14.4666477829	10.0806950043
O	16.6677463311	-15.4101539377	9.6806618721
P	16.8070905572	-17.0640600734	9.6857888921
O	16.8232421407	-17.4716751725	11.1899426710
O	17.9697373968	-17.5137702090	8.8547754395
O	15.3721161887	-17.3949702972	9.0689761338
P	14.4099179821	-18.7927465805	9.2265517742
O	14.2718915268	-18.9286404422	10.7948265364
O	13.1036907463	-18.4385825296	8.5485240695
O	15.1943337815	-19.9573197194	8.6268324894
H	16.8136879530	-14.0160549044	5.7882372046
O	17.4861926137	-14.2700312251	11.8224822359
H	16.5424201136	-14.3492461611	12.2378887103
O	14.3219667366	-19.6285180389	13.8146896488
H	13.6711270170	-19.1559709295	14.4069087200
H	14.8811994445	-20.1763561354	14.4107371137
O	14.3079348519	-16.8656667655	13.0599823379
H	14.6085121374	-15.9269001953	12.9756139173
H	13.3710674456	-16.8663538018	13.3755855163

O	16.4164790337	-20.3668508401	12.1352472934
H	16.9174508293	-20.4508513691	11.2814249627
H	15.6757473181	-20.9741835763	11.9828490076
O	20.1393593749	-14.6732711186	14.2211164144
H	20.8618223466	-15.3129150078	14.1136664000
H	20.5766520903	-13.8397976102	14.4961467352
O	16.8416306294	-18.0476941070	13.9842742727
H	17.4387274191	-17.3153753149	13.7183816761
H	17.4668470413	-18.8027192766	14.0955191847
O	18.8626214254	-16.5595900093	12.5873109157
H	19.5175374764	-17.2982933913	12.7594323746
H	18.1735363546	-16.9348762423	11.9637219831

RS (Model-6)

N	14.6047428062	-8.5390650035	9.9226127656
H	13.7243511311	-7.9650018669	9.8361835477
C	15.8724592484	-8.0255229733	9.7922985372
H	16.0878418153	-7.0328520036	9.3881076763
C	14.7157014831	-9.8042153137	10.3862682145
H	13.8694291232	-10.4502041438	10.6233776567
N	15.9973699235	-10.1367011592	10.5452564546
C	16.7306462137	-9.0285084238	10.1771384931
H	17.8151375591	-9.0257331643	10.2529743806
N	17.5083845109	-9.7676093481	15.1665091537
H	17.4342392424	-9.0006112308	15.8459783269
C	18.0147063371	-11.0235966175	15.4331537514
H	18.4304152377	-11.3321949670	16.3890346672
C	16.9571319907	-9.8046512845	13.9307631923
H	16.5061262464	-8.9391987945	13.4470248771
N	17.0725426802	-11.0112452514	13.4051543460
C	17.7144676684	-11.7877990339	14.3346825062
H	17.9266992829	-12.8312723032	14.1223552661
C	20.8645108340	-10.1831149552	12.2977677002
H	21.4713449783	-9.9588465143	11.4077250258
H	20.2712476545	-9.2862245381	12.5249772272
C	19.9474290963	-11.3647745329	11.9163309363
O	20.3346488588	-12.5367460498	12.2393080547
O	18.9251568091	-11.0860514991	11.2611184489
H	21.5226234453	-10.4410767788	13.1687796059
C	20.2514418454	-15.7391823042	5.3319029762
H	19.7643513550	-14.9852811920	4.7175490273
C	21.3662309210	-16.4898039931	5.0725384030
H	21.9916031732	-16.5444496702	4.1822609469
N	19.8330332230	-16.0809725544	6.6007541591
H	19.0134909366	-15.6911336011	7.1006930214
N	21.5984186461	-17.2594425771	6.1958622680
H	22.4279709889	-17.8977120060	6.3820624092
C	20.6524364590	-17.0065498868	7.1007998293
H	20.5265368346	-17.5098817096	8.0539574442
N	22.6561753216	-16.6412702897	10.5095768427
H	23.0549310188	-17.4083550579	9.9162891173
C	23.4060412107	-15.6884927275	11.1508114639
H	24.4804047913	-15.5970581557	11.0080595797
C	21.3622221114	-16.4177992512	10.8011698411
H	20.5474995267	-17.0576435669	10.4732323062
N	21.2342064713	-15.3388698610	11.5637669692
C	22.5073863356	-14.8588295009	11.7789313326
H	22.6772021754	-13.9349488917	12.3263342517
C	13.3367832718	-13.4396658945	13.7334157571
H	12.9287160124	-14.4590782717	13.8254375008
H	13.7241838621	-13.1699877867	14.7310966113
C	14.5377597380	-13.4593320599	12.7736037669
O	14.8201723449	-12.4803458568	12.0704127868
O	15.2221964475	-14.5336403860	12.8898400897
H	12.5138646364	-12.7272181852	13.4875029551
Zn	16.8463958227	-11.8860598942	11.3964179691
Zn	19.3855366390	-14.3410114355	11.8724927805
Mg	15.3795983097	-18.4569502495	12.1993660452
C	16.4599255899	-14.2452008252	6.8843149617
H	15.9505071045	-13.3499985399	7.2667445930
H	15.8162405452	-15.1215556360	7.0678550229
O	17.7312617605	-14.4107370578	7.5603007755
P	17.6576148310	-14.2239303015	9.2246000511
O	16.9872351403	-12.8793726036	9.4169543007
O	19.0782736912	-14.5151188808	9.6840439299
O	16.6229049460	-15.4171377701	9.6302638395
P	16.7543748788	-17.0575250855	9.5124691836
O	16.8125827052	-17.5873754292	10.9607339448
O	17.9085351510	-17.4094714380	8.5976181498
O	15.3297897279	-17.4010327698	8.8721022075
P	14.3717621909	-18.8078812745	9.0628095513
O	14.2635467158	-18.9586004186	10.6223576975
O	13.0548807676	-18.4586935419	8.4025536300
O	15.1548778516	-19.9615188241	8.4342163432
H	16.6454109740	-14.1221060190	5.8053976101
O	17.5886266915	-13.6646492002	12.0209465224
H	16.8337090374	-14.2202115686	12.3005398423
O	14.2701957452	-19.6395143733	13.6785416584
H	13.6524543856	-19.1533103687	14.2961335912
H	14.8453896832	-20.1842121625	14.2635042759
O	14.1859624314	-16.8886886388	12.7902135546
H	14.5493856711	-15.9494051839	12.8434863453
H	13.2952480016	-16.9043302784	13.2129783008
O	16.4567850897	-20.3169005302	12.0357802506
H	16.8896114071	-20.3886263654	11.1469908039
H	15.8217813093	-21.0481722153	12.0124735514
O	20.0706785166	-14.5572980896	14.1824730927
H	20.7659815090	-15.1817911273	13.9305119857
H	20.5215946482	-13.6935077009	14.2358913657
O	16.7316565601	-17.9680884202	13.8534234539
H	17.3178288720	-17.2133917069	13.5963360412
H	17.3767728299	-18.7006765942	13.9820961540
O	18.6355358497	-16.4656209973	12.5272285066
H	19.3374380628	-17.1522325631	12.7161540266
H	18.0462478450	-16.8760824411	11.8412713876

TS (Model-6)

N	14.5288600352	-8.3925155045	9.8260416391
H	13.6433104702	-7.8229234472	9.7359139973
C	15.7954202582	-7.8780657692	9.6976313910
H	16.0123493042	-6.8838320663	9.3040280195
C	14.6361762921	-9.6509016165	10.2979577865
H	13.7901100492	-10.2999349931	10.5275392233
N	15.9200321706	-9.9763281824	10.4669129373
C	16.6551268456	-8.8722840222	10.0924674626
H	17.7403048057	-8.8664963945	10.1530804666
N	17.4809808299	-9.8863705598	15.0372719725
H	17.4369294950	-9.0990022281	15.6948502076

C	17.9521446636	-11.1476816077	15.3343223838
H	18.3666076948	-11.4355014403	16.2990194318
C	16.9343237160	-9.9315564708	13.8023902078
H	16.5189707295	-9.0612869052	13.2965074633
N	17.0146981225	-11.1548747817	13.3007983079
C	17.6313667480	-11.9294631000	14.2508167469
H	17.8215229580	-12.9873129959	14.0972430206
C	20.8203167489	-10.2583211180	12.2549917494
H	21.4199859854	-10.0788659785	11.3505267334
H	20.2692817959	-9.3316584066	12.4696455761
C	19.8455312881	-11.4069104724	11.9283035942
O	20.1538346919	-12.5739300748	12.3356414589
O	18.8415656086	-11.1059934756	11.2513413897
H	21.4825537759	-10.5234698269	13.1197080657
C	20.2136421722	-15.5267097863	5.4599270218
H	19.7725314824	-14.8100783845	4.7688991195
C	21.3080734571	-16.3299792006	5.2548782201
H	21.9297701465	-16.4740953851	4.3705132259
N	19.7386577986	-15.7375440249	6.7390660727
H	18.6672398264	-15.0611560450	7.3421611597
N	21.5028537800	-17.0209963341	6.4318153885
H	22.2955271279	-17.6842276247	6.6400045731
C	20.5357714121	-16.6450801349	7.2941031397
H	20.3964344060	-17.0627056339	8.2851359832
N	22.7654883155	-16.6666353921	10.5841510061
H	23.1539018819	-17.4437057452	9.9936392815
C	23.5200104149	-15.6947126638	11.1854997500
H	24.5919608867	-15.6091644549	11.0324858541
C	21.4835324558	-16.4707581844	10.9251914021
H	20.6742835658	-17.1364662531	10.6368684272
N	21.3652691859	-15.3900208221	11.6902371320
C	22.6354650414	-14.8803425978	11.8493441396
H	22.8186985556	-13.9595877041	12.3979922880
C	13.4205557272	-13.4038093460	13.6462796775
H	13.0321933437	-14.4295529769	13.7485023478
H	13.8318320405	-13.1274135195	14.6326968191
C	14.5920803428	-13.4052714613	12.6533027153
O	14.8316755144	-12.4421713923	11.9135423495
O	15.3176514596	-14.4566955558	12.7669686463
H	12.5773576497	-12.7060829278	13.4243144864
Zn	16.7640455734	-11.7141326065	11.2070905986
Zn	19.5009231899	-14.4634483681	11.9159605804
Mg	15.4137229172	-18.4798322823	12.2735640605
C	16.6135661797	-14.3370406448	7.0191924133
H	16.0092746384	-13.5134969876	7.4195984767
H	16.0635810800	-15.2796055730	7.1746836630
O	17.8659576039	-14.3439161562	7.7368817817
P	17.6720074974	-14.1560196196	9.7039364553
O	16.9948508850	-12.7909071817	9.4541050709
O	19.1790316897	-14.4631747675	9.8271271369
O	16.6204258426	-15.4127333145	9.6611407492
P	16.7730470991	-17.0525088854	9.6106600112
O	16.8022123748	-17.5194379251	11.0924293594
O	17.9426068936	-17.4577963499	8.7564441393
O	15.3467213480	-17.4049468430	8.9808559132
P	14.3948913593	-18.8111047359	9.1531868907
O	14.2705413848	-18.9579512883	10.7176308227
O	13.0815058316	-18.4613218759	8.4857116338
O	15.1818889505	-19.9667945730	8.5380824733
H	16.7767608566	-14.1652307748	5.9388490431
O	17.5235525855	-13.9971368567	11.5024551168
H	16.6727927113	-14.3281095239	11.9266099258
O	14.3104028711	-19.6499305436	13.7492053644
H	13.6703697704	-19.1707115472	14.3485882812
H	14.8800689943	-20.1884778893	14.3444416164
O	14.2795456559	-16.8816781851	12.9355799171
H	14.6156288238	-15.9479533150	12.8589385016
H	13.3577440113	-16.8695482288	13.2893954496
O	16.4489588510	-20.3569258397	12.0935369303
H	16.9159486730	-20.4359710641	11.2208523552
H	15.7557762642	-21.0289577074	12.0065050689
O	19.9947570285	-14.6177012011	14.2050616534
H	20.6641468527	-15.2924662418	14.0139751801
H	20.5007956073	-13.7931320120	14.3462968883
O	16.7919767765	-18.0131924427	13.9278720721
H	17.3633752174	-17.2526637277	13.6743990873
H	17.4431675643	-18.7436663788	14.0456547643
O	18.7074967020	-16.4679759712	12.5476175531
H	19.3917202085	-17.1720922074	12.7451526197
H	18.0679017348	-16.8833980062	11.8985763616

RS (Model-7)

N	14.6144946683	-8.4850390400	9.8714232364
H	13.7328965839	-7.9157199707	9.7771707518
C	15.8816428675	-7.9683102659	9.7570182533
H	16.0993623587	-6.9740358885	9.3599944338
C	14.7261629721	-9.7514918405	10.3327669127
H	13.8770193521	-10.3990960384	10.5550737342
N	16.0050837692	-10.0852592914	10.5076895140
C	16.7363062612	-8.9716308193	10.1492333623
H	17.8204885480	-8.9554835541	10.2362649134
N	17.5155492358	-9.7569813849	15.1876880929
H	17.4514232293	-8.9844038060	15.8612344131
C	17.9892109080	-11.0227868777	15.4677700252
H	18.4062052415	-11.3297570982	16.4239986888
C	16.9602130386	-9.7947403942	13.9528645408
H	16.5308265342	-8.9222562942	13.4620881718
N	17.0421389193	-11.0084703640	13.4371668417
C	17.6652302068	-11.7904824766	14.3776488718
H	17.8494231516	-12.8417893268	14.1755853570
C	20.8014356325	-10.2154740650	12.3344539809
H	21.3976289823	-9.9893497532	11.4378600908
H	20.2013011619	-9.3231550252	12.5623492259
C	19.8979971617	-11.4120266628	11.9684728645
O	20.2884937250	-12.5759550854	12.2982254739
O	18.8692275876	-11.1523368650	11.3068057045
H	21.4716483517	-10.4610877732	13.1995870005
C	20.2516572005	-15.7625140534	5.3627509651
H	19.7550543700	-15.0086068268	4.7561255659
C	21.3735022456	-16.4994952200	5.0939159463
H	21.9945498258	-16.5441939965	4.2000871802
N	19.8432308016	-16.1177517221	6.6314646162
H	19.0209823257	-15.7487975932	7.1371074016
N	21.6194372706	-17.2739779935	6.2110488326
H	22.4548933572	-17.9086327478	6.3866693658
C	20.6753431273	-17.0377419650	7.1217588894
H	20.5614372636	-17.5480818304	8.0728125225
N	22.7406420030	-16.7004087472	10.4963254371
H	23.1384456638	-17.4691194626	9.9036559587
C	23.4807427719	-15.7315994795	11.1229005277
H	24.5553714329	-15.6371077943	10.9914763690

C	21.4458445629	-16.4928086341	10.8039788217
H	20.6407404463	-17.1561290331	10.4984504002
N	21.3075023114	-15.4105329273	11.5578832122
C	22.5754902484	-14.9115358579	11.7542402364
H	22.7418921761	-13.9836110859	12.2970425348
C	13.4375290000	-13.3148664792	13.7513277886
H	13.0766545918	-14.3197925104	14.0220570525
H	13.9104565948	-12.8880018012	14.6530130116
C	14.5335710348	-13.4532565019	12.6798057142
O	14.8811954081	-12.4734589374	12.0001042592
O	15.0873896647	-14.6000115811	12.6754818552
H	12.5645062374	-12.6677464123	13.4909708131
Mg	19.3498570911	-14.4848873583	11.8986604809
Fe	16.8614289618	-11.9234453620	11.3827821180
Mg	15.3299154909	-18.5135978261	12.1838350046
C	16.4427493960	-14.2335044257	6.9180441219
H	15.9366286928	-13.3374435424	7.3030917349
H	15.7969208022	-15.1085502651	7.0988138727
O	17.7156855038	-14.4081033724	7.5934123943
P	17.6334704872	-14.2505134496	9.2510160549
O	16.9857497710	-12.8956279621	9.4737785917
O	19.0457214776	-14.5595171918	9.7342470089
O	16.5882104144	-15.4312263177	9.6453290931
P	16.7191839522	-17.0767826211	9.5159674536
O	16.7692605064	-17.6158629660	10.9605122358
O	17.8809365610	-17.4149958570	8.6060845253
O	15.2996954996	-17.4092507678	8.8639088051
P	14.3405346746	-18.8221513390	9.0328123912
O	14.2355854410	-18.9962139054	10.5883039612
O	13.0233452910	-18.4619094861	8.3797424638
O	15.1264538535	-19.9619665650	8.3839201046
H	16.6278365539	-14.1077770487	5.8395406628
O	17.5550587275	-13.7045944767	12.0881360221
H	16.7829579938	-14.2814184403	12.2375271842
O	14.2188953172	-19.6965608863	13.6533382448
H	13.6174564666	-19.2043167773	14.2824504119
H	14.7950667543	-20.2497694641	14.2297748846
O	14.1119410835	-16.9640264357	12.7669181667
H	14.4233436782	-16.0072833546	12.7233378270
H	13.2239803774	-16.9825117572	13.1965627031
O	16.4705963202	-20.3305763248	12.0168481251
H	16.8690172933	-20.3955127703	11.1115876815
H	15.9346184304	-21.1364043783	12.0646737497
O	20.0928962541	-14.5631264751	14.0993068055
H	20.8641538541	-15.1291210477	13.9478788034
H	20.4414582908	-13.6501182263	14.0403101891
O	16.6758815171	-18.0237549349	13.8494827017
H	17.2593054798	-17.2661259944	13.6064257693
H	17.3255090947	-18.7536584359	13.9713878833
O	18.6511426110	-16.5824145215	12.5134285934
H	19.3532548602	-17.2689752553	12.7032922594
H	18.0445900283	-16.9943270777	11.8420649965

References

1. Morris, E. R.; Caswell, S. J.; Kunzelmann, S.; Arnold, L. H.; Purkiss, A. G.; Kelly, G.; Taylor, I. A. Crystal structures of SAMHD1 inhibitor complexes reveal the mechanism of water-mediated dNTP hydrolysis. *Nat. Commun.* **2020**, *11*, 3165.

2. Morris, E. R.; Kunzelmann, S.; Caswell, S. J.; Purkiss, A. G.; Kelly, G.; Taylor, I. A. Probing the Catalytic Mechanism and Inhibition of SAMHD1 Using the Differential Properties of Rp- and Sp-dNTPαS Diastereomers. *Biochemistry* **2021**, *60*, 1682-1698.

3. Klemm, B. P.; Sikkema, A. P.; Hsu, A. L.; Horng, J. C.; Hall, T. M. T.; Borgnia, M. J.; Schaaper, R. M. High-resolution structures of the SAMHD1 dGTPase homolog from Leeuwenhoekiella blandensis reveal a novel mechanism of allosteric activation by dATP. *J. Biol. Chem.* **2022**, *298*, 102073.

4. Bridwell-Rabb, J.; Kang, G.; Zhong, A.; Liu, H.-w.; Drennan, C. L. An HD domain phosphohydrolase active site tailored for oxetanocin-A biosynthesis. *Proc. Natl. Acad. Sci. U.S.A.* **2016**, *113*, 13750-13755.

5. Abramson, J.; Adler, J.; Dunger, J.; Evans, R.; Green, T.; Pritzel, A.; Ronneberger, O.; Willmore, L.; Ballard, A. J.; Bambrick, J.; Bodenstein, S. W.; Evans, D. A.; Hung, C.-C.; O'Neill, M.; Reiman, D.; Tunyasuvunakool, K.; Wu, Z.; Žemgulytė, A.; Arvaniti, E.; Beattie, C.; Bertolli, O.; Bridgland, A.; Cherepanov, A.; Congreve, M.; Cowen-Rivers, A. I.; Cowie, A.; Figurnov, M.; Fuchs, F. B.; Gladman, H.; Jain, R.; Khan, Y. A.; Low, C. M. R.; Perlin, K.; Potapenko, A.; Savy, P.; Singh, S.; Stecula, A.; Thillaisundaram, A.; Tong, C.; Yakneen, S.; Zhong, E. D.; Zielinski, M.; Žídek, A.; Bapst, V.; Kohli, P.; Jaderberg, M.; Hassabis, D.; Jumper, J. M. Accurate structure prediction of biomolecular interactions with AlphaFold 3. *Nature* **2024**, *630*, 493-500.

6. Zimmerman, M. D.; Proudfoot, M.; Yakunin, A.; Minor, W. Structural Insight into the Mechanism of Substrate Specificity and Catalytic Activity of an HD-Domain Phosphohydrolase: The 5′-Deoxyribonucleotidase YfbR from Escherichia coli. *J. Mol. Biol.* **2008**, *378*, 215-226.

7. van Staalduinen, L. M.; McSorley, F. R.; Schiessl, K.; Séguin, J.; Wyatt, P. B.; Hammerschmidt, F.; Zechel, D. L.; Jia, Z. Crystal structure of PhnZ in complex with substrate reveals a di-iron oxygenase mechanism for catabolism of organophosphonates. *Proc. Natl. Acad. Sci. U.S.A.* **2014**, *111*, 5171-5176.

8. Song, X.; Liu, J.; Wang, B. Emergence of Function from Nonheme Diiron Oxygenases: A Quantum Mechanical/Molecular Mechanical Study of Oxygen Activation and Organophosphonate Catabolism Mechanisms by PhnZ. *ACS Catal.* **2022**, *12*, 2009-2022.

9. Zhao, C.; Chen, H. Mechanism of Organophosphonate Catabolism by Diiron Oxygenase PhnZ: A Third Iron-Mediated O–O Activation Scenario in Nature. *ACS Catal.* **2017**, *7*, 3521-3531.

10. Brown, P. M.; Caradoc-Davies, T. T.; Dickson, J. M. J.; Cooper, G. J. S.; Loomes, K. M.; Baker, E. N. Crystal structure of a substrate complex of myo-inositol oxygenase, a di-iron oxygenase with a key role in inositol metabolism. *Proc. Natl. Acad. Sci. U.S.A.* **2006**, *103*, 15032-15037.

11. Klabunde, T.; Sträter, N.; Fröhlich, R.; Witzel, H.; Krebs, B. Mechanism of Fe(III) – Zn(II) Purple Acid Phosphatase Based on Crystal Structures. *J. Mol. Biol.* **1996**, *259*, 737-748.

12. Alberto, M. E.; Marino, T.; Ramos, M. J.; Russo, N. Atomistic details of the Catalytic Mechanism of Fe(III)−Zn(II) Purple Acid Phosphatase. *J. Chem. Theory Comput.* **2010**, *6*, 2424-2433.

13. Jo, S.; Kim, T.; Iyer, V. G.; Im, W. CHARMM-GUI: A web-based graphical user interface for CHARMM. *J. Comput. Chem.* **2008**, *29*, 1859-1865.

14. Lee, J.; Cheng, X.; Swails, J. M.; Yeom, M. S.; Eastman, P. K.; Lemkul, J. A.; Wei, S.; Buckner, J.; Jeong, J. C.; Qi, Y.; Jo, S.; Pande, V. S.; Case, D. A.; Brooks, C. L., III; MacKerell, A. D., Jr.; Klauda, J. B.; Im, W. CHARMM-GUI Input Generator for NAMD, GROMACS, AMBER, OpenMM, and CHARMM/OpenMM Simulations Using the CHARMM36 Additive Force Field. *J. Chem. Theory Comput.* **2016**, *12*, 405-413.

15. Lopata, A.; Jambrina, P. G.; Sharma, P. K.; Brooks, B. R.; Toth, J.; Vertessy, B. G.; Rosta, E. Mutations Decouple Proton Transfer from Phosphate Cleavage in the dUTPase Catalytic Reaction. *ACS Catal.* **2015**, *5*, 3225-3237.

16. Berta, D.; Gehrke, S.; Nyíri, K.; Vértessy, B. G.; Rosta, E. Mechanism-Based Redesign of GAP to Activate Oncogenic Ras. *J. Am. Chem. Soc.* **2023**, *145*, 20302-20310.
17. Rosta, E.; Nowotny, M.; Yang, W.; Hummer, G. Catalytic Mechanism of RNA Backbone Cleavage by Ribonuclease H from Quantum Mechanics/Molecular Mechanics Simulations. *J. Am. Chem. Soc.* **2011**, *133*, 8934-8941.
18. Liao, R.-Z.; Himo, F.; Yu, J.-G.; Liu, R.-Z. Theoretical Study of the RNA Hydrolysis Mechanism of the Dinuclear Zinc Enzyme RNase Z. *Eur. J. Inorg. Chem.* **2009**, *2009*, 2967-2972.
19. Lassila, J. K.; Zalatan, J. G.; Herschlag, D. Biological Phosphoryl-Transfer Reactions: Understanding Mechanism and Catalysis. *Annu. Rev. Biochem.* **2011**, *80*, 669-702.
20. López-Canut, V.; Ruiz-Pernía, J. J.; Castillo, R.; Moliner, V.; Tuñón, I. Hydrolysis of Phosphotriesters: A Theoretical Analysis of the Enzymatic and Solution Mechanisms. *Chem. Eur. J.* **2012**, *18*, 9612-9621.
21. Li, Z.; Wu, Y.; Feng, L.-J.; Wu, R.; Luo, H.-B. Ab Initio QM/MM Study Shows a Highly Dissociated SN2 Hydrolysis Mechanism for the cGMP-Specific Phosphodiesterase-5. *J. Chem. Theory Comput.* **2014**, *10*, 5448-5457.
22. Mulashkina, T. I.; Kulakova, A. M.; Khrenova, M. G. Molecular Basis of the Substrate Specificity of Phosphotriesterase from Pseudomonas diminuta: A Combined QM/MM MD and Electron Density Study. *J. Chem. Inf. Model.* **2024**, *64*, 7035-7045.
